# Gut microbiota predicts body fat change following a low-energy diet: a PREVIEW intervention study

**DOI:** 10.1186/s13073-022-01053-7

**Published:** 2022-05-23

**Authors:** Ching Jian, Marta Paulino Silvestre, Danielle Middleton, Katri Korpela, Elli Jalo, David Broderick, Willem Meindert de Vos, Mikael Fogelholm, Mike William Taylor, Anne Raben, Sally Poppitt, Anne Salonen

**Affiliations:** 1 Human Microbiome Research Program, Faculty of Medicine, University of Helsinki, Helsinki, Finland; 2 NOVA Medical School, NOVA University of Lisbon | Centro de Investigação em Tecnologias e Serviços de Saúde (CINTESIS), Lisbon, Portugal; 3 Human Nutrition Unit, School of Biological Sciences, Department of Medicine, University of Auckland, Auckland, New Zealand; 4 School of Biological Sciences, University of Auckland, Auckland, New Zealand; 5 Department of Food and Nutrition, Faculty of Agriculture and Forestry, University of Helsinki, Helsinki, Finland; 6 Department of Nutrition, Exercise and Sports, University of Copenhagen and Steno Diabetes Center, Copenhagen, Denmark

## Abstract

**Background:**

Low-energy diets (LEDs) comprise commercially formulated food products that provide between 800 and 1200 kcal/day (3.3–5 MJ/day) to aid body weight loss. Recent small-scale studies suggest that LEDs are associated with marked changes in the gut microbiota that may modify the effect of the LED on host metabolism and weight loss. We investigated how the gut microbiota changed during 8 weeks of total meal replacement LED and determined their associations with host response in a sub-analysis of 211 overweight adults with pre-diabetes participating in the large multicentre PREVIEW (PREVention of diabetes through lifestyle intervention and population studies In Europe and around the World) clinical trial.

**Methods:**

Microbial community composition was analysed by Illumina sequencing of the hypervariable V3-V4 regions of the 16S ribosomal RNA (rRNA) gene. Butyrate production capacity was estimated by qPCR targeting the butyryl-CoA:acetate CoA-transferase gene. Bioinformatics and statistical analyses, such as comparison of alpha and beta diversity measures, correlative and differential abundances analysis, were undertaken on the 16S rRNA gene sequences of 211 paired (pre- and post-LED) samples as well as their integration with the clinical, biomedical and dietary datasets for predictive modelling.

**Results:**

The overall composition of the gut microbiota changed markedly and consistently from pre- to post-LED (*P* = 0.001), along with increased richness and diversity (both *P* < 0.001). Following the intervention, the relative abundance of several genera previously associated with metabolic improvements (e.g., *Akkermansia* and *Christensenellaceae* R-7 group) was significantly increased (*P* < 0.001), while flagellated *Pseudobutyrivibrio*, acetogenic *Blautia* and *Bifidobacterium* spp. were decreased (all *P* < 0.001). Butyrate production capacity was reduced (*P* < 0.001). The changes in microbiota composition and predicted functions were significantly associated with body weight loss (*P* < 0.05). Baseline gut microbiota features were able to explain ~25% of variation in total body fat change (post–pre-LED).

**Conclusions:**

The gut microbiota and individual taxa were significantly influenced by the LED intervention and correlated with changes in total body fat and body weight in individuals with overweight and pre-diabetes. Despite inter-individual variation, the baseline gut microbiota was a strong predictor of total body fat change during the energy restriction period.

**Trial registration:**

The PREVIEW trial was prospectively registered at ClinicalTrials.gov (NCT01777893) on January 29, 2013.

**Supplementary Information:**

The online version contains supplementary material available at 10.1186/s13073-022-01053-7.

## Background

To combat the obesity epidemic and its comorbidities such as type 2 diabetes (T2D) [1], energy-restricted diets have been at the forefront of weight management and glucose control. Low-energy diets (LEDs) represent one of the most effective options for weight management [2], with established efficacy for weight loss, but which recently have also proven to be highly successful in normalizing glycaemia in high-risk obese individuals with T2D [3, 4]. However, the success of diet-induced weight loss may vary considerably between individuals and the underlying factors are largely unclear. Mounting evidence suggests that the gut microbiota, one of the most salient features contributing to physiological inter-individual variability [5], is implicated in obesity [6] and influences the host’s metabolic response to diet [7]. In mice, depletion of the gut microbiota nullified the metabolic improvements, especially the decrease in body weight, following energy restriction [8].

The gut microbiota is inextricably linked with the quantity and quality of nutrients in the diet, as gut microbes mainly rely on host diet composition to obtain metabolic substrates [9]. Subsequently, the gut microbiota exerts its impact on host physiology by producing microbial metabolites (e.g., short-chain fatty acids, SCFA) and microbial structural components (e.g., lipopolysaccharides and flagella) that modulate the metabolism of lipids, cholesterol, glucose [10] and inflammatory response [11].

While an association between the gut microbiota and obesity exists, the tripartite interaction between the microbiota, energy restriction and host metabolic response remains little studied in humans. In the context of energy-restricted diets such as LEDs, existing interventions have failed to generate a consensus on changes in the gut microbiota during diet-induced weight loss [12]. For example, Ott and colleagues reported a 4-week LED (3.4 MJ/day) had no effect on the overall gut microbiota [13], whereas another German study by Frost et al. more recently documented distinct shifts of microbiota composition and diversity during a 6-week LED intervention [14]. Variable outcomes regarding specific bacterial taxa affected by LED have been reported in other LED interventions [15–20]. Similarly, the few studies exploring the feasibility of predicting diet-induced weight modulation using baseline features of the gut microbiota arrived at divergent conclusions [7, 21]. This inconsistency is likely due to small sample size (as few as 5 participants), differences in bacterial profiling techniques, population, ethnicity of participants and the intervention diets [12, 22, 23], which preclude generalization of the results. Recently, a re-analysis of omics data collected in two Danish dietary interventions introducing whole grain-rich or low-gluten diets found that inclusion of data on gut microbiota and urine metabolites significantly improved the classification accuracy for weight loss responders [24]. Nevertheless, the definition for weight loss success or responders could vary arbitrarily across studies.

Due to the potential malleability and inter-individual variance in the microbiota, the gut microbiota has increasingly become the focus of precision nutrition, whereby personalized responses to diet predicted and dietary advice tailored to the individual [23]. Therefore, a better understanding of the changes and contribution of the gut microbiota to inter-individual variability in response to a LED may improve the effectiveness of weight-loss interventions.

PREVention of diabetes through lifestyle Intervention and population studies in Europe and around the World (PREVIEW) was a multi-centre, 3-year lifestyle intervention in overweight adults with pre-diabetes conducted in 8 countries that aimed to decrease the incidence of T2D. We have previously reported the clinical outcomes from the first phase of PREVIEW, where eligible adult participants followed an 8-week total meal replacement LED [25]. The LED was accompanied by significant weight loss and associated improvements in anthropometry (e.g., body mass index (BMI) and total body fat) and metabolic parameters (e.g., fasting plasma glucose (FPG)), with gender-specific changes [25]. Here we study the gut microbiota from a subset of participants in the PREVIEW trial, from Finland and New Zealand (*N* = 211), by Illumina sequencing of the 16S rRNA gene. We compared pre- and post-LED differences in the composition and function of the gut microbiota and to determine whether baseline microbiota configuration is predictive of host metabolic response to the LED.

## Methods

### Study participants and design

**Fig. 1:**
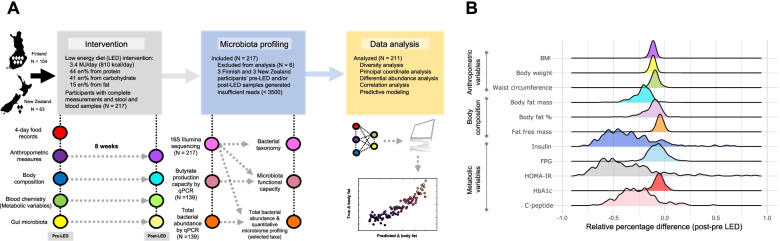
Overview of the study and variation of host variables. **A** Schematic overview of the study design. **B** Density plots showing inter-individual variation in host variables in response to the LED. The density plots display the distribution of the observed data (relative changes in host variables). The density function reflects the estimated underlying continuous probability from which the observed data have been sampled. BMI, body mass index; HOMA, homeostasis model for assessment of insulin resistance; FPG, fasting plasma glucose

PREVIEW was a multi-centre randomized controlled trial (RCT) based on a 3-year lifestyle intervention for T2D prevention across 8 countries, which comprised 2 intervention phases. Phase 1 is an 8-week, weight-loss phase using a formula LED intended to induce weight loss of ≥8% to qualify for the next phase. Phase 2 is a 148-week randomized lifestyle intervention that focuses on diet, physical activity and behaviour modification for maintenance of weight loss [26]. The study population of PREVIEW consisted of 2224 adults with overweight or obesity (BMI≥25 kg/m^2^) and pre-diabetes (according to the American Diabetes Association (ADA) criteria [27]), aged between 25 and 70 years. Overweight men and women with pre-diabetes were eligible for inclusion. Participants were recruited via advertisements in newspapers and newsletters, radio and television advertisements/interviews and by contacting primary and occupational health care providers [25]. The first participant was enrolled on June 1, 2013, and the last participant was enrolled on February 27, 2015. The last participant visit was in March 2018. Participants self-reported not being engaged in competitive sports, with stable body weight (±5 kg) for at least 2 months prior, and no current glucose medications or changes in prescribed medications for 3 months prior to sample collection. Exclusion criteria included diagnosed T2D, other significant diseases including cardiovascular, liver, gastrointestinal, or kidney disease, malignancy, bariatric or any major surgical procedure in the previous 3 months, pregnancy, or breastfeeding. The PREVIEW primary outcome was incidence of T2D at 3 years; secondary outcomes were incidence of T2D at 2 years, gut microbiota analysis and all relevant clinical and biochemical parameters related to metabolic control, at different time-points (8 weeks, 2 years and 3 years). The analysis described in the present study is based on the LED phase of PREVIEW derived from a multi-ethnic cohort recruited from the University of Auckland (UoA), New Zealand, and the University of Helsinki (HEL), Finland (total *N* = 217), from whom paired faecal samples were available (baseline/pre- and post-LED; Fig. 1A). None of the participants reported antibiotic use 3 months prior to or during the LED. As the microbiota analysis involved two of the eight PREVIEW study centres, we termed the present study a PREVIEW sub-study herein for clarity. Detailed methods for the LED intervention were described previously [25]. Briefly, all participants were provided with total meal replacement sachets from Cambridge Weight Plan® (Northants, UK) for the 8 weeks duration. In total, the LED provided an estimated 3.4 MJ/day (810 kcal/day), of which 44 energy % (en%) was from protein, 41 en% from carbohydrate and 15 en% from fat. The total dietary fibre content of the LED was 13.3 g/day (participants’ pre-LED intake of dietary fibre was 22.3 g±7.5 (mean±SD)). Participants were advised that psyllium fibre could be used in case of gastrointestinal side effects, mainly constipation. A maximum of 400 g of non-starchy vegetables could be consumed, such as tomatoes, cucumber and lettuce, making the total energy content approximately 4 MJ (1000 kcal). Participants were advised to avoid intense physical activity and maintain current activity levels during the LED intervention. The work of PREVIEW is carried out in full compliance with the relevant requirements of the latest version of the Declaration of Helsinki (59th WMA General Assembly, Seoul, Korea, October 2008), and the ICH-GCP, The International Conference on Harmonisation (ICH) for Good Clinical Practice to the extent that this is possible and relevant. The study protocol was approved by the Ethical Committees of participating countries (Health and Disability Ethics Committee in New Zealand, ref. 14/191 and Medical Ethical Committees of the Hospital District of Helsinki and Uusimaa and HUCH in Finland, ref. 171/13/03/00/2013). All participants provided written informed consent prior to commencing screening procedures in clinic. All information obtained during the trial is handled according to local regulations and the European Directive 95/46/CE (directive on protection of individuals with regard to the processing of personal data and on the free movement of such data). The PREVIEW trial was prospectively registered at ClinicalTrials.gov (NCT01777893). Additional information can be found on the PREVIEW website (https://preview.ning.com/).

### Sample collection and clinical outcome measurements

Detailed protocols for clinical sample collection and outcome measurements have been described elsewhere [25, 26]. Before and after the 8-week LED, participants attended the research clinic on clinical investigation days (CIDs) for anthropometry measurements, collection of fasting blood samples and delivery of faecal samples and 4-day food records to assess habitual diet. The faecal samples were collected at home, frozen immediately at – 20 °C in the home freezer and taken in frozen form to the study centres within 1–3 days of collection, then stored at – 80 °C until processing. Body weight and height were measured in duplicate wearing light clothing, without shoes and after voiding the bladder. Body composition including fat mass and fat-free mass was assessed by bioelectrical impedance analysis, BIA (Finland, InBody720 Body Composition Analyzer, Biospace Co., Ltd, Seoul, Korea) and dual-energy X-ray absorptiometry, DXA (New Zealand, iDXA, model DPX+, software version 3.6y, GE-Lunar, Madison, WI). Fasting venous blood samples were collected for laboratory measurements, including FPG, HbA1c, insulin and C-peptide. Laboratory analyses were performed on an Architect ci8200 integrated system (Abbott Laboratories, Abbott Park, Illinois, USA). The Homeostasis Model for Assessment (HOMA) was calculated as a proxy for insulin resistance (IR). The equation used was HOMA-IR = fasting insulin (mU/L) × FPG(mmol/L))/22.5.

### DNA extraction and 16S rRNA gene amplicon sequencing

DNA was extracted from all faecal samples with a previously described repeated bead-beating method that efficiently extracts bacterial community DNA including hard-to-lyse Gram-positive bacteria [28]. The quantity of extracted DNA was assessed using the Qubit Fluorometer (Thermo Fisher Scientific). Bacterial community composition was analysed by sequencing the PCR-amplicons of hypervariable V3-V4 regions of the 16S ribosomal RNA (rRNA) gene using primers 341F/785R and Illumina MiSeq (New Zealand, *N* = 126) or Illumina HiSeq (Finland, *N* = 308) as previously described [29]. The comparability of sequences generated by the two Illumina platforms was validated by evaluating two artificial communities and nine PREVIEW samples that were sequenced by both MiSeq and HiSeq (Additional file 1: Fig. S1A&B).

### Quantification of butyrate production capacity and absolute bacterial abundance by quantitative PCR (qPCR)

Based on sample availability, the butyryl-CoA:acetate CoA-transferase gene and total bacterial abundance were quantified using faecal DNA for a subset of 139 participants (pre- and post-LED sample *N* = 278) with the degenerate primers BCoATscrF/R and the universal primers 331F/797R by qPCR, respectively. The qPCR assays have been described in detail previously [30, 31] and were performed in triplicate on a BioRad iCycler iQ thermal cycler system (BioRad, Hercules, CA) with HOT FIREPol® EvaGreen® qPCR Mix Plus (Solis BioDyne, Tartu, Estonia). For quantification of the butyryl-CoA:acetate CoA-transferase gene, the mean threshold cycle (Ct) per sample (after excluding triplicates with Ct values that differed >0.5) was used as a proxy for the abundance of the target gene. For quantification of total bacterial abundance, the 10-log-fold standard curves ranging from 10^2^ to 10^7^ copies were produced using full-length amplicons of 16S rRNA gene of *Bifidobacterium longum* to convert the threshold cycle (Ct) values into the average estimates of target bacterial genomes present in 1 g of faeces (copy numbers/g of wet faeces) in the assays [31]. The absolute abundances of individual bacterial taxa were estimated as previously described [31], adjusting for 16S rRNA gene copy-number variation using the rrnDB database [32].

### Data processing and statistical analysis

Demultiplexed reads after adaptor removal were processed using the QIIME2 v.2019.4. pipeline [33]. The high-quality forward reads were truncated to 150 bases and error-corrected using the DADA2 plugin [34] to generate amplicon sequence variants (ASVs). Taxonomic classification was performed using a pre-trained naive Bayes classifier implemented in QIIME2 against the SILVA 132 reference database [35]. Sample pairs from 6 participants (*N* = 12) were excluded from downstream analysis due to one or both of the samples having low reads after processing (<3500 reads), leaving a total of 211 participants with paired samples (*N* = 422) for downstream analysis. Samples meeting quality criteria (*N* = 422) had a mean sequencing depth of 67,453 reads (63,638–71,268, 95% CI). The sequencing files are deposited in the European Nucleotide Archive (https://www.ebi.ac.uk/ena) under accession number PRJEB43667.

To infer the functional contribution of bacterial communities from 16S rRNA gene sequencing data, metagenome prediction was carried out using PICRUSt2 (Phylogenetic Investigation of Communities by Reconstruction of Unobserved States) [36] evaluating KEGG (Kyoto Encyclopedia of Genes and Genomes) pathways [37].

Differential abundance for bacterial taxa or KEGG pathways between time points was identified with the *DESeq2* package [38] accounting for sample pairing. *DESeq2* employs a generalized linear model of counts based on a negative binomial distribution, scaled by a normalization factor that accounts for differences in sequencing depth between samples. Significance testing was then assessed using the Wald test. Non-count variables (anthropometric and biochemical measurements, microbiota diversity and richness) were analysed with Wilcoxon signed-rank test or paired *t*-test for non-normally distributed and normally distributed variables, respectively.

Microbiota richness and Shannon diversity index were estimated using the *vegan* package [39]. Overall microbiota structure was assessed by principle coordinate analysis (PCoA) on beta diversity computed using Bray-Curtis distances, representing the compositional dissimilarity between the samples. Permutational multivariate analysis of variance (PERMANOVA; *adonis* function in the *vegan* package [39]) with Bray-Curtis dissimilarities was used to identify factors contributing to variation in microbiota composition. At baseline, variation in the microbiota was significantly associated with gender (*P* = 0.006), ethnicity (*P* = 0.004) and age (*P* = 0.003). Hence, analyses were performed with and without adjustment for gender, ethnicity and age when applicable. Associations between bacterial taxa or KEGG pathways (post–pre-intervention) and clinical measurements (post–pre-intervention) were assessed using Spearman’s correlation as well as a linear mixed-effects model implemented in the *mare* package [40] for normal and adjusted correlations, respectively.

For prediction of host responses to LED, stepwise regression based on Akaike information criterion (AIC) (*PathModel* function in the *mare* package [40]) was used to select baseline features (microbiota, diet, host physiological variables, or a combination of them) that fit parsimonious models for %change in clinical measurements (post–pre-intervention). The *PathModel* function employs generalized or general linear models (in this case using the function *lm* in R), identifying first the variables that are most significantly associated with the response variable, combining them all into one model and performing stepwise model reduction using the functions *step* and *stepAIC* in the packages *stats* and *MASS* to finally arrive at the best model. To assess model performance based on AIC, we adopted the conventional rules of thumb by comparing the difference between the AICs of two models [41]:
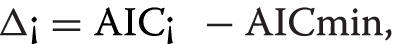


where AIC¡ is the AIC of model ¡ with the second lowest AIC, and AICmin is the model with the lowest AIC among the set of models examined. If Δ¡ < 2, there is substantial support for model ¡, whereas models with Δ¡ > 10 have essentially no support. Considering potential non-linear relationships between the microbiota features and the target variables, the prediction based on the baseline microbiota features was also done by Random Forest regression (*R* package *randomForest* [42]) using the following parameters: ntree = 10001 and mtry = *p*/3, where *p* is the number of input features. We then used repeated cross-validation (5-fold, 10 repetitions) of random forests in the *caret* package [43] in order to evaluate the *R*^2^ of the selected features to predict clinical indices. This method involves repeatedly using a subset of samples as a training set and the remaining samples as the test set to predict the outcome. The importance of each input feature was subsequently ranked according to %increase in mean squared error (%IncMSE). In both approaches, the prediction models based on the microbial features were generated using all prevalent (present in >30% of samples) bacterial genera detected at baseline, Shannon diversity and richness. For the stepwise regression models based on diet, the input features included intake of dietary nutrients; demographic characteristics, anthropometric and metabolic measurements (Table 1) were included in the models based on host physiological variables. For the microbiota and diet-based models, we included potential confounding variables (gender, ethnicity and age) in separate models (adjusted model) to account for confounding effects, since these variables may impact both exposure (e.g., baseline gut microbiota and dietary pattern) and outcome (e.g., change in adiposity).

**Table 1:** Demographic characteristics, anthropometric and metabolic measurements of the study participants pre- and post-LED

Characteristic	pre-LED	post-LED	***P*** value*
**Demographic variables**			
No. of participants	211	–	–
Female, No. (%)	156 (74)	–	–
Age (years), mean (95% CI)	54 (53–55)	–	–
Ethnicity, No. (%)			
Caucasian	194 (92)	–	–
Polynesian	13 (6)	–	–
Asian	3 (1)	–	–
Other	1 (0.4)	–	–
**Anthropometric variables**			
BMI (kg/m^2^), mean (95% CI)	34.1 (33.3–34.9)	30.2 (29.5–30.9)	<0.001
Body weight (kg), mean (95% CI)	95.8 (93.3–98.2)	84.7 (82.5–86.9)	<0.001
Waist circumference (cm), mean (95% CI)	108.6 (106.9–110.2)	98.4. (96.8–100.0)	<0.001
**Body composition**			
Body fat mass (kg), mean (95% CI)	40.1 (38.3–41.8)	31.8 (30.2–33.5)	<0.001
Body fat (%), mean (95% CI)	41.8 (407–42.9)	37.3 (36.0–38.6)	<0.001
Fat-free mass (kg), mean (95% CI)	55.6 (54.1–57.1)	52.9 (51.5–54.3)	<0.001
**Metabolic variables**			
Insulin (mU/L), mean (95% CI)	13.1 (12.1–14.0)	7.9 (7.4–8.4)	<0.001
FPG (mmol/L), mean (95% CI)	6.2 (6.2–6.3)	5.8 (5.7–5.9)	<0.001
HOMA-IR, mean (95% CI)	3.6 (3.4–3.9)	2.0 (1.9–2.2)	<0.001
HbA1c (mmol/mol), mean (95% CI)	37.0 (36.6–37.5)	34.8 (34.3–35.2)	<0.001
C-peptide (pmol/L), mean (95% CI)	908.3 (867.2–949.3)	648.0 (616.9–679.1)	<0.001

*FPG* fasting plasma glucose, *HOMA-IR* homeostasis model assessment of insulin resistance

*P value pre-LED vs post-LED was calculated using paired *t*-test for FPG and body fat % and Wilcoxon signed-rank test for other variables

Statistical analyses were performed with the statistical program *R* version 3.5.0 and RStudio version 0.99.903. *P* values were corrected for multiple comparisons by using the Benjamini-Hochberg procedure (FDR) [44]. *P* values and FDR-adjusted *P* values <0.05 were considered significant.

## Results

Baseline characteristics and post-LED measurements of the 211 participants included in our analysis are summarized in Table 1. Concordant with the findings from the main PREVIEW trial [25, 45], participants lost an average of 11.5% body weight and 22% total body fat during the LED with a significant improvement in all metabolic parameters investigated (Table 1). Seventy-six participants (36%) reverted to normoglycemia (defined as FPG <5.6 mmol/L). Substantial inter-individual variation was found in the LED-induced changes in glucose metabolism-related variables and total body fat (Fig. 1B). Members of the bacterial phylum Firmicutes dominated the baseline (pre-LED) gut microbiota of participants (86%±11; mean±SD), followed by Actinobacteria (9%±9) and Bacteroidetes (2%±3). Verrucomicrobia and Proteobacteria altogether represented on average less than 3% of the microbiota.

### Impact of 8-week LED on gut microbiota

**Fig. 2:**
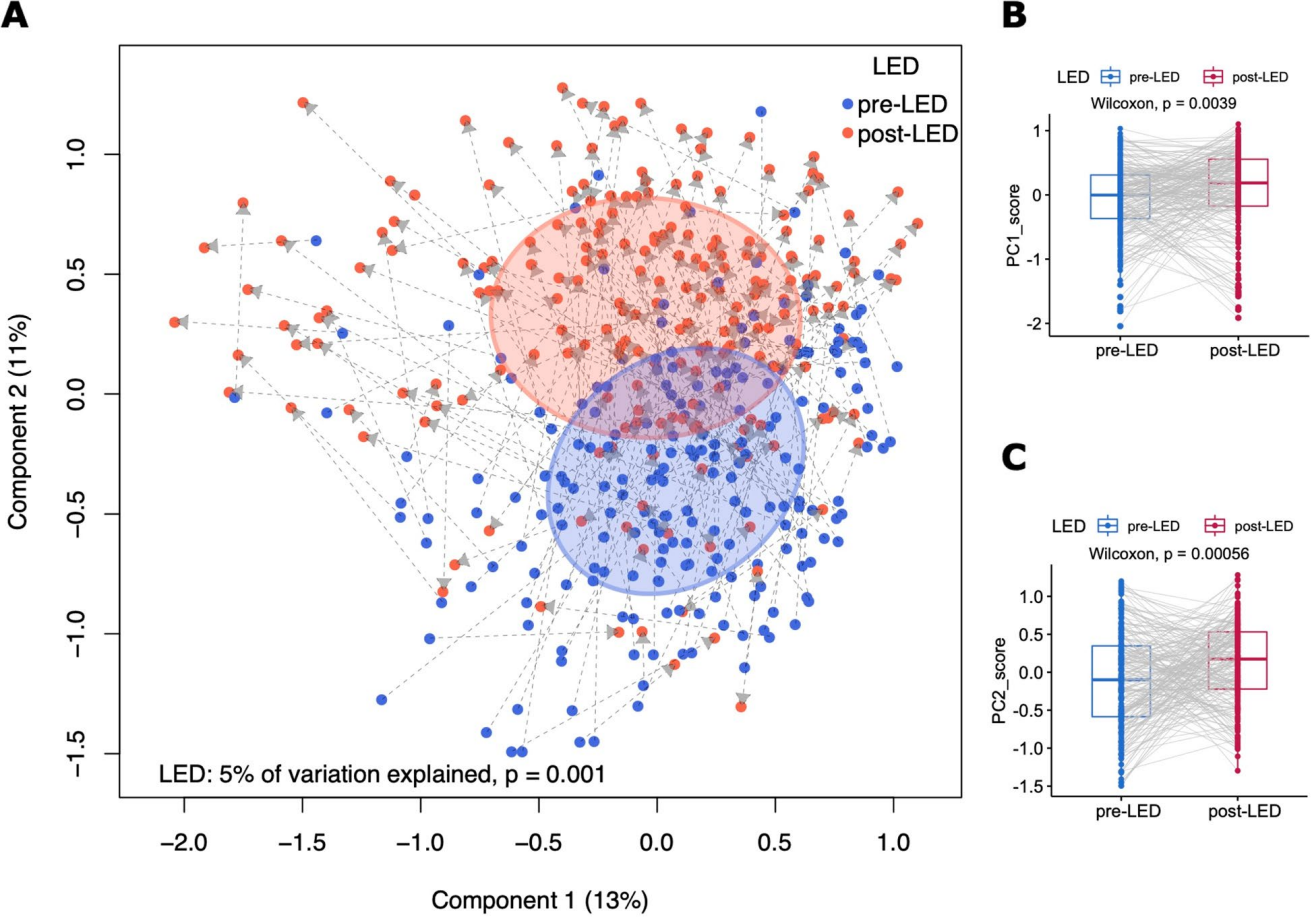
Principal coordinate analysis (PCoA) of microbiota variation in pre-(blue dots) and post-(red dots) LED samples based on Bray-Curtis distances (**A**). Arrows link the baseline pre- and post-intervention sample of each individual, indicating direction of change. The blue and red dispersion ellipses represent standard deviations within the groups of pre- and post-intervention samples, respectively. The principal component (PC) scores of PC1 (**B**) and PC2 (**C**) are plotted by the sampling time points

**Fig. 3:**
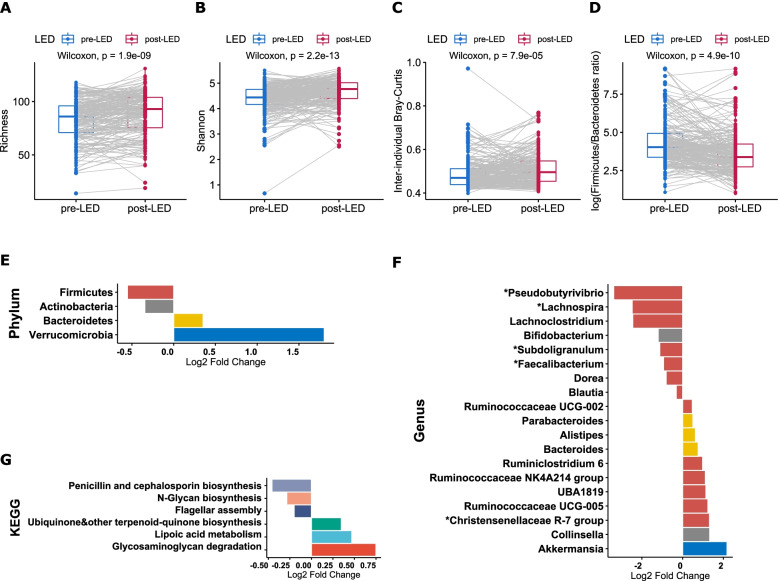
The LED intervention reshapes the overall microbiota structure, alters relative abundances of individual bacterial taxa and predicted functions. Pre- and post-LED **A** richness, **B** diversity within samples (Shannon index), **C** average dissimilarities (beta diversity) estimated by Bray-Curtis distances between participants, and **D** Firmicutes to Bacteroidetes ratio. Differentially abundant **E** phyla, **F** genera (coloured by respective phyla) and **G** KEGG modules following the LED ranked by log-fold change are visualized by divergent bar plots. Only the 15 most abundant genera and KEGG modules are shown in **F** and **G**. Log2 fold change is calculated as post-LED/pre-LED; only significant results (FDR-P < 0.05) are plotted. The genera known to be able to produce butyrate are marked with an asterisk (*) in **I**

The composition of the gut microbiota changed markedly from pre- to post-LED, visualized in strong clustering of the samples by PCoA and reflected in a significant shift in overall phylogenetic makeup between the two time points (*P* = 0.001, PERMANOVA) (Fig. 2A). Both the principal component 1 and 2 scores were significantly higher after LED (*P* < 0.001) (Fig. 2B,C), suggesting a consistent response of the microbiota to the same energy-restricted diet. The LED did not alter total bacterial density, measured by qPCR-based quantification of 16S rRNA gene copies per gram of faeces (Additional file 1: Fig. S2). Microbiota richness and alpha diversity estimated by Shannon index were significantly increased after the intervention (*P* < 0.001, Fig. 3A,B). Inter-individual Bray-Curtis values, representing how different the microbiota compositions are between participants, were significantly increased after LED weight loss (*P* < 0.001) (Fig. 3C). A significant decrease was observed in the ratio between Firmicutes and Bacteroidetes (*P* < 0.001) (Fig. 3D).

Having established pre- vs post-LED differences at the level of the entire bacterial community, we sought to identify specific bacteria that were affected by the LED. At the phylum level, the 8-week LED was accompanied by significant increases in Verrucomicrobia and Bacteroidetes (*P* < 0.001), and concomitant decreases in Actinobacteria and Firmicutes (*P* < 0.001) (Fig. 3H). At the genus level, in addition to *Akkermansia* (*P* < 0.001), the abundances of five genera from family *Ruminococcaceae* (*Ruminococcaceae* UCG-002, *Ruminiclostridium* 6, *Ruminococcaceae* NK4A214 group, UBA1819, *Ruminococcaceae* UCG-005) as well as *Bacteroides*, *Alistipes* and *Christensenellaceae* R-7 group, were increased (*P* < 0.001; Fig. 3I). *Christensenellaceae* R-7 group appeared to form the hub of the post-LED co-occurrence network with other bacterial groups (Additional file 1: Fig. S3). By contrast, *Pseudobutyrivibrio* and some other genera belonging to Firmicutes including other butyrate producers such as *Lachnospira*, *Subdoligranulum* and *Faecalibacterium* but also the acetogen *Blautia*, were significantly decreased (*P* < 0.001, Fig. 3I). There was also a significant decrease in the abundance of *Bifidobacterium* (*P* < 0.001) after the intervention (Fig. 3I). Consistent with the changes in relative abundance, the absolute abundances of *Akkermansia* and *Christensenellaceae* R-7 group were significantly increased, and *Blautia*, *Pseudobutyrivibrio* and *Bifidobacterium* significantly decreased after the LED (all *P* < 0.001) (Additional file 1: Fig. S2).

To understand the functional implications of the observed taxonomic changes, we inferred metagenomes using the PICRUSt2 algorithm. Of 173 KEGG pathways predicted, 6 pathways significantly differed in abundance between the sampling points; these included pathways pertinent to microbial metabolic processes (glycosaminoglycan degradation, lipoic acid metabolism and N-glycan biosynthesis) and the assembly process of flagella (Fig. 3J).

### Post–pre-LED changes in butyrate production capacity

**Fig. 4:**
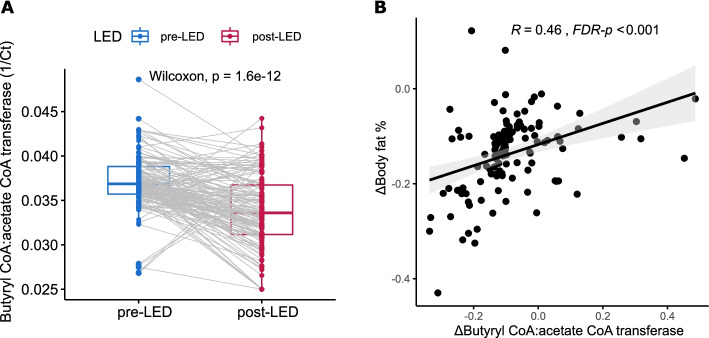
The LED intervention and body fat (%) reduction associated with reduced capacity for butyrate production in the gut microbiota. **A** qPCR quantification of the butyryl-CoA:acetate CoA-transferase gene in pre- and post-LED faecal samples. Data are expressed as 1/mean threshold cycle (Ct). **B** Relative changes (post–pre-LED) in body fat (%) (ΔBody fat %) significantly correlated with relative changes in butyrate production capacity (ΔButyryl CoA:acetate CoA transferase)

As relative abundances of several butyrate-producing bacterial genera were significantly decreased post-LED, we hypothesized that direct quantification of the butyryl-CoA:acetate CoA-transferase gene (responsible for a major route for butyrate production in bacteria) would allow us to gauge changes in butyrate production capacity more precisely. As expected, the post-LED capacity for butyrate production, estimated by qPCR for a subset of participants (*N* = 139) with available samples, was significantly reduced (*P* < 0.001, Fig. 4A). Among all the measured clinical indices (Fig. 1B), change in body fat (%) was significantly and positively associated with butyrate production capacity (FDR-P<0.001, Fig. 4B).

### Associations between changes in the gut microbiota and clinical variables (post–pre-LED)

**Fig. 5:**
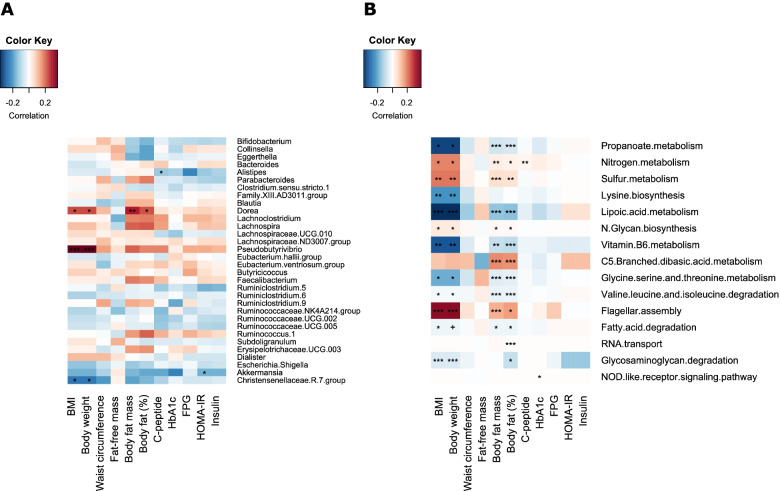
Correlation heatmaps for changes (post–pre-LED) in **A** bacterial genera and **B** KEGG functional modules. For readability of the figures, only prevalent bacterial genera (present in >30% of samples) or functional modules that had at least one significant association with changes in clinical measurements are shown. * FDR-P < 0.05; ** FDR-P < 0.01; *** FDR-P < 0.001

Associations between changes (post–pre diet) in the bacterial genera or predicted functions and clinical indices were first explored by nonparametric correlation analysis to provide an overview of microbiota-host associations (Fig. 5), from which linear mixed-effects models adjusting for demographic variables were fitted to identify significant associations that are potentially generalizable across populations. After the adjustment, changes in BMI and body weight were positively associated with the change in the abundance of *Pseudobutyrivibrio* (estimate = 0.58, FDR-P<0.01) and negatively with *Christensenellaceae* R7 group (estimate = − 0.3, FDR-P=0.03). Change in total body fat mass was consistently positively associated with changes in the relative abundances of *Pseudobutyrivibrio* (estimate=0.16, FDR-P<0.01) and *Dorea* (estimate=0.04, FDR-*P*<0.01). These significant associations were not present at baseline (pre-LED) (Additional file 1: Fig. S4). Changes in BMI had a weak but significant negative association with intra-individual Bray-Curtis distance (Additional file 1: Fig. S5), suggesting that the magnitude of microbiota change is associated with individual weight loss.

With respect to microbiota function, after the adjustment, BMI and body weight remained positively and negatively associated with flagellar assembly (estimate=0.003, FDR-P<0.01) and glycosaminoglycan degradation (estimate = − 0.008, FDR-P<0.01), respectively. These two functional modules were significantly affected by LED as mentioned previously (*P* < 0.001, Fig. 5B). The changes were attributable to changes in the abundance of *Pseudobutyrivibrio* (contributing to flagellar assembly) and *Akkermansia* (contributing to glycosaminoglycan degradation) (Additional file 1: Fig. S6).

### Prediction of host responses to LED using baseline microbiota

**Fig. 6:**
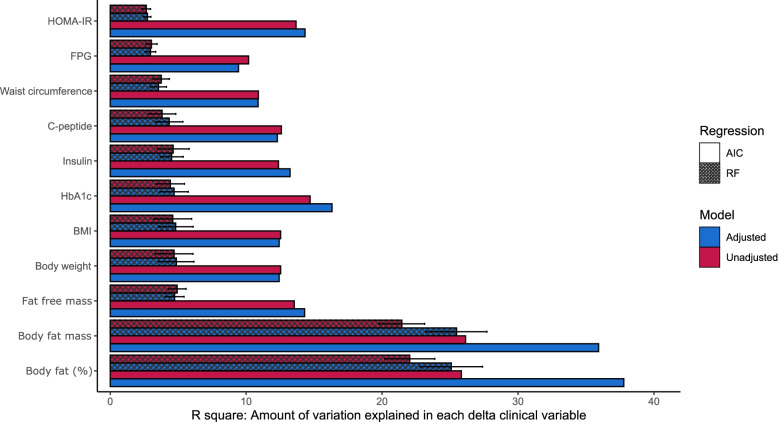
Amount of variation in changes of clinical indices explained by baseline gut microbiota. The bar graph shows the estimated *R*^2^ in Random Forest (RF) and stepwise regressions. The error bars show 95% confidence intervals from repeated cross-validation of the random forests to predict the delta (post–pre-LED) clinical indices. In both regression models, the adjusted model includes demographic variables (age, gender and ethnicity) in addition to microbiota features. The unadjusted model is included as a contrast to showcase the predictive power of gut microbiota features for specific clinical indices without conflating the information related to host clinical characteristics

**Fig. 7:**
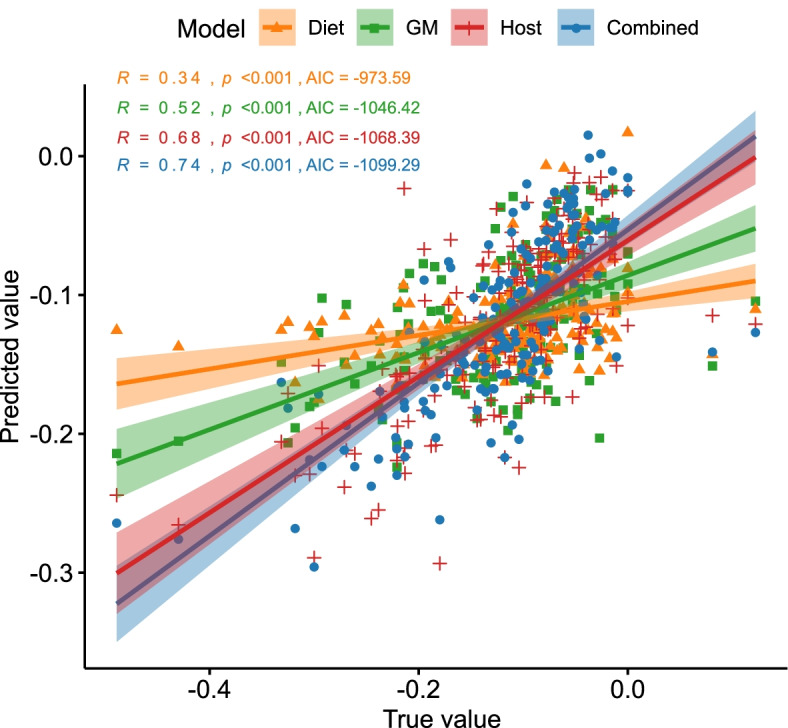
Comparison of the association strengths between the true and predicted total body fat (%) change during the LED based on the four different models. The lines represent the fitted regression lines (Spearman’s rank correlation coefficients displayed at the upper left corner) and the corresponding shaded area represents the 95% confidence intervals for each model. GM, gut microbiota; host, host clinical characteristics including demographic characteristics, anthropometric and metabolic measurements as presented in Table 1

Given the above connection between the microbiota changes and changes in adiposity, we asked whether the extent of host response to LED could be predicted based on an individual’s baseline microbiota. Our results from stepwise and random forest (RF) regressions indicated that baseline features of the gut microbiota explained a significant proportion of variance in both unadjusted and adjusted models (ca. 26–38% in stepwise regression; 22–25% in RF) in %change of total body fat, but not other clinical indices during the LED (Fig. 6). Similar results were obtained by applying the same set of predictors in the Finnish participants only (*N* = 151) (Additional file 1: Fig. S7). The baseline microbiota features predictive of total body fat change are listed in Table 2 for the stepwise regression model and in Additional file 2: Table S1-S2 for the RF regression models. *Erysipelotrichaceae* UCG-003 emerged as consistently predictive of total body fat change in both stepwise and RF regressions and had the strongest correlation with changes in total body fat (Additional file 1: Fig. S8). We next constructed predictive models for total body fat change based on 4 sets of baseline host features, including (1) microbiota-only, (2) diet-only, (3) host clinical characteristics (Table 1) and (4) a combination of 1–3. The prediction based on the combined model outperformed the predictions based on all other models, as the difference in the Akaike information criterion (AIC) value between the best (i.e., combined model) and second best model (i.e., host clinical characteristics) was larger than the conventional cutoff of 10 AIC units for significant model support. The predicted and measured total body fat (%) change based on the combined model had Spearman’s R of 0.74, corresponding to 55% of the variance in total body fat (%) change (*P* < 0.001, Fig. 7). The combined model indicated that the higher the baseline body fat (%), monounsaturated fatty acid intake in the habitual diet, and gut microbiota richness, the less successful was the intervention in terms of fat loss (%). Conversely, a high relative abundance of *Clostridium sensu stricto* 1, *Ruminococcaceae* UCG-003 and *Parabacteroides* at baseline were predictive of increased fat loss (%) during the intervention (Table 2). In the model without adjustment for host characteristics and habitual diet, also *Lactococcus* and an unclassified genus of *Peptostreptococcaceae* were significantly associated with a good response, while *Erysipelotrichaceae* UCG-003 was predictive of poor response. Their effects were, however, explained by the host characteristics, rather than being predictive in their own right.

**Table 2:** Variables retained in the prediction models for relative change in total body fat (%) based on stepwise selection

Variable	Coefficient	SE	***z***	***P***
***Combined***				
Body fat (%)	0.049	0.0048	10.23	<0.001
Gut microbiota richness	0.01	0.005	1.9	0.05
*Clostridium sensu stricto* 1	− 0.007	0.0045	− 1.58	0.1
*Parabacteroides*	− 0.008	0.0046	− 1.8	0.07
FPG (mmol/L)	− 0.01	0.004	− 2.54	0.01
Ruminococcaceae UCG-003	− 0.01	0.0047	− 2.63	0.009
MUFA intake (en%)	0.01	0.0044	2.29	0.02
***Host clinical characteristics***				
Gender	− 0.024	0.017	− 1.443	0.15
Waist circumference (cm)	− 0.522	0.19	− 2.73	0.007
BMI (kg/m^2^)	− 0.01	0.004	− 2.37	0.02
Body fat (%)	0.012	0.002	5	<0.001
FPG (mmol/L)	− 0.017	0.007	− 2.26	0.02
***Gut microbiota***				
Ruminococcaceae UCG-003	− 0.013	0.005	− 2.35	0.02
*Lactococcus*	− 0.01	0.006	− 1.88	0.06
*Parabacteroides*	− 0.013	0.005	− 2.46	0.01
Peptostreptococcaceae_unclassified	− 0.021	0.006	− 3.76	<0.001
Erysipelotrichaceae UCG-003	0.014	0.006	2.56	0.04
***Diet***				
Total fat intake (en%)	− 11.1	6.12	− 1.82	0.07
Total CHO intake (en%)	0.936	0.425	2.21	0.03
Sugars intake (en%)	− 1.02	0.66	− 1.54	0.1
Fibre intake (en%)	− 3.08	2.2	− 1.4	0.16
SFA intake (en%)	11.06	7.57	1.46	0.14
MUFA intake (en%)	14.2	6.43	2.21	0.03
PUFA intake (en%)	14	8.41	1.67	0.1

*BMI* body mass index, *FPG* fasting plasma glucose, *CHO* carbohydrate, *SFA* saturated fat, *MUFA* monounsaturated fat, *PUFA* polyunsaturated fat

## Discussion

Recent preclinical and clinical studies suggest that, along with other host and environmental factors [46, 47], the gut microbiota contributes to individual variability in diet-induced weight loss [7]. Here we present microbiota data derived from PREVIEW, the largest intervention to date in overweight or obese adults with pre-diabetes undertaking an 8-week LED for weight loss. To our knowledge, the participants included in this PREVIEW sub-study represent the largest cohort to date investigating the impact of a commercial total meal replacement LED on the gut microbiota. Our results show that (1) the LED intervention significantly altered the overall community structure, relative and absolute abundances of bacterial taxa and functional potential of the microbiota; (2) changes in the gut microbiota were strongly associated with changes in adiposity-related variables; and ( 3) decrease in body fat during the LED was predicted by the baseline features of the gut microbiota.

We observed drastic shifts in the overall microbiota structure measured by beta diversity after 8-weeks of LED, which is consistent with the previous results from Frost and colleagues’ 6-week energy restriction trial in obese individuals with T2D [14] and Heinsen’s 12-week intervention in obese adults with various chronic diseases [17], both of which used total meal replacements of similar macronutrient composition and energy content (3.4 MJ/day). Similarly, Simões et al. showed significant changes in dominant faecal bacteria following a 6-week very low-energy diet (VLED; providing fewer than 800 kcal/day (3.3 MJ)) in adults with obesity [16]. Moreover, a recent intervention analysing the effects of VLED (2.5 MJ/day) in 30 adults with overweight or obesity and non-alcoholic fatty liver disease (NAFLD) also found that 4 weeks of VLED had a significant impact on the overall gut microbiota [18]. One weight loss study not using total meal replacement products reported that a 6-week high-protein energy-restricted diet significantly increased microbial gene richness that was associated with improved clinical phenotypes in obese and overweight adults [48]. In contrast, many other weight loss interventions with duration ranging from 4 to 12 weeks did not find a significant difference in microbiota structure before and after energy restriction in overweight and obese individuals with a range of metabolic syndrome and NAFLD conditions [13, 15, 19, 20]. Evidence indicates that the microbiota may respond to diet within 1–3 days [49–51], suggesting a 4-week timeframe should have been sufficient to observe changes in the microbiota. The differences are unexpected since most of these trials used a very similar approach to weight loss, namely LED complete meal replacement. The discrepancy may perhaps arise from small differences in macro and micronutrient composition, compliance to the LED, or more likely differences in microbiome methodologies which may be exacerbated in these studies of very small sample size [12, 22]. Interestingly, the microbiota responses to the same LED in the PREVIEW participants were highly consistent, as opposed to personalized microbiota responses to similar foods previously reported in 34 healthy individuals [51].

In contrast to a recent VLED study in 40 post-menopausal women with overweight or obesity [52], we did not observe a reduction in total bacterial density after the 8-week LED. This difference is possibly owing to the different macronutrient compositions of the OPTIFAST® Liquid Diet [52] and the Cambridge Weight Plan® used in our study. Other indices that reflect microbiota structure, namely microbiota richness and alpha diversity, increased significantly following the LED as also reported by previous studies using similar energy-restricted regimes [14, 48]. While greater microbiota diversity does not necessarily imply better health [53], low diversity has been linked to poor metabolic health due to, e.g., loss of metabolically important functional capacity [54]. In comparison with the baseline, the post-LED microbiota can be delineated by (1) reduced fibre-degrading Firmicutes (mainly the *Lachnospiraceae* family as well as other key butyrate producers), (2) decreased relative and absolute abundance of *Bifidobacterium* and (3) concomitant increases in *Akkermansia* and the *Christensenellaceae* family. *Pseudobutyrivibrio* in the *Lachnospiraceae* family was the genus that decreased the most following the LED in our study; this genus has an elusive role as it has been associated with both weight loss [55] and the pro-inflammatory response [56] in animal models.

By inferring metagenomes, we show that reduced *Pseudobutyrivibrio* may have contributed to lower microbiota capacity in flagellar assembly, the changes in which associated positively to that of body weight and fat mass. This is relevant as flagellins are canonical effectors of Toll-like receptor 5 and have been suggested to contribute to obesity [57]. Reductions in *Bifidobacterium* and several butyrate-producing bacteria are not surprising considering these bacteria thrive on indigestible polysaccharides that were scarce during the LED. While decreased *Bifidobacterium* is often associated with metabolic disorders [58], the change in *Bifidobacterium* did not correlate with clinical measurements investigated here in PREVIEW. The observed small reduction of *Blautia* is of interest, as *Blautia* is a highly abundant and acetogenic group and recent studies have shown that the intestinal levels of *Blautia* spp. are increased in patients with T2D as compared to healthy controls [59]. *Akkermansia*, previously shown to be inversely correlated with metabolic derangements [60] and recently shown to improve barrier functions and insulinemia in obese individuals [61], was significantly increased following the LED in PREVIEW. This contributed to the elevated microbiota capacity for glycosaminoglycan degradation, in line with the extensive capacity of *Akkermansia* for genes capable of metabolizing host glycans, e.g., mucins [62] to gain an edge over competitors during scarcity of dietary glycans as also noted in fasting animals [63]. Increases in *Christensenellaceae* R-7 group were likely partly promoted by the adequate protein content in the LED (43.7 E%, 88 g/day), as this bacterial group specializes in protein fermentation and may produce butyrate [64]. Members of the *Christensenellaceae* family have been linked to decreased adiposity [65], which is consistent with our correlation analysis. It has been hypothesized that the protective effect of *Christensenellaceae* against excess adiposity gain involves remodeling the microbial community [64], which is supported by the *Christensenellaceae*-centred co-occurrence network after the LED as well as in a previous study [66].

*Akkermansia* was the only genus found to negatively associate with insulin resistance index (HOMA-IR) during LED, but the significance disappeared after adjusting for demographics. While a recent study in ~1500 Swedes strongly associated insulin resistance and glycemic status, specifically impaired glucose tolerance, with the gut microbiota [67], we found few associations between the gut bacteria and changes in glucose metabolism during the LED. A possible explanation is that one key mechanism by which the gut microbiota mediates glucose metabolism is through colonic fermentation of dietary fibre [68], which was however relatively low in the intervention diet. A recent post hoc analysis of a subgroup in Cotillard and colleagues’ calorie restriction (CR) intervention [48], where the diet was rich in total fibre, found that fibre intake and gut metagenomic species that were interconnected in a biological network had the greatest contribution to CR-induced improvement in insulin sensitivity [69].

The capacity for butyrate production in the post-LED gut microbiota, quantified by qPCR rather than inferred metagenomics, was significantly reduced. This mirrors the results from a recent VLED study [52]. Interestingly, we found that reduced butyrate production capacity was proportional to reduced body fat (%). While butyrate is generally considered anti-obesogenic, some studies reported higher concentrations of butyrate in obese humans than in lean individuals [70]. Greater stool calorie loss, a proxy of decreased nutrient absorption, has been linked to lower circulating [71] and faecal [52] levels of butyrate, suggesting decreased gut microbial capacity in processing nutrients. Our findings therefore lend credence to the new working model where weight loss and metabolic improvements in obese individuals induced by caloric restriction are potentially mediated by impaired nutrient absorption that is associated with gut microbiota changes, including reduced butyrate production [52, 71]. This also implies that the effects of butyrate on body weight control likely depend on host health status and/or dietary conditions. While the observations in the present study should not be taken as evidence of causality or causal mechanisms, the role of butyrate in nutrient absorption under different clinical and nutritional conditions warrants further mechanistic investigation. Moreover, the long-term impact of reduced butyrate production capacity induced by the LED on host health requires clarification.

Our previous studies [72, 73] and data from others [74, 75] have suggested that an individual’s metabolic response to different diets depends partly on baseline features of the microbiota. Recently, the baseline ratio between *Prevotella* and *Bacteroides* was proposed as a predictive biomarker for weight loss [76, 77]. However, the utility of this ratio may be limited by the sparsity of *Prevotella* [78]. A recent Chinese study in 83 participants on a 6-month self-managed dieting program suggests that the baseline gut microbiota is predictive of weight loss [21], whereas body composition and other metabolic markers were not measured. Importantly, BMI or body weight per se is an inadequate indicator for metabolic health [79] or the success of any interventions [80]. As the variance in weight change in our PREVIEW cohort was relatively small due to the fully controlled LED and high compliance, we were able to focus on the variables representing the quality of the weight loss, e.g., body fat loss and lean mass loss. By applying predictive modelling in our current study, we show that the baseline microbiota alone was able to explain a significant proportion of variance (up to 38%) in total body fat (%), but not in BMI or body weight, during LED. Since weight loss entails a reduction in both lean (fat-free) and fat mass, the baseline microbiota likely modifies the LED-driven effect on the fat tissue specifically. Indeed, preclinical and clinical studies have consistently shown that the gut microbiota modulates adipose tissue physiology [65, 81–83]. Of note, the cross-sectional results from the large-scale PREDICT1 study (*N* = 1098) found that visceral fat was more strongly linked to gut microbial composition than BMI [84]. Our findings from the PREVIEW intervention therefore extend on the findings from PREDICT1, highlighting the crucial role of the gut microbiota in adipose tissue physiology. In contrast, the baseline microbiota was not predictive of changes in clinical indices related to glucose metabolism in PREVIEW. Given the lack of association between the gut microbiota and glucose metabolism during weight loss, the LED-driven improvement in glucose metabolism was likely microbiota-independent in our cohort. The baseline relative abundances of *Erysipelotrichaceae* UCG-003 and *Clostridium sensu stricto* 1 were selected as important features for prediction of total body fat change by both feature selection techniques, but their abundances were otherwise unchanged during the LED phase. Analogous findings were reported in a recent Danish study [24] and this again suggests that the LED-induced change in body fat was modified by the baseline microbiota as an effect modifier [23]. *Erysipelotrichaceae* UCG-003 (the top feature in predicting total body fat change in PREVIEW) was recently identified as a key player in a cohort of lean patients with confirmed NAFLD [85], a consequence of increased non-adipose ectopic fat deposition into liver. Members of the *Erysipelotrichaceae* family have also been repeatedly linked to host lipid and cholesterol phenotypes in humans [86] and experimental animals [87, 88]; likewise, *Clostridium sensu stricto* 1 has been associated with high-density lipoprotein (HDL) metabolism in a recent population-based study [89].

For prediction of total body fat change during LED in PREVIEW, the combined model with host physiological, dietary and microbial features had better predictive performance than the model with only host physiological features. This suggests that microbial and habitual dietary features modify host responses to dietary change, rather than acting as mere proxies for the bio-clinical features. Notably, several species of *Parabacteroides*, one of the taxa predictive of fat loss, have been previously shown to reduce obesity in mice [90, 91]. Whether gut microbes influence fat loss via metabolism, or e.g. via eating behaviour [92], is an intriguing question. SCFAs, neurotransmitters and peptides produced by gut microbes are all hypothesized to regulate appetite [93]. Weight loss success during a diet is largely an outcome of behaviour alongside physiology [94]. In this PREVIEW sub-study, we found unexpectedly that the higher the baseline body fat percentage, the poorer the response in terms of fat percentage loss, suggestive of lower compliance and/or more sedentary lifestyle. Taken together, our work and other recent studies [21, 24] demonstrate that data integration using host and microbial features prior to an intervention is able to predict diet-induced metabolic changes over relatively long time spans, while this strategy has only been utilized to successfully predict postprandial responses previously [75, 95].

As inter-individual variation in the gut microbiota is notably high and shaped by various factors such as long-term diet and host genetics [7], small and homogenous cohorts as utilized by previous studies provide little generalizability and applicability. The main strength of our report is therefore a large novel cohort of overweight adults confirmed to be at high risk of T2D from two different. geographical regions and various ethnicities, adhering to a well-controlled and uniform dietary regime for weight loss, rendering the study clinically relevant. Compliance to diet was confirmed by the significant ≥8% body weight loss over 8 weeks. The results presented in this study should nonetheless be interpreted with some limitations in mind, including different methods for total body fat assessment (DXA and BIA) in the two study sites, which may have resulted in differences in absolute body fat mass and fat-free mass. However, we mitigated potential bias to the greatest extent by analysing change from baseline data, making the results from DXA and BIA more comparable [96]. We additionally tested the regression model in the Finnish participants only (body composition assessed using BIA) to ensure consistent findings. While we cannot exclude the possibility that natural temporal fluctuation of the gut microbiota partly contributed to the observed changes, the gut microbiota is known to be rather stable in the absence of extreme external stressors even over a 2-year period [97].

## Conclusions

Obesity and its most prevalent co-morbidity, T2D, could affect half of the world’s adult population by 2030 [98]. By identifying the gut microbiota as an important co-determinant of LED-induced reduction in total body fat, our study lays the foundation for pre-intervention assessment and patient stratification using individual microbiota profiles. A recent study suggests that daily pre-diet gut microbiota variability, termed “plasticity,” is associated with diet-induced weight loss [99]. Hence, integration of baseline microbiota variability by daily sampling prior to weight loss might improve the prediction of total body fat change, which warrants investigation in future studies. In conclusion, an 8-week LED weight loss intervention in adults with overweight and pre-diabetes significantly influenced microbiota structure, functional potential and relative abundance of several bacterial taxa. These correlated with favorable changes in adiposity that can be predicted by baseline features of the gut microbiota.

## Supplementary Information

**Additional file 1.** Supplementary Information consisting of eight supporting figures (Figure S1-S8). A short description of the figure and a figure caption for each figure are given within the file.

**Additional file 2.** Two supporting tables (Table S1-S2) of lists of input predictors for body fat change in random forest regression ranked by feature importance. A table caption for each table is given within the file.

**Additional file 3.** PREVIEW study protocol.

## Data Availability

The sequencing data in this study are available at the European Nucleotide Archive (ENA) under accession number PRJEB43667 (https://www.ebi.ac.uk/ena/browser/view/PRJEB43667) [100]. All codes are fully accessible from the referenced sources in the program R.

## Abbreviations

LED: Low-energy diet
T2D: Type 2 diabetes
NAFLD: Non-alcoholic fatty liver disease
BIA: Bioelectrical impedance analysis
DXA: Dual-energy X-ray absorptiometry
BMI: Body mass index
FPG: Fasting plasma glucose
HOMA-IR: Homeostatic Model Assessment of Insulin Resistance
HbA1c: Glycated hemoglobin
SCFA: Short-chain fatty acid
KEGG: Kyoto Encyclopedia of Genes and Genomes
rRNA: Ribosomal RNA
PCoA: Principal coordinate analysis
FDR-P: FDR-adjusted *P* value
RF: Random forest
