# Supplementary material for: Gut microbiota predicts body fat change following a low-energy diet: a PREVIEW intervention study
Source: Genome Med. 2022 May 23;14:54. doi: 10.1186/s13073-022-01053-7 (PMC9125896; doi:10.1186/s13073-022-01053-7)
Supplement: Supplementary file 1 — Additional file 1. Supplementary Information consisting of eight supporting figures (Figure S1-S8). A short description of the figure and a figure caption for each figure are given within the file. [file 13073_2022_1053_MOESM1_ESM.pdf]

Supplementary information

Evaluation of comparability of samples sequenced by Illumina MiSeq and HiSeq

Previous studies utilizing the two Illumina platforms have posited that data generated by MiSeq and HiSeq are highly comparable [1, 2]. We validated the comparability with nine PREVIEW samples and two artificial communities that had pre-determined compositions of known species [3]. The 16S rRNA gene amplicon sequencing was performed following the same protocols described in Materials and Methods.

The principle coordinates analysis (PCoA) plot of nine PREVIEW samples based on Bray-Curtis distance metrices was visualized using EMPeror [4] (**Fig S1A**). No clustering by sequencing technology (squares and circles) was found and permutational multivariate analysis of variance by sequencing technology returned a non-significant p-value (P=0.8), indicating that the microbiota variation in the samples had no association with sequencing technology (MiSeq or HiSeq).

We next compared the taxonomic compositions of two artificial communities (**Fig S1B**). We found that the MiSeq- and HiSeq-derived samples were highly similar in composition and generally recapitulated the expected compositions of references.

<u>Additional file 1: Figure S1</u>

A

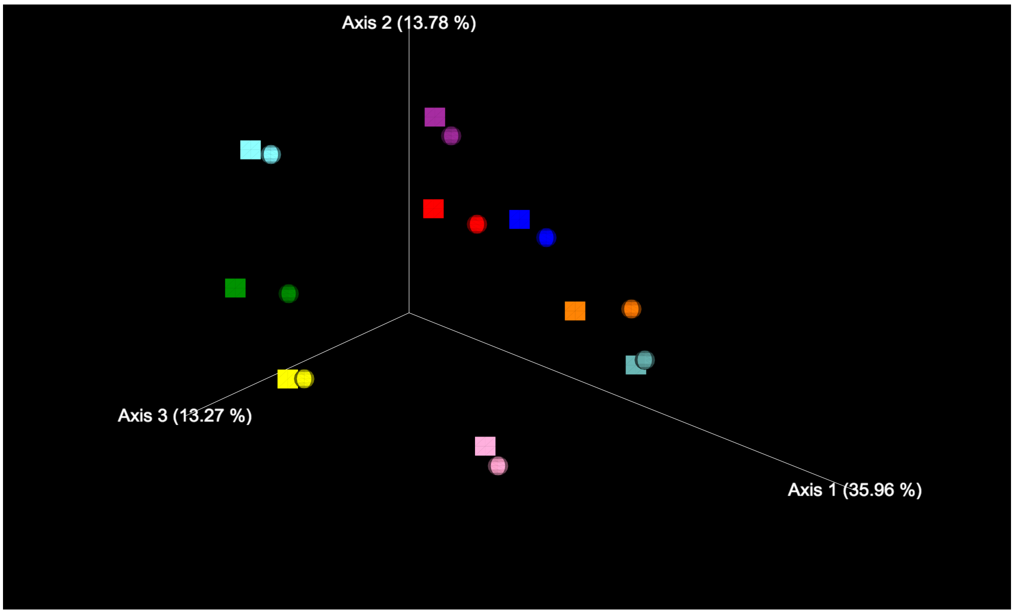

B

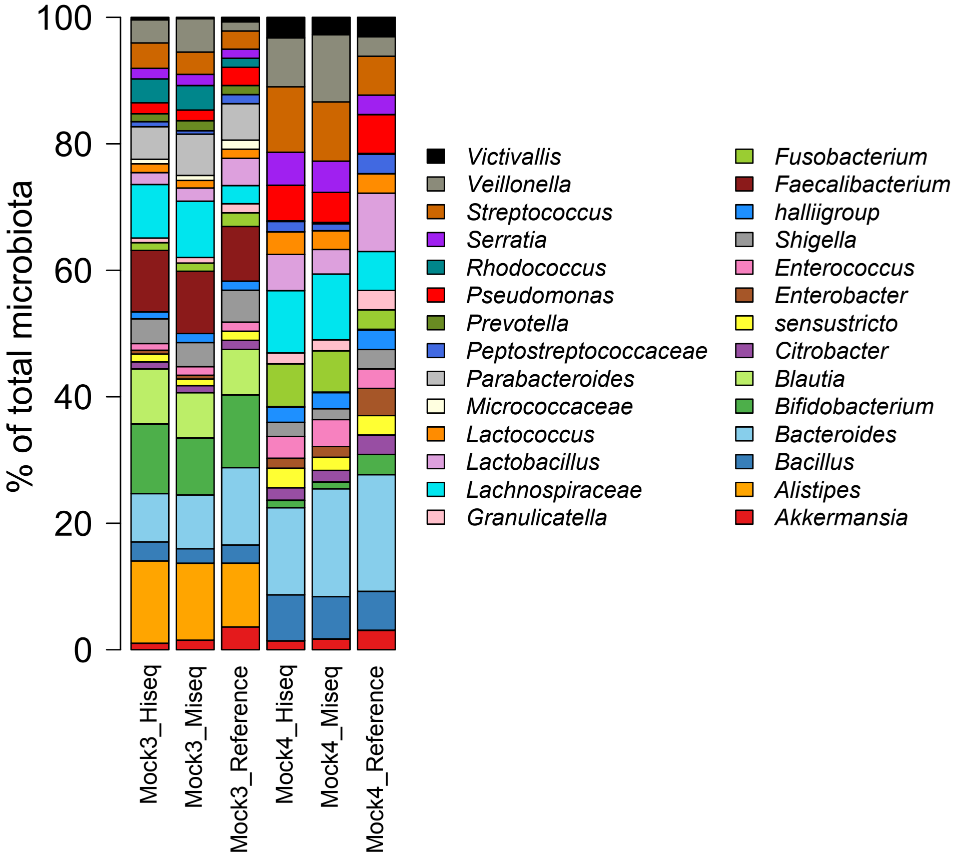

Figure S1. (A) Nine PREVIEW samples processed by MiSeq (squares) and HiSeq (circles) showing comparable community structures. **(B)** Artificial communities (mock community (MC) 3 & 4 from the study by Ramiro-Garcia et al. [3]) sequenced by MiSeq and HiSeq showing comparable compositions.

Absolute quantification of selected LED-associated bacterial taxa by quantitative PCR

<u>Additional file 1: Figure S2</u>

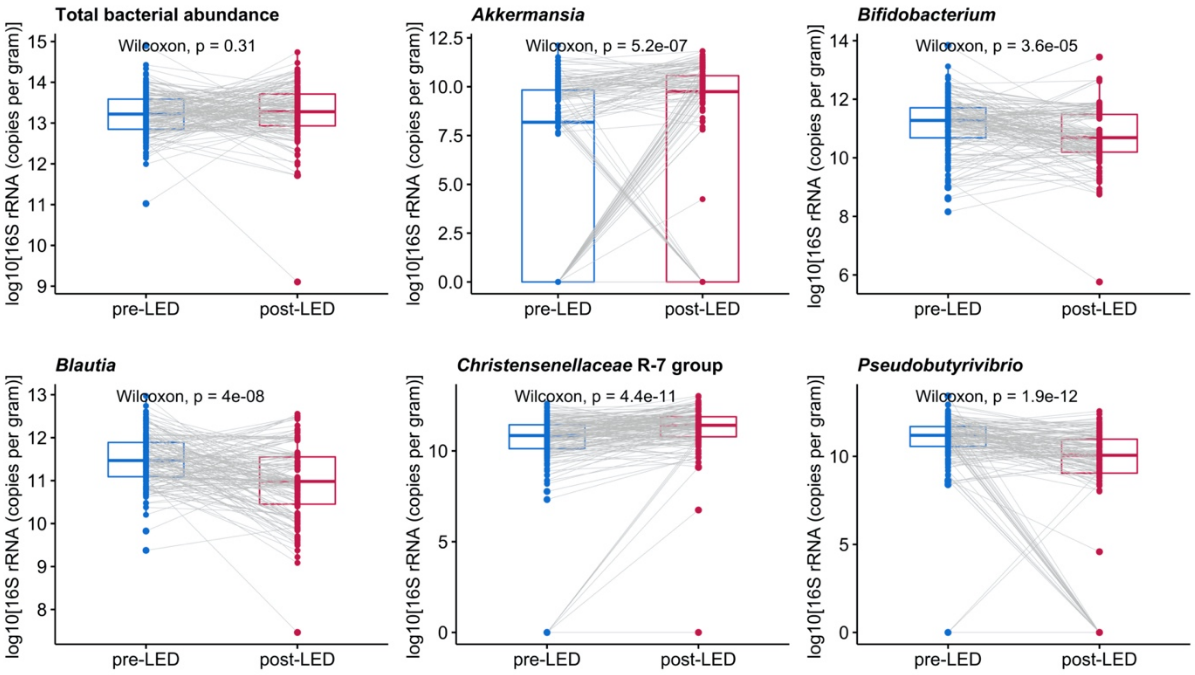

Figure S2. Total bacterial density (expressed as the logarithmic value of 16S rRNA gene copies per g feces) remained unchanged (P=0.31), while significant increases and decreases in the absolute abundances of *Akkermansia*, *Bifidobacterium*, *Blautia, Christensenellaceae* R-7 group, and *Pseudobutyrivibrio* were observed after the LED (all P<0.001). The directions of the changes were consistent with the relative abundance data. The qPCR assays were performed in a subset of 139 participants based on sample availability as described in Methods.

Construction of co-occurrence networks of pre- and post-LED microbiota

Co-occurrence networks (**Figure S3**) were constructed by hierarchical clustering based on significant correlations between bacterial genera (*clusters* function in the *mare* package with default settings).

Additional file 1: Figure S3

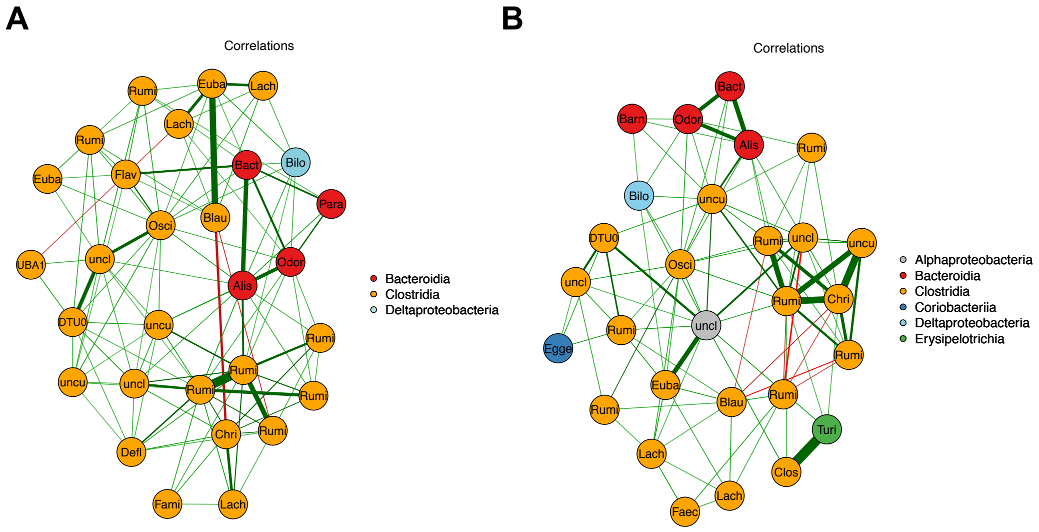

Figure S3. Co-occurrence networks of pre- (**A**) and post-LED (**B**) gut microbiota. *Christensenellaceae* R-7 group (*Chri*) forms a hub of the networks after the LED. For readability only genera with >4 significantly correlating partners were included in the plots.

Baseline correlation between gut microbiota and adiposity

We assessed baseline associations between adiposity and the relative abundances of genera that were significantly associated with weight loss during the LED by fitting a negative binomial regression model (*CovariateTest* function in the *mare* package) while controlling for demographic variables. Intervention site (Finland and New Zealand) was additionally controlled for considering its potential confounding effect for baseline data. The results were consistent with no significant association found between the genera and body weight or body fat mass (FDR-P>0.05) (**Figure S4**).

<u>Additional file 1: Figure S4</u>

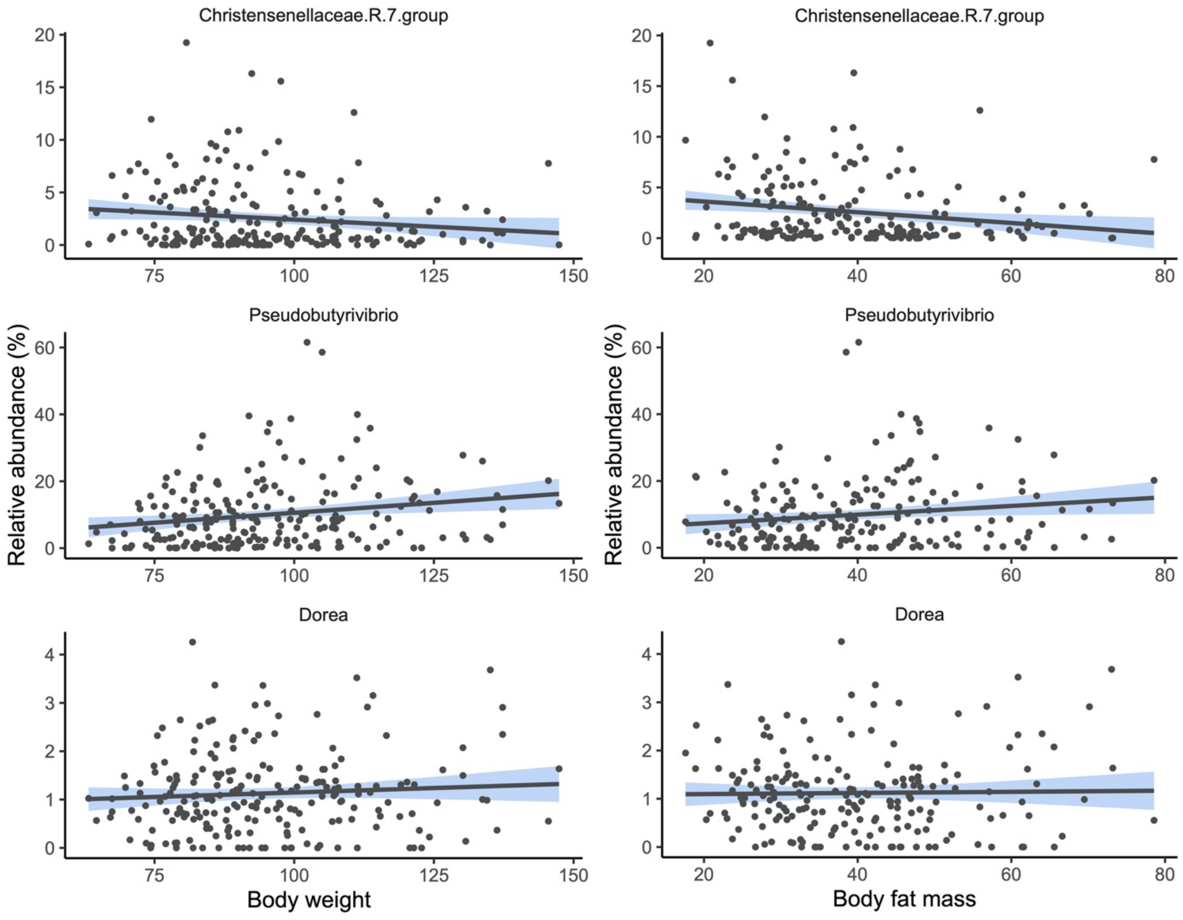

Figure S4. Associations between body weight or body fat mass and selected bacterial genera at baseline.

The correlation between beta diversity and BMI change was assessed by both Spearman's correlation (**Figure S5**) and partial Spearman's test adjusting for demographic variables (p = 0.001, adjusted R-squared = -0.06).

Additional file 1: Figure S5

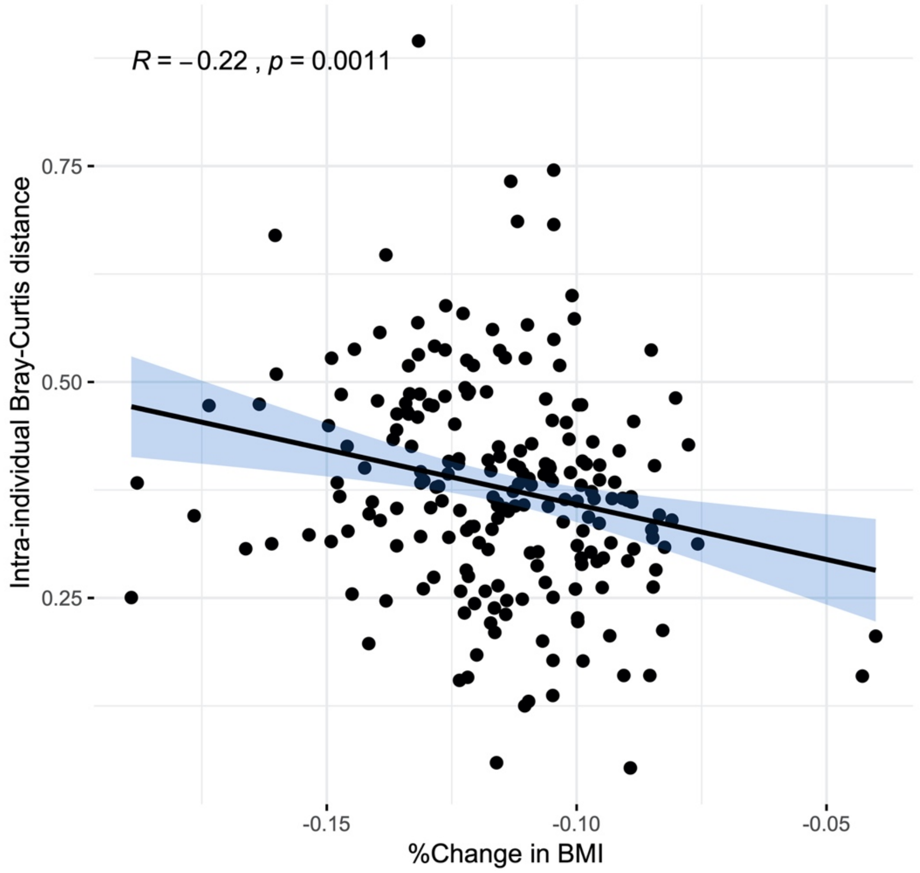

Figure S5. Intra-individual Bray-Curtis (beta diversity) significantly associated with change in BMI.

Contribution of bacterial genera to imputed KEGG functional pathways

We analyzed the contribution of bacterial genera to the two imputed KEGG pathways (Glycosaminoglycan degradation and Flagellar assembly) that were significantly affected by the LED and associated with changes in clinical measurements during the LED. A breakdown of genera contributing to KEGG pathways was obtained by PICRUSt2 where the *-stratified* option was specified within *picrust2_pipeline.py*.

The most changed genera contributing to the significant pre- and post-LED difference in glycosaminoglycan degradation and flagellar assembly were *Akkermansia* (pre-LED=6.9%; post-LED=9.2%) and *Pseudobutyrivibrio* (pre-LED=36.0%; post-LED=13.3%), respectively (**Figure S6**).

Additional file 1: Figure S6

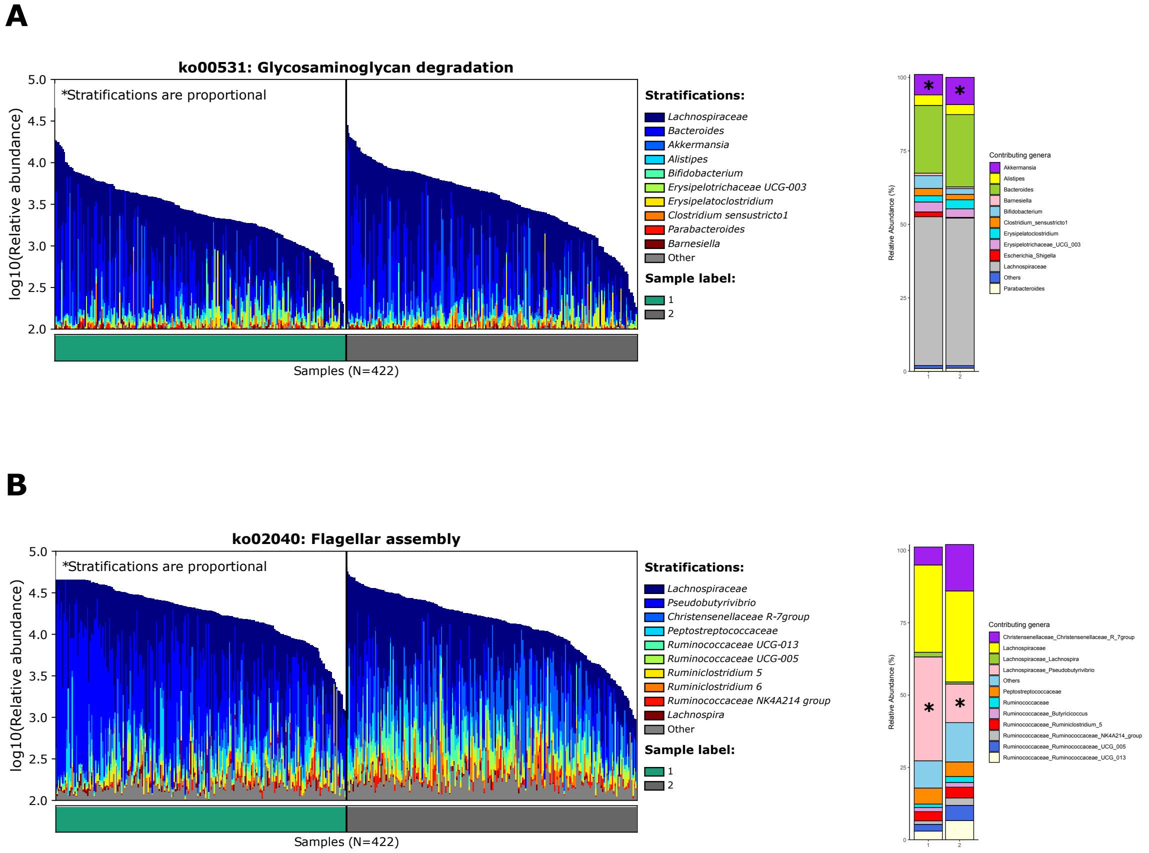

Figure S6. 10 top contributing genera to the KEGG pathways (**A, B**) that were significantly affected by the LED and associated with weight loss (left). The most changed genera contributing to the significant pre- and post-LED difference in the KEGG pathways (**A, B**) (right). Top contributing genera to LED-induced increases or decreases of corresponding function modules are marked with asterisks (*).

Validation of predictive models in Finnish participants

Given body fat was measured using different equipment by Finland and New Zealand, we validated the findings on prediction of host responses using baseline microbiota in the Finnish cohort only (N=151). The prediction models were generated and presented following the same method described in the main text.

Additional file 1: Figure S7

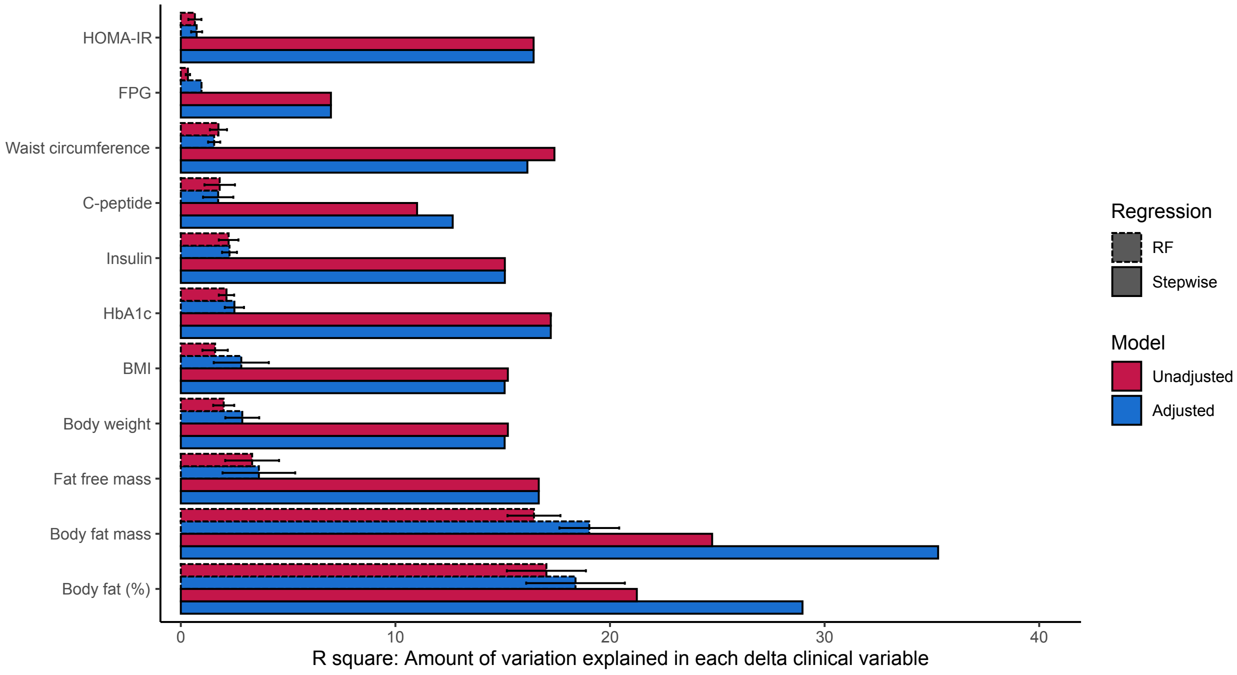

Figure S7. Amount of variation in changes of clinical indices explained by baseline gut microbiota in Finnish participants by Random Forest and stepwise regressions.

Top important features selected by Random Forest regression in linear mixed-effects model

RF-based models are suitable for gut microbiota data as they allow complex interrelationships within predictors and between predictors and the outcome, which however render interpretation challenging [5]. To test potential linear relationships between the selected microbiota features and body fat change, linear regression models (*CovariateTest* function in the *mare* package) were applied to the 10 most important genera selected by RF (**Additional file 2**). Since microbiota features are highly correlated, this method allowed us to assess the linear relationship with %change in adiposity for each feature. In these models, we controlled for the same potential confounding variables as used in the random forests.

Among the genera both selected by RF and linearly associated with body fat change (*Lachnospiraceae* ND-3007 group, *Eubacterium hallii* group and *Subdoligranulum*), *Erysipelotrichaceae* UCG-003 as the most important genus feature in RF had the strongest correlation with changes in body fat mass (**Figure S8**).

<u>Additional file 1: Figure S8</u>

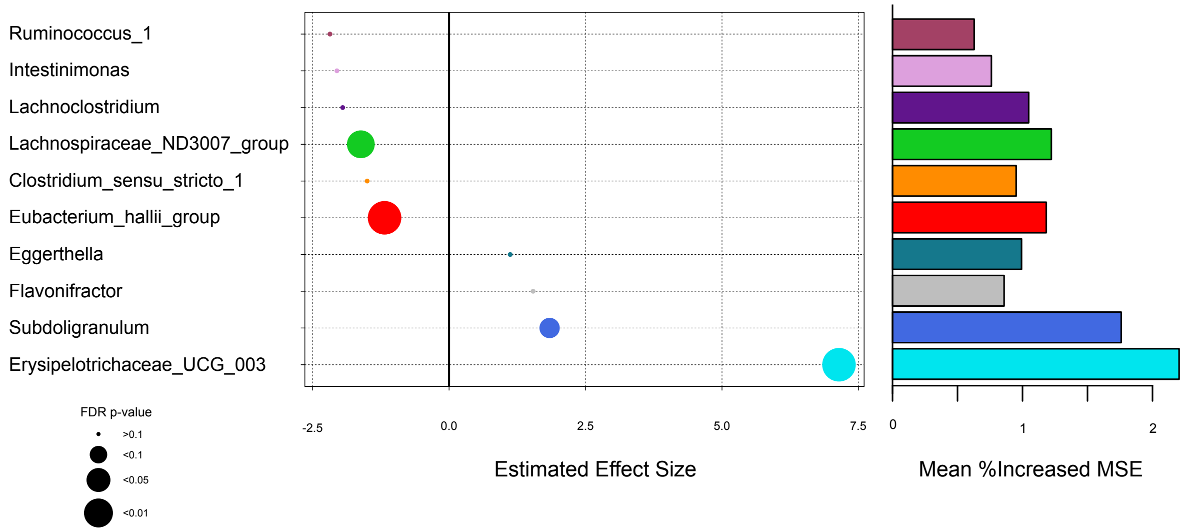

Figure S8. Correlation between change in body fat mass and 10 bacterial taxa selected by random forests (RF) as being most highly predictive of body fat change. The correlation was estimated by linear mixed-effects models adjusting for demographic variables. The bar graph on the right denotes the importance, based on the percentage increase in mean-squared error (MSE), of the 10 genera to the accuracy of the RF model.

References

1. Yin Y, Fan B, Liu W, Ren R, Chen H, Bai S, Zhu L, Sun G, Yang Y, Wang X: **Investigation into the stability and culturability of Chinese enterotypes.** *Sci Rep* 2017, **7:**7947.
2. Caporaso JG, Lauber CL, Walters WA, Berg-Lyons D, Huntley J, Fierer N, Owens SM, Betley J, Fraser L, Bauer M, et al: **Ultra-high-throughput microbial community analysis on the Illumina HiSeq and MiSeq platforms.** *Isme j* 2012, **6:**1621-1624.
3. Ramiro-Garcia J, Hermes GDA, Giatsis C, Sipkema D, Zoetendal EG, Schaap PJ, Smidt H: **NG-Tax, a highly accurate and validated pipeline for analysis of 16S rRNA amplicons from complex biomes.** *F1000Res* 2016, **5:**1791.
4. Vázquez-Baeza Y, Pirrung M, Gonzalez A, Knight R: **EMPeror: a tool for visualizing high-throughput microbial community data.** *Gigascience* 2013, **2:**16.
5. Stanislawski MA, Dabelea D, Wagner BD, Iszatt N, Dahl C, Sontag MK, Knight R, Lozupone CA, Eggesbø M: **Gut Microbiota in the First 2 Years of Life and the Association with Body Mass Index at Age 12 in a Norwegian Birth Cohort.** *mBio* 2018, **9**.
