# Supplementary material for: Gut microbiota predicts body fat change following a low-energy diet: a PREVIEW intervention study
Source: Genome Med. 2022 May 23;14:54. doi: 10.1186/s13073-022-01053-7 (PMC9125896; doi:10.1186/s13073-022-01053-7)
Supplement: Supplementary file 3 — Additional file 3. PREVIEW study protocol. [file 13073_2022_1053_MOESM3_ESM.pdf]

PREVIEW – PREVention of diabetes through lifestyle Intervention and population studies in Europe and around the World

Project no.: 312057

Seventh Framework Programme

Instrument: Collaborative Project

FP7-KBBE-2012

Study protocol and flow charts

(WP1)

TABLE OF CONTENTS

1. SIGNATURES AND AGREEMENT WITH PROTOCOL 8
2. SUMMARY 10
3. BACKGROUND AND GENERAL AIM 12
4. OBJECTIVES, HYPOTHESES AND END-POINTS........ 14
4.1 Main objectives 14
4.2 Endpoints:........ 15
4.2.1 Primary endpoint: 15
4.2.2 Secondary endpoints: 15
5. DESIGN 17
5.1 General design and time schedule 17
5.2 Visits 18
5.2.1 Screening and randomisation (visit 1) 18
5.2.2 Baseline – CID1 (visit 2)........ 19
5.2.3 Weight-reduction (visit 2, 3, 4, 5 and 6) 19
5.2.4 Dietary intervention (Adults: visit 7 – 19; Children: visit 7-17)........ 19
5.2.5 Physical activity interventions 22
5.3 Blinding........ 24
6. PARTICIPANTS........ 24
6.1 Number of participants overall and in different sites........ 24
6.2 Inclusion Criteria........ 25
6.3 Exclusion criteria 25
6.4 Recruitment and Informed Consent procedures 27
6.4.1 Re-screening........ 28
6.5 Procedures for exclusion/withdrawal from the study 30
7. METHODS 30
7.1 Time-window and general principles for data collection........ 30
7.2 Collection of basic and contact information 31
7.3 Demographic data collection 31
7.4 Anthropometric data and body composition........ 31
7.4.1 Body weight and height 31
7.4.2 Waist, hip, and thigh circumference........ 32
7.4.3 Body composition and liver fat content........ 32

7.5 Blood pressure and heart rate ... 33
7.6 Biological Samples ... 33
7.6.1 OGTT and blood sample ... 33
7.6.2 Sampling and analyses of DNA and RNA ... 34
7.6.3 Amount of blood sampled ... 35
7.6.4 Urine samples ... 35
7.6.5 Faecal samples ... 36
7.6.6 Storage and analysis of biological samples ... 36
7.7 Assessment of food consumption, nutrient intakes and GI ... 36
7.8 Assessment of dietary restraint ... 37
7.9 Assessment of appetite ... 37
7.10 Assessment of physical activity ... 37
7.10.1 Baecke questionnaire and physical activity log ... 37
7.10.2 Accelerometry ... 38
7.11 Assessment of physical fitness ... 38
7.12 Assessment of Adverse events and Concomitant Medicine ... 38
7.13 Questionnaires related to moderators, mediators, behaviour and social environment 39
7.14 Assessment of sleep ... 41
7.15 Assessment of food reward ... 42
7.16 Assessment of stress, mood and anxiety ... 42
7.17 Cost-effectiveness ... 43
8. ADVERSE EVENTS ... 43
8.1 Definition of Adverse Events ... 43
8.2 Methods for Eliciting, Recording and Follow-up of Adverse Events ... 43
8.3 Assessment of Intensity ... 43
8.4 Assessment of Causality ... 44
8.5 Definition of Serious Adverse Events ... 44
8.6 Reporting of Serious Adverse Events ... 45
8.7 Follow-up of Adverse Events and Serious Adverse Events ... 45
8.8 Procedures in Case of Medical Emergency ... 45
9. DATA MANAGEMENT AND QUALITY ... 46
9.1 Data quality ... 46
9.2 Confidentiality ... 46
9.3 Data Quality Assurance ... 46
9.4 Statistical considerations ... 47

9.4.1 Sample size calculation ... 47
9.4.2 Handling of missing values ... 48
9.4.3 Power calculations on non-primary outcomes: ... 49
10. ETHICAL ASPECTS ... 50
10.1 General ... 50
10.2 Participant information and Informed Consent ... 51
10.3 Medical safety ... 52
10.4 Children's participation ... 52
10.5 Privacy of participants ... 53
10.6 Participant's right to their own results ... 53
10.7 Insurance ... 54
10.8 Reimbursement ... 54
11. TRANSLATIONS ... 54
12. SCIENTIFIC PUBLICATIONS ... 54
13. FINANCIAL CONDITIONS ... 55
14. TIME-SCHEDULE ... 55
15. RESEARCH TEAM AND RESPONSIBILITIES ... 56
16. LIST OF INSTRUCTION PAPERS, QUESTIONNAIRES AND SOP'S ... 59
16.1 Instruction papers ... 59
16.2 Questionnaires ... 59
16.2.1 Adults ... 59
16.2.2 Children and adolescents ... 60
16.3 SOP's ... 61
16.4 Cooking books ... 62
Cooking book: HP diet (Ins-8a) ... 62
Cooking book: MP diet (Ins-8b) ... 62
17. REFERENCES ... 62
APPENDICES ... 64
Appendix 1: Study Flow Chart - Adults. ... 64
Appendix 2: Study Flow Chart - Children and Adolescents ... 66

LIST OF FIGURES

Figure 1: The screening process 29

Figure 2: Participant flow during the study (presented for the 4 arms). 48

LIST OF TABLES

Table 1: The four study groups 17

Table 2: Principles of the dietary interventions 20

Table 3: Principles of the physical activity interventions 23

Table 4: Number of participants to start the 8 week weight reduction period 24

Table 5: PREVIEW intervention – Inclusion criteria 25

Table 6: Questionnaires on moderators, mediators, behaviour and social environment - adults 39

Table 7 Questionnaires on moderators, mediators, behaviour and social environment - children 41

Table 8: Time schedule for PREVIEW study 55

Table 9 – PREVIEW research team 56

LIST OF ABBREVIATIONS AND DEFINITIONS

Abbreviation	Full name
**AE**	Adverse event
**ALT**	Alanine Transaminase
**ANCOVA**	Analysis of the Covariance
**ASQ**	Adolescents Stress Questionnaire
**AST**	Aspartate Transaminase
**AUC**	Area under the curve
**BMI**	Body mass index
**BP**	blood pressure
**CDC**	Centres for Disease Control
**CID**	Clinical investigation day
**CRP**	C-reactive protein
**DNA**	Deoxyribonucleic acid
**DPS**	Diabetes prevention study
**DXA**	Dual X-ray absorptiometry
**eCRF**	Electronic case report form
**ECG**	Electrocardiography
**EDTA**	Ethylenediaminetetraacetic acid
**EHES**	European Health Examination Survey
**EOT**	End of trial
**ESS**	Epworth Sleepiness Scale
**FOV**	Field of view
**FPG**	Fasting plasma glucose
**GCP**	Good clinical practice
**GI**	Glycaemic index
**GP**	General practitioner
**HbA1c**	Glycated haemoglobin
**HDL**	High-density lipoprotein
**HEL**	University of Helsinki
**HI**	High-intensity (exercise)
**HIV**	Human immunodeficiency virus
**H-MRS**	Hydrogen Magnetic Resonance Spectroscopy
**HOMA-IR**	Insulin resistance analysed by the homeostatic model
**HP**	High-protein (diet)
**HR**	Heart rate
**HRR**	Heart rate reserve
**ICF**	Informed Consent Form
**ICH**	International Conference on Harmonisation
**ID**	Identifier
**IDF**	International Diabetes Federation
**IEC**	Independent Ethics Committee
**IFG**	Impaired fasting glucose
**IGT**	Impaired glucose tolerance
**IPAI**	Influence on Physical activity Instrument
**INS**	Instruction
**ITT**	Intention to Treat
**LDL**	Low-density lipoprotein
**LCD**	Low-calorie diet

Abbreviation	Full name
**LGI**	Low Glycaemic Index
**MET**	Metabolic equivalent of task
**MGI**	Moderate Glycaemic Index
**MI**	Moderate-intensity (exercise)
**MP**	Moderate-protein (diet)
**MRI**	Magnetic resonance imaging
**MU**	Medical University of Sofia
**OGTT**	Oral glucose tolerance test
**PI**	Principal Investigator
**POMS**	Profile of Mood Scale
**PSQI**	Pittsburgh Sleep Quality Index
**PSS**	Perceived Stress Scale
**RCT**	Randomized, controlled trial
**RE-AIM**	Reach Efficacy/Effectiveness, Adoption, Implementation and Maintenance
**REM**	Rapid-Eye-Movement
**RNA**	Ribonucleic acid
**RQ**	Respiratory quotient
**SAE**	Serious adverse event
**SCFA**	Short-chain fatty acids
**SCT**	Social Cognition Theory
**SDT**	Self-determination theory
**SOP**	Standard operation procedure
**SWS**	Slow Wave Sleep
**T2D**	Type-2 diabetes
**TFEQ**	Three-factor Eating Questionnaire
**THL**	The National Institute for Health and Welfare (Finland)
**TRSQ**	Treatment self-regulation questionnaire for diet & exercise
**TTM**	Trans-theoretical model
**UCPH**	University of Copenhagen
**UM**	University of Maastricht
**UNAV**	University of Navarra
**UNOTT**	University of Nottingham
**UNSYD**	University of Sydney
**UOA**	University of Auckland
**USTUTT**	University of Stuttgart
**VAS**	Visual Analogue Scale
**VO_2max**	Maximal oxygen uptake capacity
**WAI**	Work Ability Index
**WHO**	World Health Organisation
**WHOQOL**	World Health Organisation Quality of Life
**WMA**	World Medical Association
**WP**	Work package

1. SIGNATURES AND AGREEMENT WITH PROTOCOL

We, the undersigned academic partners, acknowledge that we have read this protocol. We agree to conduct this study in accordance with the study protocol, the current version of the Declaration of Helsinki (59. WMA-General Assembly, Seoul, Korea, October 2008), and the ICH-GCP, International Conference on Harmonization E6 Good Clinical Practice, to the extent that this is possible and relevant. In addition, all national laws and regulations of the local ethical committee regarding human research will be strictly enforced**.**

Mikael Fogelholm, University of Helsinki (HEL), Finland, protocol responsible (WP1 leader), site-PI

Thomas Meinert Larsen, University of Copenhagen (UCPH), Denmark, site-PI

Margriet Westerterp-Plantenga, University of Maastricht (UM), The Netherlands, site-PI

Ian Macdonald, University of Nottingham (UNOTT), United Kingdom, site-PI

J Alfredo Martínez, University of Navarra (UNAV), Spain, site-PI

Svetoslav Handjiev, Medical University of Sofia (MU), Bulgaria, site-PI

Jennie Brand-Miller, University of Sydney (UNSYD), Australia, site-PI

Sally Poppitt, University of Auckland (UOA), New Zealand, site-PI

Wolfgang Schlicht, University of Stuttgart (USTUTT), Germany

Gareth Stratton, Swansea University (SU), United Kingdom

Jouko Sundvall, National Institute for Health and Welfare (THL), Finland

Anne Raben, University of Copenhagen (UCPH), Denmark
Sponsor and Coordinator of the PREVIEW project

2. SUMMARY

Type-2 diabetes mellitus (T2D) is one of the fastest growing chronic diseases worldwide. This trend is mainly driven by a global increase in the prevalence of obesity. The PREVIEW-project has been initiated to find out the most effective lifestyle-components (diet and physical activity) in the prevention of T2D. The project was initiated by Prof Jennie Brand-Miller (Sydney Australia) and Prof. Anne Raben (Copenhagen, Denmark), and the entire project consists of 6 work packages:

WP1: Multicentre intervention: Randomized, controlled, multicentre trial (RCT)
WP2: Population studies
WP3: The role of sleep and stress in interaction with the role of diet and physical activity
WP4: Other lifestyle variables: Behavioural, sociological, environmental, cultural, socio-ecological, and socioeconomic components
WP5: Dissemination and exploitation
WP6: Management.

This protocol is part of WP1, but involves also WP3 and WP4.

The aim of the PREVIEW intervention is to determine the preventative impact of a high-protein and low-GI diet in combination with moderate or high-intensity physical activity on the incidence of T2D in predisposed, pre-diabetic children, young, and older adults (both gender). Therefore, the project addresses prevention in individuals with high risk for T2D. This will be done by conducting two randomized, controlled, multicentre trials (RCT) among participants at risk of developing diabetes (impaired glucose tolerance (IGT), i.e., overweight or obesity and increased diabetes risk factors). The larger trial involves adult participants, while the smaller involves children and adolescents. The adult intervention will be performed in 8 intervention sites in 6 EU countries: Bulgaria, Denmark, Finland, Spain, the Netherlands, United Kingdom, and in Australia and New Zealand, respectively. The children intervention will be performed in 3 intervention sites in the Netherlands, Spain and the United Kingdom.

A total of up to 2,500 overweight or obese adult participants (25 - 70 y) and 150 children and adolescents (10-18 y) will be recruited. All adult participants are first treated by a low-calorie diet (LCD) for 8 weeks, with an aim to reach ≥ 8% weight reduction. Children and adolescents are treated separately with a conventional weight-reduction diet, without a specific aim for absolute weight loss.

The adult participants are randomized into two different diet interventions and two exercise interventions for a total of 148 weeks (3 years in total). This period aims at preventing T2D by weight-maintenance (prevention of relapse in reduced body weight) and by independent metabolic effects of diet and physical activity. The children and adolescents are in principle also randomised into the same 2 diet and 2 exercise arms for 96 weeks (2 years in total). However, to increase adherence to the study, they are allowed to use exercise prescriptions from both arms. Post hoc analyses will reveal which kind of exercise they actually performed. The aim is to increase insulin sensitivity and prevent increase in BMI by independent metabolic effects of diet and physical activity.

The two diet interventions are: MP = moderate-protein diet: protein 15% of total energy intake (E%), carbohydrates 55 E%, dietary glycaemic index (GI) >56; HP = high-protein diet: protein 25 E%, carbohydrates 45 E%, GI <50. Both diets are composed by using healthy food items. The two exercise interventions are: MI = moderate-intensity: 60—75% of maximal heart rate (HRmax), e.g., brisk walking for adults; HI = high-intensity: 76—90% HRmax, e.g., running for adults. Since the participants are randomized into one diet as well as one exercise group, the total number of combinations (intervention groups) is four (MP-MI, MP-HI, HP-MI and HP-HI). The randomization for the adults is stratified by sex and age group, and by sex in children.

The participants are weighed and supervised in groups during the weight-reduction (4 times) and throughout the weight-maintenance period. Meeting frequency is reduced towards the end of the study: there are eight meetings during the first 10 months of weight-maintenance intervention (study months 2—12), three during the second study year and 2 during the last study year. The supervision of diet and physical activity is based on psychological theories on behaviour change. Children follow a similar, but slightly looser time schedule and counselling is done individually.

The main assessment points (clinical investigation days, CID) are at weeks 0 (baseline, start of weight-reduction), 8 (end of weight-reduction/start of randomized intervention), 26 (6 months from baseline), 52 (12 months from baseline), 78 (18 months from baseline), 104 (24 months from baseline) and 156 (36 months from baseline / End of Trial, EOT). For the children and adolescents the last assessment point is at week 104 (24 months from baseline / End of Trial, EOT).

The primary endpoint of the study for adults is the incidence of type-2 diabetes during 3 years (156 weeks) according to diet (high protein/low-GI versus moderate protein/medium-GI, adjusted for physical activity). The primary endpoint for children and adolescents is the reduction in Tanner-stage corrected HOMA-value during 2 years (104 weeks) according to diet (high protein/low-GI versus moderate protein/medium-GI, adjusted for physical activity).

Type-2 diabetes can be verified by one of the following ways:

1) Fasting plasma glucose ≥7.0 mmol/L (126 mg/dL) or 2 h glucose in OGTT ≥11.1 mmol/L (200 mg/dL), assessed by Hemocue at the CID's of PREVIEW (two tests on separate occasions 2-4 weeks apart are required), or

2) Type-2 diabetes diagnosed by a medical doctor between the CID's of PREVIEW by using random plasma glucose ≥11.1 mmol/L in the presence of symptoms of diabetes, OGTT or HbA1c, with or without concomitant medical treatment.

The main secondary endpoint for adults is the incidence of type-2 diabetes during 3 years (156 weeks) according to physical activity (high-intensity physical activity versus moderate-intensity physical activity, adjusted for diet).

Other secondary or exploratory endpoints are changes in HbA1c (a measure of average blood glucose levels), body weight, waist circumference and body fat mass (kg, proportion of body weight), proportion of subjects maintaining at least 0, 5 and 10% weight loss (relative to baseline body weight), insulin sensitivity (Matsuda Index based on OGTT), beta-cell disposition index, risk factors for cardiovascular disease

(blood pressure, lipids, C-reactive protein and liver enzymes), changes in perceived quality of life and workability, habitual well-being and chronic stress, sleep duration and quality (accelerometer and questionnaire), appetite sensations, and habitual (long-term) physical activity.

In addition to the above listed outcome variables, we collect data in all participants on dietary intake (food diaries, urinary nitrogen excretion), actual physical activity (training log, accelerometer), genetic profile (in centres where this is possible and allowed according to local law), moderators and mediators of behaviour change, cost-effectiveness, public health impact - (Reach Efficacy/Effectiveness, Adoption, Implementation and Maintenance - (RE-AIM). Adverse events and use of concomitant medication (with focus on diabetes and its related complications) will also be registered. For kidney safety measures, we also analyse serum creatinine and urinary albumin in a sub-group of 100 elderly subjects (+55 y).

In selected sub-groups we collect data on metabolomics profile (urine), colon cancer risk markers and gut microbiota (faecal samples), liver fat content, kidney safety measures, maximal oxygen uptake capacity (VO2 max), sleep architecture, and food reward.

3. BACKGROUND AND GENERAL AIM

Type-2 diabetes is one of the fastest growing chronic diseases worldwide. This is primarily due to the increasing prevalence of obesity, caused by a sedentary and inactive lifestyle and general food availability. It is estimated that in year 2000 there were approximately 150 million individuals with type-2 diabetes and that this number is likely to double by 2025 (1). The relative risk of getting type-2 diabetes rises exponentially with increasing body mass index (BMI) and at a BMI above 23 kg/m^2 the risk of getting type-2 diabetes has already doubled (2). Over the past 20 years the US has experienced a surge in overweight and obesity, and the prevalence of type-2 diabetes has increased in parallel with these conditions. People with diabetes have a 2-4 times higher risk of dying from heart disease compared to those without diabetes (3) and the health and economic costs related to the increased prevalence of diabetes in both young adults and the ageing population are huge (4).

The global increase in the prevalence of obesity is most probably driven by a simultaneous increase in global food abundance and food of reduced nutritional quality, together with increased sedentariness and decreased physical activity during both work and possibly leisure time (5). Recent studies have also indicated that a deviation from the normal sleeping pattern of 7-8 hours' sleep per night, particularly short duration of sleep, increases appetite and promotes obesity and its related diseases (e.g. type-2 diabetes and cardiovascular disease)(6,7).

There are two ways to prevent type-2 diabetes:

1) By preventing weight gain and/or by lifestyle modification in the general population (population-approach);

2) By weight loss and maintenance, together with lifestyle modification, in at-risk individuals (pre-diabetics).

The main drivers in both situations are changes in dietary and physical activity patterns (8, 9), but also sleep and stress may be important. Unfortunately, despite convincing evidence from clinical trials that

type-2 diabetes can be prevented or delayed through intensive lifestyle interventions resulting in weight loss (8,9), the reality is that weight regain and incremental weight 'creep' are very common. It is possible that this may jeopardise diabetes prevention and the effect of a longer period of weight maintenance on prevention remains to be seen.

The recent EU 6th Framework study "DiOGenes" (Diet, Obesity and Genes) (Grant Agreement no. 513946) (10) identified two dietary factors associated with shorter-term prevention of weight regain after prior weight loss: higher protein intake and lower glycaemic index (GI). The findings showed that overweight and obese participants assigned to the combination of modestly higher protein and lower GI ad libitum had significantly better completion rates and weight maintenance after 6 months as compared with the official dietary guidelines. Indeed, those consuming the high protein-low GI combination diet continued to lose weight during the weight-maintenance phase and were twice as likely to have maintained a 5% weight loss compared to the other groups.

The interesting point from DiOGenes was that current nutrition recommendations (e.g. Nordic Nutrition Recommendations) typically promote an intake of 50—60 E% carbohydrate and no more than 20 E% protein. This was also the target in the Finnish Diabetes Prevention Study, DPS (8), which is considered one of the landmark studies in this area. Both the national guidelines for dietary treatment and prevention of type-2 diabetes in the US (11) and the UK (12) point out that an ideal macronutrient composition for preventing and treating type-2 diabetes is still not known. It is possible that the range of carbohydrates and proteins in the diet may be wider than commonly believed.

In the present study, we aim at comparing the preventive effects of two diets. One of the diets has lower carbohydrate and higher protein intakes than is normally achieved, i.e. the target is similar to the high-protein, low-GI diet in the DiOGenes study. The other test diet, on the other hand, has a target carbohydrate intake in the upper achievable end and with less emphasis on glycaemic index, hence resembling the dietary aims of the Finnish Diabetes Prevention Study (DPS), which has already been documented to prevent type-2 diabetes (8).

We hypothesize that the high protein-low GI diet may even be superior to moderate protein-moderate GI diet for both diabetes prevention and the reduction of its complications.

Although the positive role of physical activity in prevention of type-2 diabetes has been well established (13—15), the role of exercise intensity is unclear (16). Moreover, the data on increased physical activity combined with lowered carbohydrate intake is very limited (17,18).

A further issue is potential interaction between age and prevention of type-2 diabetes. It is not known whether the standard strategies are equally effective in young, adult and ageing individuals. Besides age, social-ecological variables, such as social-cognitive determinants of behavioural change, habits, or social and environmental factors influence the success of weight-loss maintenance. In addition there seems to be an interaction between obesity, diabetes and sleeping pattern. This interaction is independent of physical activity levels (7). So, there are other factors besides diet that may be important to help diabetes prevention.

The primary goal of the PREVIEW intervention study is to identify the most efficient lifestyle pattern for the prevention of type-2 diabetes in a population of pre-diabetic overweight or obese adults (i.e. those

at high risk of diabetes) and for the improvement of insulin sensitivity in a population of overweight or obese children and adolescents with reduced insulin sensitivity . This will be done by conducting 2 multi-centre, multinational, clinical randomized intervention trials. One larger of 3 years duration in a total of up to 2,500 pre-diabetic adult participants, and one smaller of 2 years duration including up to 150 children and adolescents.

4. OBJECTIVES, HYPOTHESES AND END-POINTS

4.1 Main objectives

Our main objectives in the PREVIEW intervention studies are:

1) To determine the preventive effect of a high protein-low GI **diet** in combination with either moderate or high-intensity **physical activity** on the incidence of **type-2 diabetes** in predisposed, pre-diabetic adults (25 - 70 y), and on insulin sensitivity in children and adolescents (10-18 y) with reduced insulin sensitivity. This will be done by conducting two **randomized, controlled, multicentre trials (RCT).** One large trial will be conducted with up to 2,500 adults at high risk of developing diabetes (i.e. overweight or obesity with BMI $\geq$ 25 kg/m^2 and increased diabetes risk factors). This trial will be performed worldwide including 6 EU nations: Bulgaria, Denmark, Finland, Spain, the Netherlands, the United Kingdom, as well as Australia and New Zealand (WP1). The other will be conducted in up to 150 children and adolescents in 3 EU intervention sites: the Netherlands, Spain and the United Kingdom.

   - Our hypothesis is that a high-protein, low-GI diet (as used in the DioGenes study; protein intake higher than in the present recommendations for preventing and treating T2D) will be superior in preventing type-2 diabetes and improving insulin sensitivity, compared with a moderate protein, moderate GI diet (as used in the DPS study; within the present recommendations for preventing and treating T2D), and that high-intensity physical activity will be superior compared to moderate-intensity physical activity.

2) To evaluate the role of **sleeping pattern and chronic stress** on the development of diabetes in such pre-diabetic subjects - and their interaction with **diet and physical activity** (WP3).

   - Our hypothesis is that a high-protein, low-GI diet in interaction with high-intensity physical activity may improve the sleeping pattern and reduce the level of chronic stress during the weight-maintenance phase - ultimately reducing the risk for T2D in pre-diabetic adult subjects and improve insulin sensitivity in the children and adolescents.

3) To evaluate the moderating and/or mediating influence of social-ecological variables such as social-cognitive determinants of **behavioural change and habitual behaviour, social environmental influences, cultural habits, socio-ecological and socio-economic components** for individuals at risk of developing diabetes (WP4).

   - Our hypothesis is that the recommended behaviour change and its maintenance are influenced by these variables. Thus, low self-efficacy and lack of social support could both by themselves and in combination cause a relapse back to an earlier, unhealthy behaviour.

4.2 Endpoints:

4.2.1 Primary endpoint:

For adults:

Incidence of type-2 diabetes, in high protein versus medium protein diet, measured during 3 years after baseline and based on WHO/IDF criteria:

- Fasting plasma glucose ≥7.0 mmol/L (126 mg/dL) or 2 h glucose in OGTT ≥11.1 mmol/L (200 mg/dL), assessed by Hemocue at the CID's of PREVIEW (two tests on separate occasions 2-4 weeks apart are required), or
- Type-2 diabetes diagnosed by a medical doctor between the CID's of PREVIEW, by using random plasma glucose ≥11.1 mmol/L in the presence of symptoms of diabetes, OGTT or HbA1c, with or without concomitant medical treatment.

Asymptomatic individuals with a single abnormal value will have the test repeated to confirm the diagnosis.

For children and adolescents:

Change in insulin resistance at 2 years after randomization to high protein versus medium protein diet, measured by insulin resistance analysed by the homeostatic model (HOMA-IR).

4.2.2 Secondary endpoints:

(tested against the possible combinations of diet and physical activity if not stated otherwise)

- The effects of high-intensity vs. moderate-intensity physical activity on incidence of type-2 diabetes in adults, based on WHO/IDF criteria (adjusted for diet);
- Change in HbA1c, a measure of average blood glucose levels;
- Change in body weight, BMI, waist, hip and thigh circumference;
- Change in body fat mass (kg, proportion of body weight); FMI: Fat mass index
- Proportion of adult subjects maintaining at least 0, 5 or 10% weight loss (relative to initial body weight);
- Insulin sensitivity (e.g. Matsuda Index based on the OGTT, glucose area under the curve (AUC) during OGTT, beta-cell disposition index) (OGTT only adults);
- The effects of stature (height; proportion leg-length/height) in adults and changes in stature in children and adolescents, on the changes in relationship between reduction in body weight, body fat and insulin sensitivity:
- Risk factors for cardiovascular disease, with at least the following measures: blood pressure, lipids (triglycerides, total, low-density lipoprotein (LDL) and high-density lipoprotein (HDL) cholesterol), C-reactive protein, and liver enzymes;
- Changes in perceived quality of life and workability, habitual well-being, sleep and chronic stress, subjective appetite sensations, and habitual physical activity.

- In sub-groups:
    - Liver fat content by MRI and H-MRS (adults) (sub-group A)
    - Colon cancer risk markers and microbiota (faecal samples) (adults) (Sub-group B)
    - VO_2 max (adults) (sub-group C)
    - Metabolomics profile (urine samples) (adults) (sub-group D)
    - Food reward (adults) (sub-group E)
    - Sleep architecture (children and adolescents) (sub-group F)
    - Kidney safety measures (100 subjects +55 y, sub-group G).

Finally, adverse events and use of concomitant medication will be registered.

Primary hypotheses: The following primary hypotheses can be applied:

Adults

H_0:

Incidence of type-2 diabetes between baseline and week 156 of the study is equal in the intervention groups (high protein vs. medium protein).

vs.

H_1: Incidence of type-2 diabetes between baseline and week 156 of the study is lower in the high protein vs medium protein intervention group.

Children and adolescents:

H_0:

Change in insulin sensitivity between baseline and week 104 of the study is equal in the intervention groups (high protein vs. medium protein).

vs.

H_1: Change in insulin sensitivity between baseline and week 104 of the study is more positive for the high protein than for the medium protein intervention group.

5. DESIGN

5.1 General design and time schedule

The study in adults will be carried out as a 3 year (156 weeks) multi-national, randomized, clinical intervention trial in 8 sites in different countries. The study consists of two distinct parts, namely an 8-wk weight-reduction period (same for all adult participants regardless of intervention group assignment), followed by a 148-wk randomized weight-maintenance intervention. The first part is preceded by pre-screening, screening and randomization of eligible participants. Subjects are randomized into four groups as given in Table 1.

Children and adolescents will follow a 2-year (104 weeks) randomized, clinical intervention trial in 3 sites in different countries. They will be randomized after 8 weeks weight stability (and BMI reduction) for a 96 weeks BMI maintenance intervention.

A total of up to 2,500 eligible adult participants (25-70 y) and up to 150 children and adolescents (10 - 18 y). The plan is to recruit an equal number of participants (n = 310-15) from each of the 8 study sites. Adults will be recruited from all 8 sites, whilst children and adolescents will be recruited from 3 sites (UM, UNAV, and SU).

Adult participants with successful weight reduction (at least 8% of initial body weight) will continue into the weight-maintenance phase of the study.

Table 1: The four study groups

	**High protein (25 E%)<br>Moderate carbohydrate (45 E%)<br>Low GI (<50) diet**	**Moderate protein (15 E%)<br>High carbohydrate (55 E%)<br>Medium GI (>56) diet**
**High-intensity physical activity (> 6 MET)**	Group 1: HP-HI	Group 3: MP-HI
**Moderate-intensity physical activity (3-6 MET)**	Group 2: HP-MI	Group 4: MP-MI

The abbreviations refer to the dietary regimen (HP/MP) and to the intensity of physical activity (HI/MI).
MET = Metabolic equivalent of task (energy expenditure compared to resting energy expenditure). Note that the MET's may vary on individual basis because of variation in fitness (intensity is always relative to a participant's maximal fitness).

The intervention is carried out by using a "fading visit" approach, that is, the frequency of meetings and supervision gradually declines towards the end of the study (see flow-charts, Appendix 1 and 2).

The main assessment points (clinical investigation days, CID) are at the following weeks:

- Week 0 (CID1, baseline, start of weight-reduction with LCD)
- Week 8 (CID2, end of weight-reduction/start of randomized intervention)
- Week 26 (CID3, 6 months from baseline and 4 months after randomization)
- Week 52 (CID4, 12 months from baseline and 10 months after randomization)

- Week 78 (CID5, 18 months from baseline and 16 months after randomisation; lightened protocol)
- Week 104 (CID6, 24 months from baseline and 22 months after randomization). For children and adolescents: End of Trial (EOT).
- Week 156 (CID7, 36 months from baseline and 34 months after randomization / End of Trial, EOT). Adults only.

5.2 Visits

Visits and measurements are given in the flow-charts (Appendix 1 for adults and Appendix 2 for children/adolescents).

Individuals considered eligible based on pre-screening phone-interview (see section 6.4) performed in accordance to inclusion and exclusion criteria are sent the written patient information and invited for an information meeting at the site. At the information meeting, the study is explained carefully and questions are answered. Individuals, who are still interested in participating, can sign the informed consent form at the meeting. Hereafter, the screening visit is scheduled. Those who need more time to consider, are allowed to take the consent form home and return it later, if they decide to participate.

The informed consent form must always be signed and dated both by the participant (or its parent) the investigator, or by the project worker - who has been delegated by the Investigator in accordance to the delegation log and qualified to perform the oral information - before any procedures related to the protocol can be performed.

5.2.1 Screening and randomisation (visit 1)

After the signed and dated informed consent form is obtained. Participants are considered eligible based on in- and exclusion criteria, and weight, height and blood pressure are taken and the participants are given a simplified OGTT (fasting 0 and post-load 120 min measurements). A blood specimen is also taken simultaneously. See further information on recruitment, section 6.4. Safety check and exclusion criteria necessitate a blood specimen in all adults as well as an electrocardiography (ECG) in the oldest age group (55-70 y).

Relevant medical history will be recorded. The participant is then randomized to one of the four intervention groups. Randomization of all adult study subjects is done by stratifying the participants according to age-group and sex (men, women). The randomization is done in blocks in order to ensure an equal number of participants in each group. The randomization is not disclosed to the participants at this time point. For the children, the stratification is done according to sex.

- ➔ **SOP-5a: Pre-screening and screening, adults**
- ➔ **SOP-5b: Pre-screening and screening, children**
- ➔ **SOP-5c: OGTT during Screening**
- ➔ **SOP-5d: Guidance for Type-2 diabetes diagnose in PREVIEW study**
- ➔ **SOP-24: Concomitant Medication**
- ➔ **SOP-19: Randomization**

5.2.2 Baseline – CID1 (visit 2)

Before entering the 8 weeks' weight-reduction phase (for children 8 weeks' weight maintenance phase), the participants visit the site for the baseline measurements. The procedure includes fasting blood sample, a 2-h OGTT (0, 30, 60, 90 and 120 min measurements) (adults only), anthropometric measurements and body composition, 4-day food records, 7-d accelerometer (sleep and physical activity), questionnaires related to e.g. physical activity, moderator and mediator of behaviour change, perceived stress, dietary restraint and sleep. A sub-sample in adults will provide baseline data for metabolomics analyses (urine collection), physical fitness (VO_2max), colon cancer risk markers and microbiota (faecal sample), liver fat content (MRI), and food reward. In a sub-sample of children and adolescents sleep architecture will be examined.

5.2.3 Weight-reduction (visit 2, 3, 4, 5 and 6)

The energy restricted LCD diet (for adults only) consists of 800-1000 kcal/d and the target macronutrient composition of the diet will be 15-20% of total energy from fat, 35-40% from protein and 45-50% from carbohydrate. During this period, subjects will attend five group meetings at the site-centre where their body weight, adverse events (AE) and concomitant medication will be registered and dietary and behavioural instructions will be given. No specific instructions on physical activity are given during the weight-reduction phase. All adult participants should follow their LCD until they are measured at the CID2 visit (wk 8). The Cambridge Weight Plan is used for all centres.

Children and adolescents, regardless of whether they have reached their full height or not, are treated separately with a conventional weight-reduction diet, without a specific aim for absolute weight loss. The target for children's energy intake will be based on the Schofield equation adjusted for activity with no correction for growth. A healthy personal dietary regime will be designed by a dietician (without energy-dense, nutrient-poor foods; with fibre, fruit, and vegetable intake; timely, regular meals; and avoidance of "grazing" during the day).

The weight-reduction period for adults is reinforced during group meetings led by a dietician/weight loss counsellor. The meetings are held on weeks 0, 2, 4, and 6. There is also a meeting on week 8, following the CID2, at which participants will be introduced to the intervention to which they have been randomised. Individual meetings are performed for children and adolescents at weeks 0 and 4.

LCD details are included in the Standard Operation Procedure (SOP) nr. 1.

- ➔ **SOP-1: LCD**
- ➔ **SOP-22: Children diet during "LCD"**
- ➔ **Ins-10: Instructions to participants regarding the Low Calorie Diet (LCD) (adults)**
- ➔ **Ins-11: Instructions to participants - Children diet during LCD**

5.2.4 Dietary intervention (Adults: visit 7 – 19; Children: visit 7-17)

Adult participants with at least an 8% weight loss at the CID2 visit are allowed to start the randomized intervention phase. After the screening visit, participants have already been randomized to one of the dietary interventions, HP = High protein (25 E%), moderate carbohydrate (45 E%), starchy food items

with low GI, or MP = Moderate protein (15 E%), high carbohydrate (55 E%), starchy food items with moderate GI.

The diets are to be consumed *ad libitum* with respect to energy, i.e. the participants are not asked to count the energy content, and they are not provided with an individual target for energy intake. However, they will be instructed about the importance of controlling portion size of particular food types in order to achieve the macronutrient / GI prescription, and in self-monitoring and adjustment of portion sizes in general, in order to maintain weight loss. They will also be advised to have a regular meal pattern. They will be repeatedly reminded, at the group meetings and occasionally by text message or e-mail, about the importance of maintaining weight at the level achieved after the weight-reduction phase. Additional weight reduction is allowed, but without any other means than the study diet and physical activity regimens (e.g. LCD's using commercial products and/or weight-reducing drugs are not allowed in the weight-maintenance period, except for the 2 weeks immediately after the LCD period).

The idea of the food-based dietary guidelines is that both diets are planned to be healthy and supportive for weight maintenance. This implies that the food items with increased use have at least suggestive scientific evidence on prevention of weight gain and/or type-2 diabetes. Respectively, foods with decreased use have at least suggestive evidence on increasing weight gain and/or type-2 diabetes. The main principles are shown in Table 2.

Table 2: Principles of the dietary interventions

	**High protein (25 E%)<br>Moderate carbohydrate (45 E%)<br>Low GI (≤50) diet**	**Moderate protein (15 E%)<br>High carbohydrate (55 E%)<br>Medium GI (≥56) diet**
**Comparison between the groups**	• Protein intake higher<br>• Carbohydrate intake lower<br>• GI lower	• Protein intake lower<br>• Carbohydrate intake higher<br>• GI medium
**Food items with increased use (vis-à-vis the other group)**	• Whole-grain cereals with low GI<br>• Pasta<br>• Low-fat milk products<br>• Poultry<br>• Fish<br>• Legumes	• Whole-grain cereals with moderate/high GI, e.g. bread<br>• Potatoes, sweet potato, cous-cous, rice
**Similar use**	• Most fruits and vegetables<br>• Vegetable oils, margarine<br>• Red meat (decreased in both)<br>• Sugar-sweetened beverages (decreased in both)	

To achieve the intended difference in the ratio between carbohydrates and protein, we will provide the participants with examples of daily eating plans with foods in appropriate proportions to reflect the macronutrient and GI requirements of the two different interventions. Suggestions will be given for food exchanges in order to allow variety (self-selection), whilst preserving the required macronutrient and GI levels. Daily eating plans will represent a range of total energy levels so that participants can select the portion size they favour (i.e. *ad libitum* principle). In addition, both groups will receive more

general instructions on foods that do not have to be limited and those that do. The plate model will also be used to demonstrate visually the correct ratio between protein and carbohydrates, based on commonly consumed foods. Finally, further advice will include how to respond to feelings of hunger and to problems of weight relapse. The advice will be directed to the participants at a group level, so that the main intended group differences in food use and nutrient intakes are maintained. The principles are explained in details in SOP-2.

Participants in each group will receive written material on how to compose the diet. This material includes a cooking book specifically designed for PREVIEW. Each diet group will be provided with the version of the cooking book with recipes that are appropriately formulated to meet the requirements of their specific diet group (i.e. HP/LGI recipes or MP/MGI recipes). Moreover, one staff-member from each study site will be trained in preparing meals designed for this study at a workshop arranged in Copenhagen by Meyers Madhus, who is a partner in PREVIEW.

Dietary compliance is assessed by a Dietary compliance questionnaire at weeks 26, 52, 104, and 156.

- ➔ **Ins-12: Re-feeding strategy**
- ➔ **SOP-2: Diets**
- ➔ **Q-3 and Q-3-C: Dietary compliance questionnaire**
- ➔ **Cooking books for HP and MP diets (Ins- 8a and Ins-8b)**

<u>Scheduling of visits</u>

The adult participants have group meetings counselled by a dietician (or another qualified experienced health-care professional) on study weeks 8 (end LCD/ beginning of the randomized intervention), 10, 12, 16, 20, 26, 32, 44, 52, 64, 78, 104, and 130. For children and adolescents, individual meetings take place at study weeks 8, 12, 16, 20, 26, 32, 44, 52, 64, 78, and 104.

For adults, the first 4-5 meetings are used to support initiation of the diet and the remaining meetings are targeted towards maintaining new habits. The group supervision methods are enforced by health psychology theory: Social Cognition Theory (SCT), Trans-theoretical model (TTM), Self-determination theory (SDT), tailored specifically to support the PREVIEW study. All relevant staff at each of the study sites will be given appropriate training in order to execute these methods.

A typical group meeting will take up to 120 minutes. The participants will be in small groups (6-12 participants per group). Participants of the two dietary interventions (HP and MP) will, to the extent possible, not be mixed in the group meetings. The first part of each meeting is devoted to dietary supervision. The second part of the meeting is devoted to physical activity. Like for diet, the two physical activity programmes (HI and MI) are not mixed.

All participants may also be approached between each group meetings with phone call, SMS and/or e-mail. Moreover, participants are also encouraged to contact the site-staff if they have any questions related to diet, physical activity programme or any other items of the study.

Dietary intake is recorded by 4-d dietary records and compliance of protein intake by analysing nitrogen and/or urea in urine (see sections 7.6.4 and 7.6.6). The recording and urine collection take place on week 0, 26, 52, 104 and 156.

- ➔ **SOP-3: Group Supervision [Group supervision based on Theories of Health Psychology] (adults)**
- ➔ **SOP-3a: Group Supervision – Children [Group supervision based on Theories of Health Psychology]**

5.2.5 Physical activity interventions

All participants will be randomized to one of the two physical activity interventions, HI = high-intensity (high-intensity) or MI = moderate-intensity (Table 3). For children and adolescents it is, however, allowed to take exercise from both types of activities in order to increase adherence to the study. Measured heart-rate (by heart-rate monitor or palpitation) is the principle way of determining MET-levels.

Physical activity is in general unsupervised. Hence, the participants can choose from several options with similar level of metabolic turnover (energy expenditure divided by resting metabolic rate, i.e., MET values). The two physical activity interventions will have roughly similar target energy expenditure (1000 kcal/wk), but the specific advice is based on the Centre for Disease Control (CDC) recommendations on at least 75 min high-intensity or 150 min moderate-intensity physical activity weekly (19). The main principles of the physical activity regimens are given in Table 3:

Table 3: Principles of the physical activity interventions

	High-intensity physical activity	Moderate-intensity physical activity
**Heart rate**	• 76-90% HRmax<br>• 61-80% HRR	• 60-75% HRmax<br>• 45-60% HRR
**Examples of activities**<br>**(these may vary depending on the fitness level of the participant)**	• Bicycling, high effort<br>• Strenuous ball games<br>• Aerobics with very high effort, e.g. with extra weights<br>• Jogging>8 km/h<br>• Swimming, high effort, laps<br>• Cross-country skiing	• Bicycling, moderate effort<br>• Leisurely ball games<br>• Most conditioning exercises (aerobic, power yoga, etc.)<br>• Brisk walking (4-6 km/h)<br>• Swimming, recreational<br>• Downhill skiing
**Weekly duration (in total)**	• 75 min	• 150 min
**Weekly frequency**	• 2-3 times	• 3-5 times
**Daily duration (guideline)**	• 25-40 min	• 30-50 min (may be broken down into shorter sessions)
**Additional exercises**	• Muscle conditioning exercises, by using own weight (e.g. push-ups, sit-ups): twice weekly at home, 15-20 min per session.<br>• Stretching: twice weekly, 15-20 min per session	• Muscle conditioning exercises, by using own weight (e.g. push-ups, sit-ups): twice weekly at home, 15-20 min per session.<br>• Stretching: twice weekly, 15-20 min per session

HRmax = max heart rate, defined as 220 – age (220 in children under 16 y)
HRR = heart rate reserve, defined as the difference between measured resting HR and estimated HRmax

In the beginning of the weight-maintenance phase (week 8), the participants, assisted by an exercise instructor (or another experienced health-care professional), plan their personal physical activity programme. In the group sessions, the participants are also instructed on basic principles of increasing physical activity. Moreover, they are taught problem-solving techniques, that is, how to overcome urges to refrain from planned physical activities. Stretching and home-based muscle-conditioning exercises are also supervised in a group-based session. All physical activity sessions are planned to be at the same visit with the dietary supervision at the site-centre.

The first month of the interventions (study weeks 9—12) is used to increase the weekly physical activity volume to 75 min. The intensity is kept moderate in all participants.

The next month (study weeks 13—16) is used to reach the intended prescribed program. For the HI-groups, this means that the volume (weekly duration, i.e., 75 min) is kept constant while the intensity is gradually increased so that in the end of this period all physical activity sessions should have an intensity of at least 6 METs. There will be no intensity change for the MI-group, but the volume is gradually increased from 75 min to 150 minutes per week during this 4-week period.

During every visit, questions related to physical activity are also discussed. All research centres have a physician who the participants may consult with any problems related to the study (such as exercise-induced injuries). Moreover, all participants will receive written material and have access to a "Frequently Asked Questions" provided by the PREVIEW webpage.

Physical activity will be assessed by Baecke questionnaire, training log books (7 days) and accelerometers (7 days). The assessment points are the same as for dietary monitoring, that is, weeks 0, 26, 52, 104 and 156 (last one only for adults). On the same CID-visits, VO_2 max measurements will be performed in some sites in a sub-set of participants (see later).

➔ **SOP-4: Physical activity programs**

5.3 Blinding

Due to the characteristics of the study design, any blinding of the dietician, exercise instructors or participants is not possible. Each dietician and physical activity trainer will be responsible for guidance of participants in all intervention groups.

Any data analysis obtained prior to study termination will not influence the on-going study and hence the final outcome of the study in order to exclude any bias introduced by the study personnel. We make all efforts to blind the staff (e.g. lab technicians) taking measurements, particularly on the CID's. Moreover, the statistical analyses of the main outcome variable will be done without breaking the group-assignment code before the analyses are finalized.

6. PARTICIPANTS

6.1 Number of participants overall and in different sites

Participants will be recruited in each of the 8 participating countries from the general population and with no specific requirements on ethnicity. The statistical power is based on a total population of approximately 2,500 adult participants eligible to undergo the 8 weeks weight-reduction period. This calculation is based on an assumption of 75% of subjects achieving an 8% weight loss during the LCD as well as estimated drop-out rates during the 3 y intervention (LCD + weight maintenance phase).Thus, up to 2,500 adult subjects will be recruited. Furthermore, 150 children/adolescents will be included in the 2-y study. The aimed distribution between age groups and sites is given in Table 4.

Table 4: Goal of participants to be included (revised in 2014)

	Total	UCPH	HEL	UM	UNOTT	UNAV	MU	UNSYD	UOA	SU
Children and adolescents, 10-18 y	150			100		25				25
Adults, 25-70 y	2,500	360	315	215	315	290	375	315	315	
**Sum**	2,650	360	315	315	315	315	375	315	315	25

6.2 Inclusion Criteria

The diagnosis of diabetes and other categories of glucose intolerance will be based on criteria adopted by the WHO in 1985 and 2006, and used in the DPS study (Eriksson et al. 1999). The following inclusion criteria apply for screening (shown by age groups):

Table 5: PREVIEW intervention – Inclusion criteria

**Inclusion criteria**	**Children and Adolescents**	**Adults**
1. Age	10 - 18 years	25 - 70 years
2. Overweight or obesity status	Age-adjusted value corresponding to BMI ≥ 25.0 kg/m^2 (Cole et al. 2000)	BMI ≥ 25.0 kg/m^2
3. Pre-diabetes	Since the prevalence of pre-diabetes among children with overweight or obesity is low, it is not feasible to include exclusively pre-diabetic children (according to criteria of the IDF).<br>Therefore insulin resistant overweight/obese children will be included, defined as:<br>• HOMA-IR ≥ 2.0 for Tanner stage > 2<br>• No defined HOMA-IR for Tanner stage 1 and 2, since HOMA-IR increases with Tanner stage and, even for a HOMA-IR < 2 at T1 and T2 improvement is possible<br>.	The criteria from WHO/IDF (International Diabetes Foundation) for assessing pre-diabetes will be used as the formal inclusion criteria, i.e. having:<br>• Impaired Fasting Glucose (IFG): Fasting venous plasma glucose concentration 5.6 - 6.9 mmol/l or<br>• Impaired Glucose Tolerance (IGT): Venous plasma glucose concentration of 7.8 – 11.0 mmol/l at 2 h after oral administration of 75 g glucose (oral glucose tolerance test, OGTT), with fasting plasma glucose less than 7.0 mmol/l.<br>Due to potential between-lab variation (local assessments), HbA1c is not used as an inclusion criteria in the screening.
4. Informed consent	Required from all the participants, or both their parent/guardian	Required
5. Smoking	Smoking is allowed, provided subjects have not recently (within 1 month) changed habits. However, smoking status is monitored throughout the study and used as a confounding variable.	
6. Motivation	Motivation and willingness to be randomized to any of the groups and to do his/hers best to follow the given protocol	
7. Other	Able to participate at CID's during normal working hours.	

6.3 Exclusion criteria

Based on interview and/or questionnaire, individuals with the following problems will be excluded:

Medical conditions as known by the subjects:

1) Diabetes mellitus (other than gestational diabetes mellitus);

2) Significant cardiovascular disease including current angina; myocardial infarction or stroke within the past 6 months; heart failure; symptomatic peripheral vascular disease;

3) Systolic blood pressure above 160 mmHg and/or diastolic blood pressure above 100 mmHg whether on or off treatment for hypertension. If being treated, no change in drug treatment within last 3 months. There will be no criteria for blood pressure limits in children and adolescents;

4) Advanced chronic renal impairment;

5) Significant liver disease e.g. cirrhosis (fatty liver disease allowed);

6) Malignancy which is currently active or in remission for less than five years after last treatment (local basal and squamous cell skin cancer allowed);

7) Active inflammatory bowel disease, celiac disease, chronic pancreatitis or other disorder potentially causing malabsorption;

8) Previous bariatric surgery;

9) Chronic respiratory, neurological, musculoskeletal or other disorders where, in the judgement of the investigator, participants would have unacceptable risk or difficulty in complying with the protocol (e.g. physical activity program);

10) A recent surgical procedure until after full convalescence (investigators judgement);

11) Transmissible blood-borne diseases e.g. hepatitis B, HIV;

12) Psychiatric illness (e.g. major depression, bipolar disorder).

Medication:

13) Use currently or within the previous 3 months of prescription medication that has the potential of affecting body weight or glucose metabolism such as glucocorticoids (but excluding inhaled and topical steroids; bronchodilators are allowed), psychoactive medication, epileptic medication, or weight loss medications (either prescription, over the counter or herbal). Low dose antidepressants are allowed if they, in the judgement of the investigator, do not affect weight or participation to the study protocol. Levothyroxine for treatment of hypothyroidism is allowed if the participant has been on a stable dose for at least 3 months.

Personal/Other:

14) Engagement in competitive sports;

15) Self-reported weight change of >5 % (increase or decrease) within 2 months prior to screening;

16) Special diets (e.g. vegan, Atkins) within 2 months prior to study start. A lacto-vegetarian diet is allowed;

17) Severe food intolerance expected to interfere with the study;

18) Regularly drinking > 21 alcoholic units/week (men), or > 14 alcoholic units/week (women);

19) Use of drugs of abuse within the previous 12 months;

20) Blood donation or transfusion within the past 1 month before baseline or CID's;

21) Self-reported eating disorders;

22) Pregnancy or lactation, including plans to become pregnant within the next 36 months.

23) No access to either phone or Internet (this is necessary when being contacted by the instructor's during the maintenance phase);

24) Inadequate understanding of national language;

25) Psychological or behavioural problems which, in the judgement of the investigator, would lead to difficulty in complying with the protocol.

Laboratory screening:

If all of the above criteria are satisfied, the participant is eligible for a glucose tolerance test (blood at 0 and 120 min), and blood glucose concentrations are analysed immediately (Haemocue). In addition, full blood count, urea, and electrolytes may be analysed as a further safety evaluation at the discretion of the investigator.

ONLY IF the glucose tolerance test meets the entry criteria for the study, the remaining samples are sent to the local laboratory for a safety check, with the following exclusion criteria:

26) Haemoglobin concentration below local laboratory reference values (i.e. anaemia).

27) Creatinine >1.5 times Upper Limit of Normal (local laboratory reference values).

28) Alanine Transaminase (ALT) and/or Aspartate Transaminase (AST) >3 times the Upper Limit of Normal (local laboratory reference values)

    Or any other significant abnormality on these tests which in the investigators opinion may be clinically significant and require further assessment

29) Electrocardiography (ECG). Any abnormality which in the opinion of the investigator might indicate undiagnosed cardiac disease requiring further assessment (e.g. significant conduction disorder, arrhythmia, pathological Q waves). This is done in adults 55-70 years of age.

After LCD phase (in adults):

30) Failure to reach at least 8% weight reduction during the LCD-phase. This leads to exclusion from the intervention.

6.4 Recruitment and Informed Consent procedures

An adequate number of potential participants are secured by using multiple ways of recruitment, i.e. newspaper advertisements, newsletters, waiting lists and via other written or electronic media, contacts with national and local obesity and diabetes association and direct contact with primary and occupational health care providers. The final procedures are at the responsibility of the site Principal Investigators.

We will allow competitive recruitment. Thus, some centres may recruit more than planned, to compensate for less successful centres.

Individuals interested in the study are invited to contact the site-centre by e-mail or SMS. A limited time-slot for telephone enquiries is also given. Age, body weight and height should be given in the mail/SMS for preliminary exclusion of clearly too lean participants (Figure 1).

The individuals are then contacted by e-mail or phone for a short pre-screening interview. In this interview, most inclusion and exclusion criteria are queried. The interview will include the Finnish Diabetes Risk Score (20). If the subject has a score ≥ 12, he or she is eligible to continue to the next steps in the screening process. Based on the interview potential participants are sent a written description of the study and invited to an information meeting. The Diabetes Risk Score is only used for adults.

At the information meeting the study is explained carefully and questions are answered. Individuals, who are still interested in participating, can sign the informed consent form at the meeting. Hereafter, the screening visit is scheduled. Those who need more time to consider, are allowed to take the consent form home and return it later, if they decide to participate.

The informed consent form must always be signed and dated both by the participant and the investigator or by the project worker - who have been delegated by the Investigator in accordance to the delegation log and qualified to perform the oral information - before any measurements can be performed. Participants will be informed that the consent form includes their consent to allowing auditing and/or monitoring of their data by the appropriate regulatory authority, an independent monitor, or a Sponsor/consortium representative.

After the potential participant has signed the informed consent form, the laboratory screening begins. This starts by measurements of weight, height, resting blood pressure and ECG (in the oldest age-group, or even younger if this is deemed necessary by the investigator). Then a fasting blood sample is taken from the ante-cubital vein (for assessment of exclusion and inclusion criteria). A small aliquot from the venous sample is immediately analysed by Hemocue in order to identify possible diabetics. At 2 h, a new venous blood sample is taken (simplified OGTT). The 0 and 2 h Hemocue analyses are used to exclude clearly non-pre-diabetics, on one hand, and evident diabetics, on the other hand. For those eligible (based on the glucose analyses), the blood samples are then analysed with regard to exclusion criteria 26—29.

If a child is found eligible for the study, his or her parent may also participate, if he/she fulfils the inclusion and exclusion criteria of adult participants. Full screening, preceded by a signed informed consent, is needed from the adult.

6.4.1 Re-screening

A participant may be re-screened in the following cases:

- Diagnosed type-2 diabetes at PREVIEW Screening (fasting blood glucose ≥7.0 and/or 120 min blood glucose in OGTT ≥ 11.1 mmol/l):

Re-screening with the simplified OGTT within 2—4 weeks including at new screening visit. If type-2 diabetes is again diagnosed, the participant is not eligible and must be referred to his or her general practitioner (GP). If type-2 diabetes is not diagnosed, the participant can be included in the study.

- Hypertension: Re-screening is possible during the same day or within a week:

  If the initial screening and re-screening identifies unregulated hypertension (exclusion criteria), the participant will be referred to his/her own general practitioner (GP) to undergo further evaluation and possibly to receive antihypertensive medication. If the blood pressure becomes well regulated for 2-3 month, the participant can be invited to a full new screening in the study. A new signed informed consent is needed.

- Any other re-screening should be done at the discretion of the PI (but according to the exclusion/inclusion criteria), including a new signed informed consent form with a new ID.

➔ **Q-1 and Q-C-1: Screening interview**

➔ **SOP-5a: Pre-screening and screening, adults**

➔ **SOP-5b: Pre-screening and screening, children**

➔ **SOP-5c: OGTT during Screening**

➔ **SOP-5d: Guidance for Type-2 diabetes diagnose in PREVIEW study**

➔ **SOP-23a: CRF Adults**

➔ **SOP-23b: CRF Children**

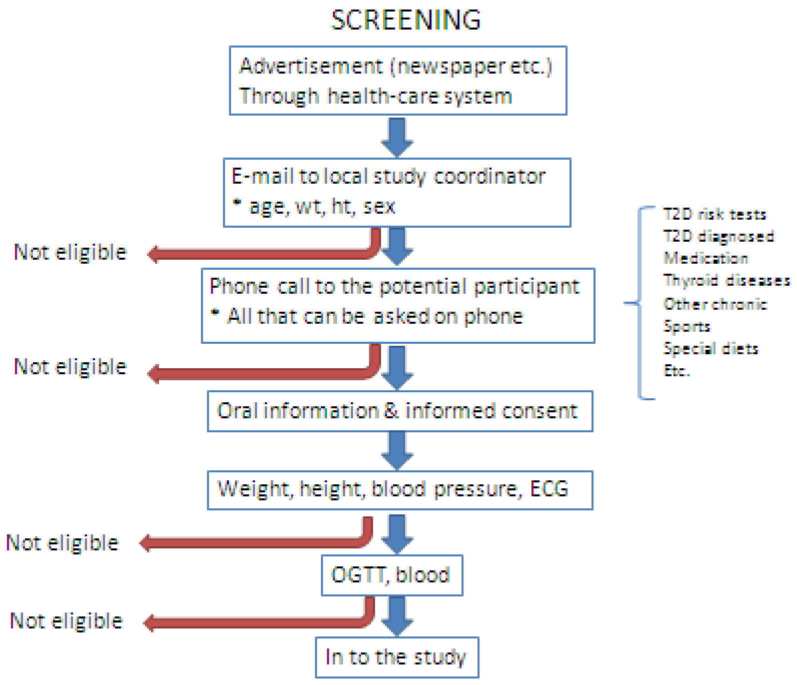

Figure 1 - The screening process

6.5 Procedures for exclusion/withdrawal from the study

Participation in the study is voluntary, and subjects have the right to withdraw from the study at any time without providing a reason, and with no loss of benefits to which the subject is otherwise entitled. The subject will be informed that already collected data can be used and will be included in the primary Intention to Treat (ITT) analysis. If a study subject chooses to withdraw, the study personnel must be informed immediately, and every effort should be made to complete the final visit assessments as soon as possible (EOT, visit 19 for adults and visit 17 for children/adolescents). The reason for discontinuation should be documented in the electronic Case Report Form (eCRF).

The Investigator has the right to terminate participation of any subject at any time if deemed in the subject's best interest.

Examples of possible reasons for premature withdrawal of a study subject include:

- Subject withdraws consent for personal reasons
- Subject's general condition contraindicates continuing the study, as judged by the study personnel or the medical expert
- Serious Adverse Event (SAE), which in the opinion of the investigator will be incompatible with continuation of the study
- Pregnancy
- Lost to follow-up
- Other reasons as determined by the Investigator
- Data collected from participants prior to withdrawal from the study are used in the final study analysis, but only if this is not precluded by local/national ethical regulations.

➔ **SOP-23c: End of Trial/Termination**

7. METHODS

7.1 Time-window and general principles for data collection

The measurements follow a weekly time-schedule, as outlined in the flow chart. Although the aim is to make each measurements/data collections precisely as scheduled, to ensure as complete a data collection as possible we strive to follow the following time-windows:

- Week 8: -3 to +5 days
- Week 10, 12, 16, 20, 26: ±1 weeks

- Week 52: ±2 weeks
- Any of the following measurement points: ± 4 weeks

When possible (e.g. anthropometric measurements, blood pressure, blood specimen draw, etc.), the data collection follows procedures and descriptions of the European Health Examination Survey (EHES) manual (http://www.ehes.info/manuals/EHES_manual/EHES_manual.htm).

7.2 Collection of basic and contact information

The following background data are collected (and noted with pen-and-paper) during the initial telephone interview:

- Name
- Age
- Gender
- Contact information (address, telephone, email)
- Main reason for exclusion / non-participation (if relevant)

7.3 Demographic data collection

The following background data are collected during the screening visit:

- Marital status
- Education
- Classified annual income (in analyses this is related to country-wise median income)
- Medical history, including gestational diabetes

➔ **Q-2 and Q-C-2: Background-data questionnaire**

➔ **SOP-23a: CRF Adults**

➔ **SOP-23b: CRF Children**

7.4 Anthropometric data and body composition

Anthropometric data include body weight, height, sitting height, circumferences for waist, hip and thigh, body composition and fat distribution.

7.4.1 Body weight and height

Body weight is measured with an empty bladder and wearing underwear or other light clothing. During CIDs body weight is measured when the participant is in the fasting state. Two measurements are taken to the nearest 0.1 kg. This process is repeated and the average of each of the two weights is used in the analysis.

For measurement of height the participant is required to remove shoes and have his/her heels, buttocks and upper part of the back remaining in contact with a wall-mounted stadiometer to the nearest 0.5 cm. The average of the two heights is used in the analysis.

For measurement of sitting height, the participant is required to sit on a flat, wooden shelf, placed on a seat and have his/her buttocks and upper part of the back remaining in contact with a wall-mounted stadiometer measuring to the nearest 0.5 cm. The stadiometer gives the height of the shelf (a) the perfect horizontal height of the subjects' head (b): sitting height is b-a. The average of two sitting height measurements is used in the analyses.

Sitting height is measured in order to calculate leg-length (height – sitting height). For adults this is done at a random visit. For children / adolescents it is done at all CID visits (weeks 0, 8, 26, 52, 78, 104).

Weight is measured at screening and in weeks 0, 2, 4, 6, 8, 10, 12, 16, 20, 26, 32, 44, 52, 64, 78, 104, 130, and 156. For children, weight is measured every 4 weeks up to week 20 and then at similar time points as for the adults until week 104 inclusive.

Height is measured in adults in weeks 0 and 156 and in children in weeks 0, 26, 52, 78, and 104.

7.4.2 Waist, hip, and thigh circumference

Waist, hip and thigh circumferences will be measured to the nearest 0.5 cm with the same tape, if possible, with the subject standing. Two measurements are taken and the mean is used.

Waist measurement will be taken midway between the lower rib and iliac crest, at the end of expiration.

Hip circumference will be measured as the largest circumference in the area around the buttocks.

The circumferences are measured with an empty bladder. During CIDs, circumferences are measured in the fasting condition in the morning.

Mid-thigh circumference is taken on the right side of the body. The circumference measure is taken at the level of the mid-point on the lateral (outer side) surface of the thigh, midway between trochanterion (top of the thigh bone, femur) and tibiale laterale (top of the tibia bone).

Waist, hip and thigh circumferences are measured in weeks 0, 8, 26, 52, 104, and 156 (the last only in adults).

7.4.3 Body composition and liver fat content

Body composition:

Measurement of body composition is done, if possible, by dual X-ray absorptiometry (DXA), underwater weighing, BodPod, or deuterium dilution, depending on the availability in each site-centre. If this is not possible, a bioelectrical impedance method is used. The measurements are done according to the manufacturer's instructions, and when the participant is in the fasted state. The same device and software should be used throughout the entire study and must be calibrated according to manufacturer's instructions. Fertile women will be tested for pregnancy before DXA.

Body composition measurements in all subjects are done in weeks 0, 8, 26, 52, 104, and 156 (the last only in adults).

Liver fat content:

Assessment of liver fat content by MRI (Magnetic Resonance Imaging) and H-MRS (Hydrogen Magnetic Resonance Spectroscopy) is done in a sub-group (Sub-group A) of 40 subjects: 20 from HPMI and 20 from MPMI group, at UM.

MRI/H-MRS is a non-invasive and non-irradiant imaging technique, which will be used to determine liver fat content.

MRI and H-MRS measurements take place at weeks 0, 26 and 104.

- ➔ **SOP-6a: Body weight and height, sitting height, waist, hip and thigh circumference**
- ➔ **SOP-6b: Body composition**
- ➔ **SOP-6c: Liver fat content**

7.5 Blood pressure and heart rate

Systolic and diastolic blood pressure (BP) will be measured using a validated automatic device on the right arm after 5-10 min rest in a resting position. The measurement is taken three times with a 1 min rest between, and the reading is recorded to the nearest 1 mmHg. A mean value of these three readings is used. High-intensity physical activity, coffee or smoking is prohibited for 12 h before the measurement. The device should be overhauled and calibrated regularly (at least annually). Resting heart rate and – if possible – heart rate variability is measured.

An ECG measurement will be done at screening in 55-70 y old subjects, as a precautionary measure to identify individuals for whom particularly the high-intensity physical activity program could be a health risk.

Blood pressure is measured at screening (only adults) and in weeks 0, 8, 26, 52, 78, 104 and 156 (the last only in adults).

Heart rate is measured in weeks 0, 8, 26, 52, 78, 104 and 156 (the last only in adults).

- ➔ **Ins-2: Instruction to participants regarding blood pressure assessment**
- ➔ **SOP-8: Blood pressure and heart rate assessment**
- ➔ **SOP-9: ECG (Electrocardiography)**

7.6 Biological Samples

7.6.1 OGTT and blood sample

2-h Oral Glucose Tolerance Test (OGTT):

Each adult participant will take an oral load of 75 g glucose (commercial product) dissolved in 300 ml water within a 5 min period. The participants will remain semi-recumbent and resting throughout the procedure, and must be in a fasted state before the start of the test (except for 1 dL of water). Furthermore, no foods and drinks (other than the test load) are allowed until completion of the test. Blood

samples will be obtained for glucose assessment at 0, 30, 60, 90 and 120 and for insulin assessment at 0, 30, and 120 minutes after drinking the liquid glucose solution.

Glucose from 0 and 120 min samples will be analysed immediately with HemoCue and plasma glucose estimated by multiplying the HemoCue Blood glucose value by 1.11. Glucose from 0, 30, 60, 90 and 120 min will be analysed centrally later (THL).

If diabetes appears to have developed on the basis of either the 0 or 120 min values then the participant will be re-tested again within 2 to 4 weeks from the CID visit. If type-2 diabetes is not verified by HemoCue at the re-test, the participant can continue in the study. If type-2 diabetes is verified by Hemocue at the re-test, the participant is excluded from the study and referred to his/her own general practitioner.

<u>Fasting blood samples:</u>

Fasting blood is drawn from both adults and children from the antecubital vein when the participant is in a fasted state, that is, before the 2-h OGTT for adults. The following analyses from the blood specimen belong to the basic PREVIEW protocol:

- Glucose
- Insulin
- C-peptide
- HbA1c
- Total, HDL- and LDL-cholesterol, triglycerides
- CRP
- Liver enzymes

The OGTT and fasting blood samples are taken at screening (only 0 and 120 min) and week 0 (baseline, CID 1), 8 (CID 2, only fasting), 26 (CID 3), 52 (CID4), 78 (CID 5, only fasting), 104 (CID 6) and 156 (CID 7 in adults only).

→ **Ins-1: Instruction to participants regarding blood specimen draw and OGTT**

→ **SOP-7: Lab manual**

→ **SOP-21: OGTT**

7.6.2 Sampling and analyses of DNA and RNA

For DNA extraction, buffy coat samples are collected from Ethylenediaminetetraacetic acid (EDTA) blood following the instructions in the SOP.

For isolation and purification of intracellular RNA, 1x2.5 ml whole blood is collected in Paxgene Tube. PAXgene Blood RNA Kit is used for RNA extraction (yield 3-7 g RNA).

The intended use of the samples is epi/genetic analyses in relation to obesity and associated diseases.

Collecting material for genetic and epigenetic analyses will be done in accordance to the local Ethical Boards.

➔ **SOP-10a: Collection of DNA samples**

➔ **SOP-10b: Collection of RNA samples**

7.6.3 Amount of blood sampled

At all CID visits, fasting samples of blood are taken for analysis of glucose, insulin, C-peptide, lipids and HbA1c. At visits CID1, CID4 and CID7 fasting samples for DNA, RNA are also taken, but only in the sites where this is possible (e.g. where the ethical committees/local law allows this). Two fasting samples are also taken for storage. Further, in a sub-group of 100 elderly (+55 y) samples for analyses of serum creatinine are also taken at CID1, CID4, and CID7 (sub-group G).

For the OGTT (done for adults at all CID visits, except CID5) samples are taken at 0, 30, 60, 90 and 120 minutes for analyses of glucose. Insulin will be analysed at 0, 30 and 120 min. At all time points samples are also taken for storage.

The maximum amount of blood taken at a CID visit is 100 ml blood for adults.

For children neither OGTT, DNA, nor RNA samples are taken. Therefore the total volume for children is not more than 50 ml each time.

A description of the detailed methodology for laboratory assessments in the lab manual is provided by the central lab (THL) and by any other laboratories participating in PREVIEW. These descriptions are included in a separate document.

Until the serum samples are sent to the central lab for analysis, all of them will be listed on study specific sample logs and stored at the clinical centres. The storage condition for serum samples for analysis will be at -80° Celsius. Freezers will be temperature monitored on a daily basis or connected to an alarm.

Blood analyses are done both during and after the study.

➔ **SOP-7: Lab Manual**

7.6.4 Urine samples

A 24-h urine sample will be collected for assessment of nitrogen or urea (biomarker for protein intake) in adults. An aliquot (2 x 1 ml) of the urine is also taken for local storage for later analyses. The collection is done by separate instructions to the participants and the sample is treated and stored by following a SOP in all sites.

The urine sample is collected at weeks 0, 26, 52, 104, and 156.

In a sub-group of 800 subjects consisting of 200 subjects from 4 sites (UCPH, HEL, UNOTT, and UNAV), urine from baseline (CID1) and month 12 (CID4) will be analysed for metabolomics profile (Sub-group D).

In a sub-group of 100 subjects (+55 y) urine is also analysed for albumin at CID1, CID4 and CID7 (subgroup G).

➔ **Ins-3: Instruction to participants regarding urine sample collection**

➔ **SOP-11: Urine sample collection, analysis and handling**

7.6.5 Faecal samples

Full faecal samples from 3 consecutive days are collected from a sub-group (Sub-group B) in 2 centres (UOA and HEL, n=100 in each centre). At least 2 days of complete collection are needed for a valid collection. The samples are used for assessment of colon cancer risk markers, i.e. phenolic metabolites, short-chain fatty acids (SCFA), nitrogenous compounds. Subjects are recruited from all four groups on a consecutive invitation basis. Moreover, samples from the same subjects are stored for analysis of microbiota. Faecal samples will be collected during weeks 0 and 52.

➔ **Ins-4: Instructions to participants on collecting faeces**

➔ **SOP-12: Faecal sample collection and handling**

7.6.6 Storage and analysis of biological samples

The storage condition for serum, urine and faeces samples for analysis will be at -80° Celsius. Freezers will be temperature monitored on a daily basis or connected to an alarm.

The biological samples will be reported to the appropriate regulatory authority in each participating country. All biological samples will solely be stored and analysed for the purposes described in this protocol. After complete analysis and data processing on the protocol related samples, all biological samples will be destroyed. Procedures for collecting, storing etc. will be done in accordance with current legislation in each country.

The stored samples will serve to carry out additional analyses giving more insight to development of type-2 diabetes and cardiovascular diseases. These measurements include e.g. vitamins, adipocytokines, metabolomics, microbiota etc., and will be carried out when external funding is obtained.

The additional samples for analyses connected to the sub-studies will be stored at the site.

7.7 Assessment of food consumption, nutrient intakes and GI

Dietary assessment (nutrient intakes, food consumption) is done by repeated 4-day food records. The participants are asked to fill in a complete record of everything they eat and drink (time, place, exact description of eaten foods, and amount) during the given days (3 week days and 1 weekend day). At least 2 week days and 1 weekend day of acceptable quality is required for eligible data collection. The procedure is first instructed in a group session. The participants are encouraged to use a small scale to weigh their foods, but this is not compulsory. The food diaries are returned personally to a study technician (dietician or a corresponding professional) who immediately checks the diaries and asks clarifying questions, if needed. All efforts are done to combine group meetings with returning of e.g. food diaries, in order to minimize the burden of the study to the participants.

The food records are converted to dietary intakes and food (group) use in PREVIEW site, by using national or other appropriate nutrient composition data tables and software. The definition of food groups will be agreed by the research team before the analyses in order to ensure comparable data in all sites.

The 4-d food diaries are collected during weeks 0, 26, 52, 104 and 156 (the last only in adults).

➔ **Ins-5: Instruction to participants for filling in food diaries**

➔ **SOP-13: Diet analysis**

7.8 Assessment of dietary restraint

We will use the full-length version of the Three Factor Eating Questionnaire (TFEQ) to assess all of the characteristics of dietary restrained. The TFEQ's are returned personally to a study technician who immediately checks the questionnaires and asks clarifying questions, if needed.

The TFEQ's are filled in during weeks 0, 8, 26, 52, 104 and 156 (the last only in adults).

➔ **Q-4 and Q-4-C: TFEQ**

7.9 Assessment of appetite

Subjective appetite ratings (hunger, satiety, fullness, prospective food consumption, thirst) are measured using Visual Analogue Scale, VAS scores (100 mm scale). The assessments are done during weeks 0, 8, 26, 52, 104 and 156 (the last only in adults).

➔ **Q-5 and Q-5-C: VAS for assessment of appetite**

➔ **SOP 14: VAS for assessment of appetite**

7.10 Assessment of physical activity

Physical activity will be analysed by two methods: by the self-administered (subjective) Baecke questionnaire, and by 7-day accelerometry recording for 24 hours daily (objective method).

7.10.1 Baecke questionnaire and physical activity log

The Baecke questionnaire measures work, sport and leisure activities using 22 frequency questions. The outcome variables are three respective indexes. The questionnaires are returned personally to a study technician who immediately checks the questionnaires and asks clarifying questions, if needed.

In addition, the participants fill in a 7-day physical activity log with more emphasis on the prescribed physical activities. At least 4 days (3 weekdays and 1 weekend day) are needed for eligible data collection.

The Baecke questionnaires and physical activity logs are filled in during weeks 0, 26, 52, 104 and 156 (the last only in adults).

➔ **Q-6 and Q-C-6: Baecke questionnaire**

➔ **Q-7 and Q-C-7: Physical activity log**

➔ **SOP-4a: Physical Activity Log**

➔ **Ins-13: Physical Activity Log**

7.10.2 Accelerometry

In all centres, physical activity and sleep are assessed by accelerometers. With this method, the participants are asked to wear the instruments 24 hours/day (excluding during showering, swimming, etc.) for 7 full days, including 2 weekend days. We are aiming for 7 days and require at least 4 valid days (i.e. at least 10 hours of wearing), including at least one weekend day for analyses. The participants are first introduced to wearing and using the accelerometer. During the first data collection, an extra day is used for familiarization. We will try to contact the participants 1-2 times during the data collection to make sure they have no problems.

The accelerometer data are collected during weeks 0, 26, 52, 104, and 156 (the last only in adults).

➔ **Ins-6: Instructions to participants for using accelerometers**

➔ **SOP-15: Accelerometer data collection**

7.11 Assessment of physical fitness

Physical fitness (VO_2 max) will be assessed in an adult sub-group (sub-group C) in UM (n=40), UNOTT (n=20), UCPH (n=40), and UNAV (n=20) by using an incremental treadmill or ergometer test with measurement of respiratory gases (indirect calorimetry). The recruitment for the VO_2 max test is by inviting consecutive participants until the needed numbers of volunteer participants with at least roughly equal group distribution (HI vs. MI) are achieved. The outcome variables are VO_2 max and VO_2 and respiratory quotient (RQ) at a predetermined submaximal load. The test is submaximal up to an estimated 85% of VO_2 max.

The physical fitness data are collected during weeks 0, 26, and 104.

➔ **Ins-7: Instructions to participants regarding physical fitness tests (VO_2 max)**

➔ **SOP-16: Assessment of physical fitness (VO_2 max)**

7.12 Assessment of Adverse events and Concomitant Medicine

Adverse events (AEs) will be registered during all CID visits. The participant will be asked if he/she has noticed any unfavourable events since last evaluation and if the subject answers “Yes”, the AE form in the eCRF will be filled in for each separate AE. The form will cover onset, end, intensity, causality, action taken and outcome of the AE.

Use of concomitant medication, as well as herbal medicine, Chinese medicine and dietary supplements that in the opinion of the investigator can affect any of the primary study outcomes, will be registered at screening visit and at each of the CID visits. The participant will be asked if he/she has taken any new medicine since last evaluation and if the subject answers “Yes”, the concomitant medication in the eCRF will be filled in for each product.

- ➔ **SOP-23a: CRF Adults**
- ➔ **SOP-23b: CRF Children**
- ➔ **SOP-24: Concomitant Medication**

7.13 Questionnaires related to moderators, mediators, behaviour and social environment

The PREVIEW intervention (WP1) is supported by WP4 which provides the relevant psychological variables and determinants of health behaviour change for planning and executing the intervention. Moreover, WP4 provides a theoretical framework and assessment of potential confounders, moderators or mediators influencing motivation toward behaviour change, maintenance of behaviour and the expected effects on the primary and secondary outcomes. We hypothesize that self-efficacy (and by this the confidence to deal successfully with barriers), intrinsic motivation, causal attribution for weight outcomes, coping and self-regulation skills as well as social support are significant moderators which influence the effect of the life-style change.

The aim is to provide valid and reliable questionnaires and assessment methods for collecting and interpreting data on social cognitive determinants of behaviour, on social-ecological, on cultural and socio-demographic as well as socio-economic components. These instruments will be in English language (to be used at UNOTT, UNSYD and UOA) and translated were required into Danish, Dutch, Finnish, Bulgarian and Spanish.

Education, age, sex, marital status and other background demographic variables will be queried during screening (visit 1). The questionnaires related to moderators, mediators and behavioural and social environment are in general filled in during weeks 0, 8, 26, 52, 104, and 156 either online or in a paper-pencil version. Filling in the questionnaires takes approximately 90—120 min for adults.

- ➔ **SOP 18+20: QDP and Filling the questionnaires**

Table 6: Questionnaires on moderators, mediators, behaviour and social environment - adults

Level	Variables (moderators, mediators, confounders)	Instrument	Items	Questionnaire no
Environmental variables	**Recommended physical activity availability and accessibility**	Influence on Physical activity Instrument (IPAI subscale environment; Donahue, Mielenz, Sloane, Callahan & Devellis, 2006)	5	**Q-8**
	**Physical inactivity temptations**	Subscale competing demands from the Temptation to not exercise scale (Hausenblas et al. 2001)	6	**Q-9**
	**Recommended food availability and accessibility**	Self-constructed items	7	**Q-10**
	**Food temptations**	Self-constructed items	7	**Q-11**
Social/ interpersonal variables	**Social support for diet and exercise**	Social support for diet and exercise scales (Sallis, Grossmann, Pinski, Patterson & Nader, 1987)	46	**Q-12**

Level	Variables (moderators, mediators, confounders)	Instrument	Items	Questionnaire no
	**Teachable moments**	Social Readjustment Rating Scale (Holmes & Rahe, 1967)	8 + X (Filter)	**Q-13**
Individual variables	**Socio-demographic and economic status**	European Social Survey and International Social Survey (choice of items)	18	**Q-14**
	**Previous negative attempts of weight reduction**	Self-constructed items	2	**Q-15**
	**Habit strength** of physical inactivity and poor diet	Habit strength measure (Wood, Tam & Guerro Witt, 2005)	12	**Q-16**
	**Intention**	Self-constructed items (adapted from Schwarzer & Renner, 2011)	3	**Q-17**
	**Self-efficacy** (physical activity and nutrition)	The nutrition self-efficacy scale & The physical exercise self-efficacy scale (Schwarzer & Renner, 2005)	5 + 5	**Q-18**
	**Causal attributions** (for weight outcomes)	Attributional weight outcome scale (Brubacker, 1988)	15	**Q-19**
	**Self-regulation goal adjustment**	Goal Adjustment Scale (Wrosch et al., 2003)	10	**Q-20**
	**Coping** self-efficacy	Coping self-efficacy for physical activity and healthful nutrition (Schwarzer & Renner, 2000)	11+ 3	**Q-21**
	**Perceived stress**	Perceived Stress Scale (PSS; Cohen, 1983)	10	**Q-22**
	**Outcome expectancies**	Outcome expectancy of behavior change (Subscale for change of nutrition habits and subscale for exercise (Renner & Schwarzer, 2005)	12 + 13	**Q-23**
	**Self-regulation of motivation**	Treatment self-regulation questionnaire for diet & exercise (TRSQ; Levesque et al., 2007)	15 + 15	**Q-24**
Secondary endpoints	**Quality of Life**	WHOQOL-BREF (The WHOQOL Group, 1998)	26	**Q-25**
	**Work ability** [a)]	Work Ability Index (WAI; Tuomi, et al., 1998)	24	**Q-26**

a) employees

➔ **SOP 18+20: QDP and Filling the questionnaires**

Table 7: Questionnaires on moderators, mediators, behaviour and social environment - Children and Adolescents

Level	Variables (moderators, mediators, confounders)	Instrument	Items	Questionnaire no
Environmental variables	**Recommended physical activity availability and accessibility**	Influence on Physical activity Instrument (IPAI subscale environment; Donahue, Mielenz, Sloane, Callahan & Devellis, 2006)	4	**Q-C-8**
	**Physical inactivity temptations**	Subscale competing demands from the Temptation to not exercise scale (Hausenblas et al. 2001)	4	**Q-C-9**
	**Recommended food availability and accessibility**	Self-constructed items	6	**Q-C-10**
	**Food temptations**	Self-constructed items	6	**Q-C-11**
Social/ interpersonal variables	**Social support for diet and exercise**	Subscales of the Social support for diet and exercise scales (Sallis, Grossmann, Pinski, Patterson & Nader, 1987)	8	**Q-C-12**
	**Parental SES**	Family affluence scale (see Boyce et al. 2006)	4	**Q-C-13**
Individual variables	**Coping self-efficacy**	Coping self-efficacy for physical activity and healthful nutrition (slightly modified version) (Schwarzer & Renner, 2000)	12	**Q-C-14**
	**Habit strength** of physical inactivity and poor diet	Items from the Self-Report-Habit-Index (Verplanken et al. 2003)	14	**Q-C-15**
	**Outcome Expectancies**	Outcome expectancy of behavior change (Subscale for change of nutrition habits and subscale for exercise (Renner & Schwarzer, 2005)	16	**Q-C-16**
	**Perceived stress**	Subscales of the Adolescents Stress Questionnaire (ASQ, Byrne et al. 2007)	16	**Q-C-17**
	**Self-efficacy** (physical activity and nutrition)	The nutrition self-efficacy scale & The physical exercise self-efficacy scale	10	**Q-C-18**
	**Self-regulation of motivation**	Treatment self-regulation questionnaire for diet & exercise (TRSQ; Levesque, Williams, Elliot, Pickering, Bodenhamer & Finley, 2007)	26	**Q-C-19**

7.14 Assessment of sleep

WP1 is supported by WP3, where sleep and stress will be assessed. Sleep will be assessed by examining the duration of sleep and some relevant sleep disturbances (sleep onset latency, waking up during the night without being able to fall asleep again and day-time sleepiness). The Epworth Sleepiness Scale (ESS) and the Pittsburgh Sleep Quality Index (PSQI) will be used.

Sleep will be assessed in all subjects in all centres by questionnaires and accelerometers during weeks 0, 8, 26, 52, 104 and 156 (the last only in adults).

When the subjects wear the accelerometers, they also complete the questionnaires concerning sleep from the PSQI. From the accelerometers, total duration of being in bed/24 h will be monitored.

- ➔ **Q-27, Q-C-20: ESS**
- ➔ **Q-28, Q-C-21: PSQI**

In a sub-group (sub-group F) of 40 children and adolescents, age 10-18 years, sleep architecture will be assessed one night in a metabolic ward. Changes in total sleeping time, sleep latency, REM sleep and SWS will be related to the intervention related effects on insulin sensitivity, respectively body composition, waist circumference, body weight loss, body weight maintenance. UM will investigate sleep architecture with poly-somnography in this sub-cohort of 40 children. The sub-cohort will consist of 2 stratified intervention groups (20 participants in the MP and 20 participants in the HP group). Each measurement will deliver a description of duration and occurrence of total sleeping time, sleep latency, sleep phases 1 and 2, REM sleep and SWS sleep, using continuous recording with BrainRT (OSG BVBA, Rumst, Belgium). Assessments will be done in weeks 0, 52, and 104.

7.15 Assessment of food reward

A possible relationship of peripheral insulin sensitivity and brain reward activation will be assessed in a sub-group of pre-diabetic adults (sub-group E), consisting of 2 stratified intervention groups (20 MPMI, 20 HPMI).

UM will use functional magnetic resonance imaging (fMRI) in a block design with high calorie/ low calorie food images and control images. Activation will be tested in a 3T Siemens Magnetom scanner with a single headcoil.

Measurements of food reward will be done at wks 0, 26, and 104.

7.16 Assessment of stress, mood and anxiety

For stress the Perceived Stress Scale (PSS) and for mood the Profile Of Mood Scale (POMS) will be used. UM, SU and USTUTT will provide the questionnaires and analyse the data. Especially sleep and stress related changes in interaction with diet and physical activity, and in relation with changes in insulin sensitivity, body composition, and body-weight will be analysed.

Stress will be assessed in adults and children/adolescents by questionnaires during weeks 0, 8, 26, 52, 104 and 156.

Mood will be assessed in adults only by questionnaires during weeks 0, 8, 26, 52, 104, and 156.

➔ **Q-22: Perceived Stress Scale (PSS)**

➔ **Q-C-17: Perceived Stress**

➔ **Q-29: POMS**

7.17 Cost-effectiveness

The adult participants will also fill in a short questionnaire on medicine use, days absent from work, “less than optimal” workability etc. These answers are used to estimate cost-effectiveness of the life-style programs in PREVIEW.

Cost-effectiveness will be assessed in wks 0, 52, 104, 156.

➔ **Q-30: Cost-effectiveness questionnaire**

8. ADVERSE EVENTS

8.1 Definition of Adverse Events

An adverse event is any undesirable medical event occurring to participant during a clinical trial, which does not necessarily have a causal relationship with the intervention. An AE can therefore be any new unfavourable and unintended sign, including abnormal laboratory findings, symptoms, or disease, or worsening of existing symptoms, which is temporarily associated with the treatment under investigation.

8.2 Methods for Eliciting, Recording and Follow-up of Adverse Events

The participants will be asked at all CID visits, screening visit excluded, if they have noticed any unfavourable events. The AE form in the eCRF will be filled in for each separate AE. The form will cover onset, end, intensity, causality, action taken and outcome of the AE.

LCD-related AE’s will be collected and marked as LCD-related on the AE form. Common LCD-related AEs are cold intolerance (50%), gallstones (10–30%), bad breath (20–30%), fatigue, dizziness, muscle cramps, headache, gastrointestinal distress (10–20%). Common local adverse reactions are dry skin (50%) and hair loss (10%).

➔ **SOP-23a: CRF Adults**

➔ **SOP-23b: CRF Children**

8.3 Assessment of Intensity

The Investigator should for each AE reported by a study subject rate the maximum intensity of the AE. The intensity grades are defined as follows:

- Mild: The AE does not interfere with the subject’s usual function.

- Moderate: The AE interferes to some extent with the subject's usual function.
- Severe: The AE interferes significantly with the subject's usual function.

Note the distinction between the seriousness and the intensity of an AE. Severe is a measure of intensity; and a severe reaction will not be classified as serious unless it meets one of the criteria for serious events listed below.

8.4 Assessment of Causality

The Investigator should for each AE reported by a study subject rate the causality of the AE.
The causality grades are defined as described:

Definite: An AE occurring in a plausible time relationship to the trial intervention and which cannot be explained by concurrent disease or concomitant drugs or chemicals. The response to withdrawal of the treatment (de-challenge) should be clinically plausible, and the AE should recur on re-challenge.

Probable: An AE with a reasonable time relationship to the trial intervention, unlikely to be attributed to concurrent disease or concurrent drugs or chemicals, and which follows a clinically reasonable response on withdrawal (de-challenge). Re-challenge information is not required to fulfil this definition.

Possible: An AE with a reasonable time relationship to the trial intervention, but which could also be explained by concurrent disease or concomitant drugs or chemicals. Information on drug withdrawal may be lacking or unclear.

Unlikely: An AE with a temporal relationship to the trial intervention which makes a causal relationship improbable, and in which concomitant drugs, chemicals or underlying disease provide plausible explanations.

Unrelated: An AE which has clearly and incontrovertibly no temporal relationship to the trial intervention or is due to underlying/concurrent illness or effect of a concomitant drug.

8.5 Definition of Serious Adverse Events

A serious adverse event (SAE) is any untoward medical occurrence that:

a) Results in death;
b) Is life-threatening at the time of the event;
c) Requires inpatient hospitalization;
d) Results in persistent or significant disability or incapacity;
e) Is another important medical event.

The participant must be admitted to hospital in order to be considered as receiving hospital treatment. Hospitalization itself and surgical diagnostic procedures are not AEs. Hospitalization for elective surgery in a pre-existing condition that has not worsened during study participation is not an AE.

8.6 Reporting of Serious Adverse Events

If a SAE occurs, the study personnel should fill in a "Serious Adverse Event reporting form" and notify the Investigator. The Investigator should report all SAEs (whether or not considered related to study treatment) to the person authorized by the Sponsor to be responsible for this task (Prof. Mikael Fogelholm, University of Helsinki, mikael.fogelholm@helsinki.fi, mobile: +358 50 318 0302) within 24 hours after awareness of the SAE. A copy should be sent to the sponsor /coordinator of PREVIEW (Prof. Anne Raben, University of Copenhagen, ara@nexs.ku.dk). The reporting by the investigator to the Sponsor representative should cover the seriousness criteria, intensity and initial causality assessment.

The initial report of a SAE should as far as possible be supplemented by detailed information on diagnosis/symptoms, the relationship with the start of the intervention. A follow-up report of the SAE will be written as applicable and sent to the Sponsor's representative.

SAEs will be reported to the Authorities according to local regulations. If applicable, the Investigator will report to the applicable Regulatory Authorities and Ethics Committee all relevant information about SAEs, in any case no later than seven days after knowledge by the Sponsor of a case which caused death or was life-threatening; no later than 15 days after knowledge for other unexpected serious adverse reactions. The Sponsor will answer any complementary request made by the Health Authorities regarding any such event.

8.7 Follow-up of Adverse Events and Serious Adverse Events

After an AE or SAE, the subject should be followed by any clinical or biological examination, considered as necessary by the medical judgment of the Investigator, until the AE/SAE has resolved, stabilized, the investigator deems further observations or examinations to be no longer medically indicated or until the subject is under professional medical care. Follow-up should always be performed until a potential causality between the study treatment and the AE has been assessed.

Once yearly, the Ethics Officer in PREVIEW evaluates the list of all AE's and SAE's and makes a report summarising the observations. This can then be handed in to the local authorities.

8.8 Procedures in Case of Medical Emergency

The overall medical expert for the study is Prof. MD Stephen Colagiuri, University of Sydney, stephen.colagiuri@sydney.edu.au, mobile: + 61 419 432 985 , who may be consulted in case of serious adverse events. However, each study-site will nominate its own expert who takes the local medical responsibility.

- ➔ **SOP-23a: CRF Adults**
- ➔ **SOP-23b: CRF Children**

9. DATA MANAGEMENT AND QUALITY

9.1 Data quality

All data will be collected using an electronic case record form (eCRF). The eCRF is based on the electronic data capture system OpenClinica (21).

Data to be entered in the OpenClinica system from the screening examination will include those data that are collected until the time where a possible decision of non-inclusion is taken. Further, data related to medical history and concomitant medication will not be entered in OpenClinica if the participant is not included in the study.

A log will be kept on each site to document the origin of source data.

The investigator and authorized staff at the clinical centres can add data to the eCRF and must keep the eCRF current to reflect subject status during the course of the trial. The eCRFs for any subject leaving the study should be completed at the time of the final visit or shortly thereafter. The End of Trial form should be filled out giving the reason for termination (e.g. screening failure, adverse event, and diagnosis of type-2 diabetes). A screening number and the date of birth identify the subjects on the eCRF. The study personnel must make a separate confidential record of personalized details (name and initials) on the subject identification and enrolment log which is kept separately from the eCRF at each site.

Laboratory data will be entered into the eCRF, partly by double data entry. Most lab data will be entered by the central lab (THL). Paper source data will be archived for 15 years locally in each study-site after end of the study.

➔ **SOP-23a: CRF Adults**

➔ **SOP-23b: CRF Children**

➔ **SOP-23c: End of Trial/Termination**

9.2 Confidentiality

In order to maintain anonymity, subjects will only be identified by their date of birth (according to local law) and the assigned screening number for all documents associated with data collection.

All information obtained during the study will be handled according to local regulations and the European Directive 95/46/CE (Directive on protection of individuals with regard to the processing of personal data and on the free movement of such data).

9.3 Data Quality Assurance

Study specific training of study staff at the clinical centres will be performed prior to initiation of subject recruitment. The training will include as a minimum a review of all procedures to be performed at the

clinical centre, handling of laboratory samples and eCRF training. A log will be completed to document training.

Collection and preparation of all laboratory samples at the clinical centres will be performed in a standardized way according to a written laboratory manual. All laboratory supplies will be purchased locally at each site. All analyses for adults will be performed at the central laboratory (THL) to minimize variation. For children, UM will perform the analyses.

Provided extra funding is obtained, monitoring of the clinical centres will be performed during the study using a risk-based approach. The specific items to be monitored will be described in detail in a written monitoring plan.

- ➔ **SOP-17: data quality check and data entry**
- ➔ **SOP-7: Lab manual**

9.4 Statistical considerations

9.4.1 Sample size calculation

Based on previously published data among patients who completed the study, the 3-y incidence of type-2 diabetes in adult people aged 40 y with a diabetes risk score $\geq$12 is 21%. This was derived by combining overall cumulative incidence derived from results by Lindstrom *et al.* (2008) with the risk reduction reported by Tuomilehto *et al.* (2001) and Diabetes Prevention Program Research Group (2002).

We base our main power calculations on **two** intervention arms (i.e. HP vs. MP diet). For adults we hypothesize that a risk reduction of 25% in the MP group will reduce the diabetes incidence in this group to 15.8%. The estimated 25% risk reduction is based on outcomes achieved in published diabetes prevention trials and based on completer analyses. We hypothesise that the HP group will achieve an overall 50% diabetes risk reduction from a baseline risk of 21% to a 3-y risk of 10.5%.

Using these assumptions a conservative estimate of the sample size required to detect this difference in incidence (15.8% vs. 10.5%) is at least 649 per arm or 1,298 participants in total (for a two-sided comparison with a power of 80% and alpha of 0.05). We estimate a 30% drop-out rate (similar to DiOGenes) during the 148 week intervention period.

Thus, we will need at least 1,860 subjects starting the intervention. To further allow for a drop-out after inclusion and for subjects not losing 8% weight during the 8 weeks weight loss period (estimated 25%), a total of 2,472 subjects should be recruited (see Figure 2).

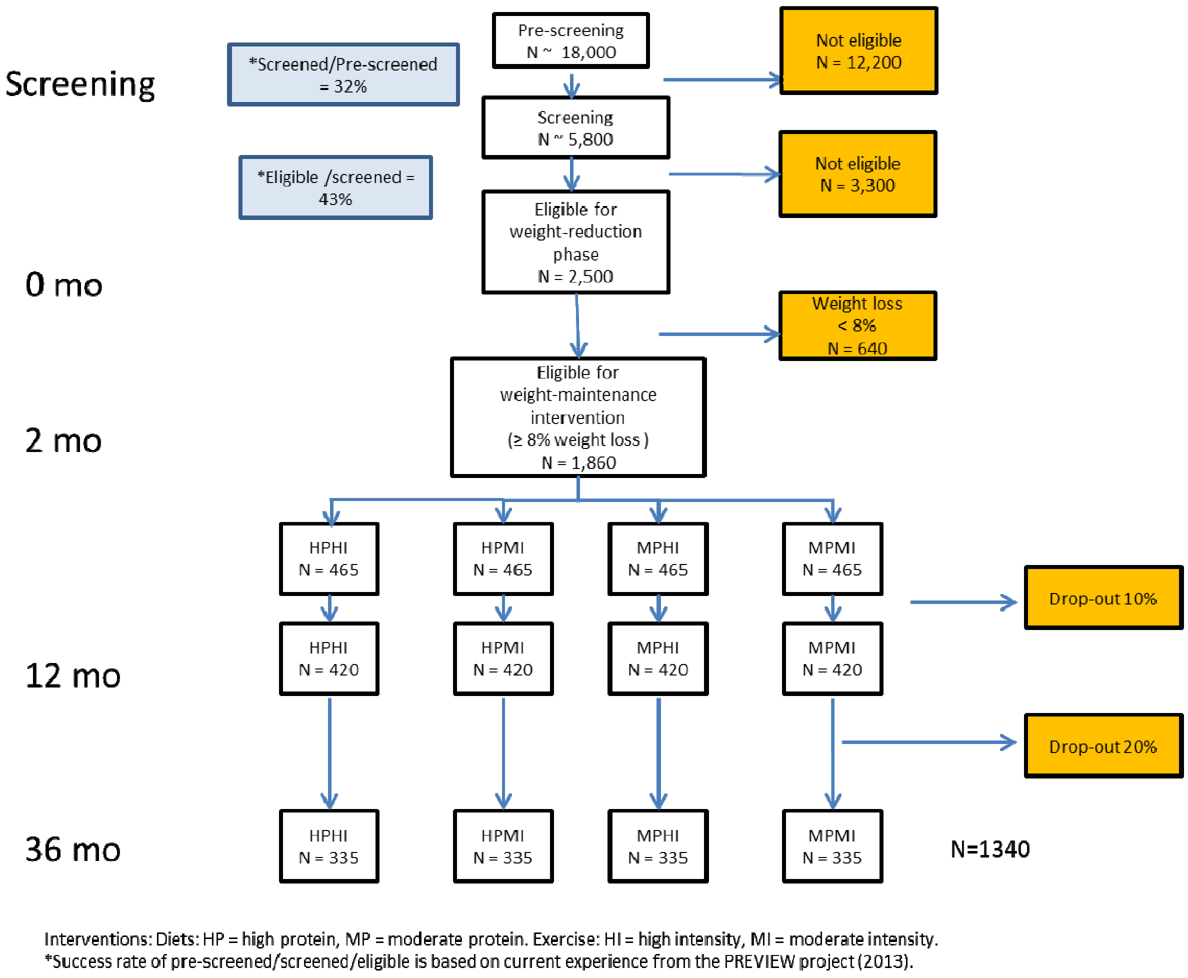

Figure 2: Participant flow during the study (presented for the 4 arms).

The Consortium will recruit up to a total of 2,500 participants at high risk of diabetes, from the 8 centres (on average 315 in each centre) to initiate the weight-reduction period.

The percentage of subjects going from pre-screening → screening → eligible is 32% and 43%, respectively. This is based on obtained experience on recruitment in the study (from June 2013 to Feb 2014). Therefore, we will pre-screen about 18,000 subjects and screen about 5,800 subjects in order to get the total number of 2,500 eligible subjects (see Figure 2).

9.4.2 Handling of missing values

The time of onset of T2D in subjects with pre-diabetes at baseline will be registered at specific visits (CIDs). If presence of diabetes is observed at a visit, the onset must have occurred between this visit and the previous visit where diabetes was examined. Thus, the observations can be considered as interval censored data. The time of onset will be set to be in between the first of the two required registrations of elevated FPG or IGT (from OGTT) or HbA1c, and the diabetes assessment visit prior to the first registration.

Since the sample size estimations take into account the drop-outs during the study, sufficient data should be obtained.

The Full Analysis data set is defined as all randomised subjects who have been participating in at least one counselling session after the entry into the randomised period, and where at least one post-baseline assessment of the primary outcome (T2D) has been performed (i.e. confirmation from own medical doctor, or as assessed during the OGTT analysis or from HbA1c).

The primary statistical analysis of the primary outcome will be done using interval censored failure time data on the Full Analysis data set. A conservative estimate of the power has been calculated as if the endpoint diabetes yes/no among completers (1,298) during the 3 years is analysed by use of a two-sided Chi-square test with a significance level of 5%.

The data will be analysed using a parametric or semi-parametric survival analysis model. The model will include effects of treatment, gender, age group and centre. Baseline FPG will be included as a covariate. From this model the hazard rate between treatments will be estimated and a test on no difference between treatments will be performed. Secondarily, a number of sensitivity analyses may be applied.

9.4.3 Power calculations on non-primary outcomes:

A secondary power calculation was done by using HbA1c as a continuous outcome variable. Based on the 3-y results from the Finnish Diabetes Prevention Study, an anticipated difference between two groups is 0.2% with an SD of 0.6%. Using an 80% power and alpha of 0.05, the estimated sample size on each treatment is 142. Allowing again for a 30% drop out, this increases the sample size to 185 per group and to 740 in total for four groups. Hence, the strict power calculation given above and used for our primary (dichotomous) outcome, leads to a sample large enough to allow for sub-group analyses of HbA1c, and possibly also for other continuous variables.

The changes in continuously distributed variables from baseline to week 26 and week 156 within treatment groups will be tested using Analysis of Covariance (ANCOVA) and adjusting for baseline value, centre, age-group and other stratification variables (all tests two-sided).

All categorical variables will be expressed in contingency tables and analysed using Fisher's exact test. Survival analysis using the Kaplan-Meier plot and the log-rank test will be used to investigate time to subject withdrawal.

All null hypotheses comparing groups assume equality between the treatment groups while the alternative hypotheses assume that there is a difference (two-sided). All within-group null hypotheses assume no change from baseline to week 156 while the alternative hypotheses assume that there is a change (two-sided).

10. ETHICAL ASPECTS

10.1 General

The PREVIEW Consortium gives the highest priority to the ethics issues that arise in this project. The work of PREVIEW will be carried out in full compliance with the relevant requirements of the latest version of the Declaration of Helsinki (59th WMA General Assembly, Seoul, Korea, October 2008), and the ICH-GCP, The International Conference on Harmonisation (ICH) for Good Clinical Practice to the extent that this is possible and relevant. In addition, all national laws and regulations of the local ethical committee regarding human research will be strictly enforced. The study will start up in each country when the local ethical committee approval is obtained.

PREVIEW includes research involving pre-diabetic overweight human beings voluntarily enrolled in a multicentre trial running for 3 years in adults and for 2 years in children and adolescents (WP1). Human material in terms of biological samples (e.g. blood, urine, faeces) as well as personal data will be collected during the multicentre trial. Most biological samples will be analysed centrally at a central laboratory at the National Institute for Health and Welfare (THL) in Finland. The personal data will include aspects of health, ethnicity and information related to lifestyle variables such as dietary preferences and habits and physical activity habits as well as sleep, stress, habitual behaviour, social environmental influences, cultural habits, socio-ecologic and socio-economic. The project will not collect data on political opinions, or religious or philosophical convictions. None of the data collected will be disclosed to any third party except for auditing and/or monitoring by the appropriate regulatory authority and the information collected will only be used within the project.

The partners in the PREVIEW project fully conform to national legislation and applicable codes of conduct and seek the approval of the relevant ethics committee prior to start of the research activities (99/167/EC: Council Decision of 25/1/99). Ethical issues arising at local levels will be monitored by the site-PI. The monitoring of the relevant ethical issues will be on the agenda of Steering Committee meetings and the General Assembly of PREVIEW. It will monitor compliance with European Commission project standards at all intervention centres and will support ethics capacity where necessary.

The PREVIEW Consortium will ensure that all partners fulfil their ethical obligations. The laboratories handling blood and urine will fulfil local and national safety requirements. Utilization of chemicals in this project will be subject to Institutional and National safety regulations to ensure the safety of employees and to prevent damage to the environment both in the European community and elsewhere. People handling biohazardous material will do so only after they have received proper training. Disposal of hazardous materials will be performed according to EC-regulations. The compliance with these rules is supervised by institutional safety officers.

The PREVIEW Coordinator will be advised on any ethical issues by a GCP expert at UCPH who is at all times up-to-date with the latest rules and guidelines. The GCP expert will be consulted whenever new events require a significant change of the protocol in the course of the studies in PREVIEW.

Further, an independent external advisor with expertise in research ethics in clinical trials has been appointed within the project's Scientific Advisory Board, namely Prof. Richard Atkinson, USA.

10.2 Participant information and Informed Consent

Written, informed consent will be obtained from all participants prior to any procedures related to the protocol. The Investigator or delegated study personnel with proper qualifications will explain verbally to each subject the objectives, nature, significance, risks and implications of the study before inclusion. This information will also be included in the written subject information sheet.

In particular, the participants will be informed about the following:

- The possibility of withdrawing from the study at any time without losing any benefits the participant is entitled to;
- How personal and health-related data will be collected and used during the study;
- That all personal and health-related data will be pseudo-anonymized;
- How the identifiable data (name, home address, etc.) and pseudo-anonymized research data are coupled, and how the identifiable data are stored.

The participants together with an assessor will be given time to discuss any questions and if needed in a private enclosed room and make a decision regarding participation in the study. All participants will receive a copy of the subject information sheet and the signed informed consent form (ICF). The original will be retained by the Investigator.

No systematic deviations from the protocol are allowed, and no protocol waivers will be given. All protocol deviations noted during the study (whether by the Investigator, the monitor or the sponsor) will be recorded and evaluated as major or minor before the database is closed.

The Ethics Committee must also be consulted whenever new events require a significant change in the protocol or its appendices during the study. The initial Ethical Review is required for the 3-y study (or 2-y for children/adolescents). If there is a follow-up, a new review will be needed for this. This option is, however, clearly indicated both for the participants and for the Ethical Committees.

Informed consent for children must be procured from the person(s) having custody. If both parents have custody, consent must be obtained from both of them. Consent must also be obtained from the child itself, if this is not excluded by circumstances. An assessment will be made as to whether the child is mature enough to understand what the experiment involves and what consent means. Even so, the experiment may not be conducted if the child objects to it. If a child is unwilling to undergo (one of) the investigative procedure(s), the investigation will be cancelled, despite initial approval by the parents. The parents will be informed about this in advance and have to agree with this behavioural code. Parents can withdraw their approval of the study of their child(ren) at any moment.

As explained above, all participants are free to leave the study, even without explaining the reason, at any time. National ethical guidelines may have different principles on how the data collected from these participants can be used. It is likely, however, that ethical boards could approve a principle that the data for participants leaving the study can be used after asking written permission to do so.

10.3 Medical safety

Protocols of the same kind have been used previously, also with participants who are overweight or obese and who have increased risk-factor levels. The data collection includes blood specimen draws, and the only risk associated with this is a light pain at the needle insert, and possibly a small bruise around the site of insertion, which will disappear within 1-2 days. The total amount of blood drawn is less than 100 ml at each CID.

Some methods will induce minor radiation (e.g. DXA). Therefore, fertile women will be tested for pregnancy and excluded if the test is positive.

The health hazards of these methods are considered negligible, since the methods are routinely used in both research and in clinical practice. For the oldest age-group, we will select individuals who are pre-diabetics, but who are capable of participating in an exercise-intervention. This inclusion criteria will exclude the most fragile and vulnerable participants.

All study sites will have at least one physician who can be consulted in case of medical uncertainties.

10.4 Children's participation

The inclusion of obese and overweight children in the intervention is expected to produce a health benefit also for them, although the primary endpoint (incidence of type-2 diabetes) is not a likely event. In PREVIEW, children are studied in UM, UNAV, and SU.

Children and adolescents enrolled in the project will receive information sheets where the aim and the background of the study are briefly explained and their parents will be asked for consent. Even when the child is able to give consent, the investigator must also obtain that consent from the parents. The definition of legal representative will be in accordance with the legislation of the host country. If the child reaches the age of maturity in the period of the study, the research team will obtain his/her consent to continue the study including use of samples. If a child refuses to participate or continue participating in the study it will be fully respected. The scientists will avoid exerting any pressure against the child or his/her parents that will lead to the participation of the child in the project.

Even though children are a vulnerable group, studies involving children are necessary to progress the well-being, prevention and necessary treatment for this age group as well as for the adult. Special attention will be paid to minimize the burden for the children in the PREVIEW project and to the potential risks associated with a high-protein diet in children in particular, with respect to growth and metabolism. SOPs will describe what outcome measurements will be taken in children, including physical measurements and blood samples, and how to deal with potential adverse events during the trial. The Consortium will minimize the measurements involved for the children. However, it will be necessary to take some blood samples in order to fully address the safety aspect of the diet also in this group. This will need to be performed in a sensitive manner to assure cooperation by the children and their parents.

The inclusion of children in the multi-centre intervention is expected to produce a health benefit in terms of weight loss or reduced weight gain. The Consortium believes it is important to include the children in the intervention because the Consortium wants to investigate the different lifestyle variables

over the whole life span. The Consortium also believes that parents should consider the multi-centre trial of potential benefit to their children with respect to the prevention of diabetes. It is important to underline that the high-protein diet does not provide more than 25% energy from protein, which is less than for certain popular diets (e.g. Atkins, the Zone).

The children undergoing the intervention will benefit in several ways. The children will take part in a weight management program that is new, challenging, and addresses other components than only a diet. In addition to the diet they will follow advices on an age-related physical activity program, consisting of structured medium and high-intensity physical activity times throughout the day. Moreover, children and their parents will receive information and instructions on sleep hygiene (bed-times, duration in bed, no lights on, no music, no TV in the room etc.) and possibly experience that this may be a very easy way to underscore body weight management. Their overall benefit is a better body weight management leading to reversal to normal from their pre-diabetic state supported by neural changes going along with improvements in insulin sensitivity. If successful, the program will be made available and disseminated for children in a similar situation. Otherwise, children get the same benefits as the adults, that is, they will receive personal feedback on their responses to an intervention which may reduce their future susceptibility to type-2 diabetes.

Underage children (the age may be dependent on country, usually below 18 y) are only allowed to enter the study after an Informed Consent by his/her parent or legal guardian (see 10.2).

10.5 Privacy of participants

All data collected in this study are confidential and every effort will be made to affirm and uphold the principle of the subject's right to protection against invasion of privacy. The measurements will be done in a private setting (room, etc.), with only one participant present at a time. No personal data are to be discussed at the group meetings. The data are entered in the central data hub by using participants ID-codes.

The privacy of personal data will be protected in accordance with national and EU regulations. Security measures will protect the personal data. These include passwords for databases and controlled access to buildings. The handling, storage and transfer of data will be conducted in accordance with the Data Protection Act (DPA) 1998, and if the data is transferred outside European this will be performed in accordance with Schedule8, DPA 1998.

10.6 Participant's right to their own results

The volunteers will eventually get personal feedback on the main findings, like blood pressure, cholesterol levels, insulin resistance and body composition. This feedback is done after all results have been analysed and it is restricted to data with results that can be clinically interpreted. More scientific data (i.e. data where group level information may be meaningful, but personal data cannot be interpreted in a reliable way) are given as group results, in a seminar-type session, so that the participant can also get answers to their questions. In general, volunteers are entitled to receiving information on their personal results at the end of the study. If any disease progression or serious side-effects are noted during the

study, the general practitioner of the participant will be contacted in order for him/her to discuss the event and any actions with the volunteer.

10.7 Insurance

All subjects will be insured against injury caused by their participation in the study according to local legal requirements in the Countries where the study takes place.

10.8 Reimbursement

In principle, PREVIEW participants are not given any reimbursement for participation. However, a possible reimbursement for the participants is up to the individual study sites. Moreover, reimbursement of travel expenses in certain circumstances may be considered. The amount of the reimbursement must not be able to influence informed consent, i.e. choice to participate.

11. TRANSLATIONS

When needed (i.e. in the non-English speaking countries), documents are translated into national language. The translations are done by certified translators, in cooperation with the site-PI. Where relevant the texts are translated back to English by another certified translator. All versions (original, translation, back-translation) are stored at least at the coordinating centre (UCPH).

12. SCIENTIFIC PUBLICATIONS

After completion of the study, the results, both positive as well as negative, will be tabulated, evaluated and issued as a complete final clinical study report. A summary of the report will be sent to the Independent Ethics Committees (IECs) and Regulatory Authorities according to the applicable regulations.

Scientific publication is primarily the responsibility of the PREVIEW "Publication Advisory Group" (PAG). Main results from the intervention study must be published before breaking of the diet and exercise code can be performed. Thus, all publications and data analyses prior to the main results will be performed without breaking the intervention code. Furthermore, publications with site-specific data will not appear, until these data have been published in a core publication using data from all interventions sites.

All suggestions for publications must be approved by the PAG before data can be used. Authorship will be included according to the Vancouver Declaration. Reports will be published in relevant international and national peer-reviewed scientific journals, non-scientific journals or other relevant media. A set of Publications Rules define the principles for use of data and for authorship for all involved, including undergraduate, PhD, and post doc students. Further, rules for possible site-specific analyses are defined.

The study-sites are allowed to carry out ancillary studies, either in one site only or as a joint study of 2 or more sites. The main principle is that any additional research outside the original PREVIEW protocol must never interfere with the original protocol. The sites are also required to get their own funding for

these studies. All sub-studies need to be applied for and approved by the Steering Committee in PREVIEW. Only approved sub-studies can be conducted using the PREVIEW subjects.

The trial has been registered at www.clinicaltrials.gov with **ClinicalTrials.gov identifier:** NCT01777893.

13. FINANCIAL CONDITIONS

The European partners in the PREVEW project are financed by 8.99 mio Euro from the 7th EU framework KBBE.2012.2.2.-03 "Impact of lifestyle on well-being and diet-related disease". About 70% of this budget is allocated to the clinical intervention study described here (WP1).

For all EU partners with a budget above 375,000 Euro, an audit will be performed one or more times.

The funding for the Australian and New Zealand partners derives from national research programmes: The National Health and Medical Research Council - EU Collaborative Grant, AUS, and the University of Sydney. The NZ Health Research Council (14/191), and the UoA Faculty Research Development Fund.

The Cambridge Weight Plan is supporting the project by sponsoring the low calorie diet (LCD) formulas for all adult participants.

14. TIME-SCHEDULE

The proposed time-schedule for the main events in this study is summarised in Table 8.

Table 8: Time schedule for PREVIEW intervention study

Task	Timing
Preparatory phase: development of the interventions, protocol description, instruction materials, electronic case record forms (eCRF), training of the staff in the different intervention centres, production of specific standard operation procedures (SOP)	1/2013-6/2013
Ethical approval for the RCT in each participating country	3/2013-9/2013
Recruitment and screening of pre-diabetic subjects, adults<br>Recruitment and screening of children and adolescents	6/2013-2/2015<br>6/2013-5/2015
Workshops given to the diet and physical activity instructors	6/2013-10/2013
Weight-reduction phase (8 weeks) including data collection, site specific data analyses and randomization, Adults:<br>Children and adolescents:	8/2013-6/2015<br>10/2013-7/2015
Weight-maintenance intervention, including guidance (diet and physical activity) and data collection. Adults:<br>Children and adolescents:	11/2013-3/2018<br>12/2013-5/2017
Carrying out the dietary intake data analyses, adults<br>Carrying out the dietary intake data analyses, children/adolescents	1/2015-9/2018<br>1/2015-6/2017

Task	Timing
Carrying out the lab analyses, adults<br>Carrying out the lab analyses, children and adolescents	6/2015-9/2018<br>1/2015-6/2017
Evaluation of urine metabolomics (metabolic profile) in a sub-group	6/2015-9/2018
Evaluation of colon cancer risk markers (phenolic metabolites, SCFA, nitrogenous compounds) will be analysed from a sub-group by using 3-d faecal samples	5/2016-9/2018
Carrying out the statistical analyses and drafting the manuscript in children/adolescents	5/2015-12/2017
Carrying out the statistical analyses and drafting the manuscript in adults	5/2018-12/2018

15. RESEARCH TEAM AND RESPONSIBILITIES

The main academic researchers are described in Table 9.

Table 9 – PREVIEW research team

Partner nr. (according to DoW)	Study site/country	Name and title	Tasks in PREVIEW intervention
**2**	**Helsinki, FI**	Mikael Fogelholm, Professor<br>Kirsi Pietiläinen, Associate Professor, MD<br><br>Heikki Tikkanen, Professor, MD, DMSci<br><br>Elli Hovi, M.Sc. (nutr)<br>Saara Kettunen, M.Sc. (clin nutr)	Principle PI of PREVIEW WP1 intervention<br>Medical consultant, co-investigator, and responsible for RNA sampling<br>Medical consultant, Specialist in Clinical Physiology, Sports and Exercise Medicine<br>Site Coordinator<br>Ph.D. student, main responsible for dietary issues and gut microbiota
**1**	**Copenhagen, DK**	Anne Raben, Professor<br>Thomas Meinert Larsen, Assoc.Professor<br>Pia Christensen, Post doc<br>Christian Ritz, statistician<br>Finn Sandø-Petersen, data manager<br>Lars Dragsted, Professor<br>Lesli Larsen, Assoc. Prof.<br>Lene Stevner, BSc<br>Lone Vestergaard Nielsen<br>Grith Møller Poulsen	Coordinator of PREVIEW<br>Site-PI of PREVIEW intervention<br>Daily responsible for intervention study<br>Responsible for statistical analyses<br>Responsible for datahub and eCRF<br>Responsible for metabolomics<br>Responsible for DNA sampling.<br>GCP-coordinator<br>Assistant project coordinator, PhD student<br>Assistant project coordinator, PhD student
**5**	**Nottingham (site)**	Ian A Macdonald, Prof.<br>Peter I Mansell, Prof.<br>Elizabeth J Simpson, Dr.<br><br><br><br>Moira A Taylor, Dr.	Site PI of PREVIEW intervention<br>Senior Medical responsible<br>Researcher and Reg Nurse, Site coordinator, laboratory work Intervention coordinator and group instructor (Diet)<br>Associate Prof and Reg Dietician
**6**	**Navarra (site)**	J. Alfredo Martínez, Prof.<br>Santiago Navas-Carretero, Res. Assoc. PhD<br>Itziar Abete, Research Associate, PhD	Site-PI of PREVIEW intervention<br>Daily responsible for intervention study<br>Participation in intervention study and laboratory work
**4**	**Maastricht (site)**	Margriet Westerterp-Plantenga, Professor	Site-PI of PREVIEW intervention, WP3 leader

Partner nr. (according to DoW)	Study site/country	Name and title	Tasks in PREVIEW intervention
		Anita Vreugdenhill, MD, PhD<br>Tanja Adam, PhD<br>Mathijs Drummenm M Sc,<br>Elke Dorebos, Msc, Md<br>Jesse Rijks, MSc, MD	Paediatric responsible, co-investigator<br>Adult intervention, co-investigator<br>PhD-student carrying out the study in adults<br>PhD students carrying out the study in chil-dren and adolscents
**7**	**Sofia (site)**	Svetoslav Handjiev, Prof., MD, PhD<br>Nadka Boyadjieva, Prof., MD, PhD<br>Teodora Handjieva-Darlenska, Ass. Prof, MD, PhD	Site-PI of PREVIEW intervention<br>Co-investigator<br>Co-investigator
**13**	**Sydney (site)**	Jennie Brand-Miller, Prof, PhD<br>Stephen Colagiuri, Prof, MD<br><br>Ros Muirhead Ph.D.<br>Tania Markovic MD,PhD, Ass.Prof<br>Ros Muirhead Ph.D<br><br>Amanda Salis, Assoc.Prof.PhD<br><br>Radhika Seimon PhD<br>Shannon Brodie<br>Jessica Honeywood<br>Kylie Simpson BExerSportSci	Site-PI of PREVIEW intervention, WP5 leader<br>Endocrinologist and chief medical consultant for PREVIEW at UNSYD<br>Research Dietitian<br>Clinician<br>Clinical Trial Coordinator and Research Dieti-tian<br>Associate Professor, leading sub-study on appetite, bone health, muscle strength<br>Post-doctoral fellow, sub-study<br>Research dietitian<br>Research dietitian<br>Exercice physiologist, Phd student
**14**	**Auckland (site)**	Sally Poppitt, Prof<br>Anne Thea McGill<br><br>Nick Gant, Senior Lecturer, PhD<br>Amy Lieu, Dietician<br>Marta Silvestre, Post-doc	Site-PI of PREVIEW intervention<br>Clinician and medical consultant for PRE-VIEW at UoA<br>Associate investigator - exercise<br>Dietary intervention<br>Daily responsible for intervention study
**9**	**Stuttgart (academic partner)**	Wolfgang Schlicht, Prof<br>Daniela Kahlert, PhD<br>Annelie Reicherz, Research Assistant	Principle PI of WP4<br>Co-investigator<br>Co-investigator
**8**	**Swansea (academic partner)**	Gareth Stratton, Prof<br><br>Melitta McNarry, PhD, Lecturer<br><br>Kelly Mackintosh, PhD, Lecturer<br><br>Sinead Brophy, PhD, Associate Profes-sor/Reader<br>Jeff Stephens, PhD, MD, Diabetologist<br>Nils Swindell, Ph.D. student<br>Masoumeh Minou, PhD, Res assistant<br><br>Chris Bidder<br>Parvaiz Ali, Prof, Head of Nuclear in Sin-gleton Hospital (Medical Physics Expert)<br>Rhod Evans, Associate Prof (Clinical Radia-tion Expert)	Responsible for planning and assessment of physical activity and analysis of sleep data<br>Co-Investigator: fitness and activity pro-gramming WP3<br>Co-investigator: Physical activity measure-ments and Behaviour change<br>Co-investigator: Biostatistics and analysis of data in WP3<br>Advisor, Consultant Physician.<br>Responsible for children study in SU<br>Responsible for coordination and adminis-tartion of the research project<br>Paediatrician<br>Advisor on the DEXA and body composition measurement<br>Medical Advisor on DEXA scan
**12**	**THL (central lab)**	Jouko Sundvall, MSc	Responsible for lab manual, central lab sam-ples and analyses

**Partner nr. (according to DoW)**	**Study site/country**	**Name and title**	**Tasks in PREVIEW intervention**
		Laura Lund	Co-responsible

16. LIST OF INSTRUCTION PAPERS, QUESTIONNAIRES AND SOP'S

16.1 Instruction papers

Ins-1: Instruction to participants regarding blood specimen draw and OGTT

Ins-2: Instruction to participants regarding blood pressure assessment

Ins-3: Instruction to participants regarding urine sample collection

Ins-4: Instructions to participants on collecting faeces

Ins-5: Instruction to participants for filling in food diaries

Ins-6: Instructions to participants for using accelerometers

Ins-7: Instructions to participants regarding physical fitness tests (VO_2 max)

Ins-8: Cooking books (HP and MP diets)

Ins-9: PA booklets

Ins-10: Instructions to participants regarding the Low Calorie Diet (LCD) (adults)

Ins-11: Children diet during LCD

Ins-12: Re-feeding strategy

Ins-13: Physical Activity Log

16.2 Questionnaires

16.2.1 Adults

Q-1: Screening interview

Q-2: Background-data questionnaire

Q-3: Dietary Compliance Questionnaire

Q-4: TFEQ

Q-5: VAS for assessment of appetite

Q-6: Baecke questionnaire

Q-7: Physical activity log

Q-8: Recommended physical activity availability and accessibility

Q-9: Physical inactivity temptations

Q-10: Recommended availability and accessibility

Q-11: Food temptations

Q-12: Social support for diet and exercise

Q-13: Social Readjustment scale

Q-14: Socio-demographic and economic status

Q-15: Previous negative attempts of weight reduction

Q-16: Habit strength measure

Q-17: Intention

Q-18: Nutrition self-efficacy and physical activity self-efficacy scale

Q-19: Attributional weight outcome scale

Q-20: Goal Adjustment Scale

Q-21: Coping self-efficacy for physical activity and healthful nutrition

Q-22: Perceived Stress Scale (PSS)

Q-23: Outcome expectancy of behaviour change

Q-24: Treatment self-regulation questionnaire for diet and exercise

Q-25: WHOQOL-BREF

Q-26: Work ability indexQ-27: ESS

Q-28: PSQI

Q-29: POMS

Q-30: Cost-effectiveness questionnaire

16.2.2 Children and adolescents

Q-C-1: Screening interview

Q-C-2: Background-data questionnaire

Q-C-3: Dietary Compliance Questionnaire

Q-C-4: TFEQ

Q-C-5: VAS for assessment of appetite

Q-C-6: Baecke questionnaire

Q-C-7: Physical activity log

Q-C-8: Recommended physical activity availability and accessibility

Q-C-9: Physical inactivity temptations

Q-C-10: Recommended food availability and accessibility

Q-C-11: Food temptations

Q-C-12: Social support for diet and exercise

Q-C-13: Parental SES

Q-C-14: Coping self-efficacy

Q-C-15: Habit strength of physical inactivity and poor diet

Q-C-16: Outcome expectancies

Q-C-17: Perceived stress

Q-C-18: Self-efficacy (physical activity and nutrition)

Q-C-19: Self-regulation of motivation

Q-C-20: ESS

Q-C-21: PSQI

16.3 SOP's

SOP-1: LCD

SOP-2: Diets

SOP-3: Group supervision (adults)

SOP-3a: Group supervision (children)

SOP-4: Physical activity programs

SOP-4a: Physical Activity Log

SOP-5a: Pre-screening and screening (adults)

SOP-5b: Pre-screening and screening (children)

SOP-5c: OGTT during Screening

SOP-5d: Guidance Type-2 diabetes diagnose in PREVIEW study

SOP-6a: Body weight and height, sitting height, waist, hip and thigh circumference

SOP-6b: Body composition

SOP-6c: Liver fat content

SOP-7: Lab manual

SOP-8: Blood pressure and heart rate assessment

SOP-9: ECG (Electrocardiography)

SOP-10a: Collection of DNA samples

SOP-10b: Collection of RNA samples

SOP-11: Urine sample collection, analysis and handling

SOP-12: Faecal sample collection and handling

SOP-13: Diet analysis

SOP-14: VAS for assessment of appetite

SOP-15: Accelerometer data collection

SOP-16: Assessment of physical fitness (VO_2 max)

SOP-17: Data quality check and data entry

SOP-18+SOP-20: QDP + Filling the questionnaires

SOP-19: Randomization

SOP-21: OGTT

SOP-22: Children diet during "LCD"

SOP-23a: CRF Adults

SOP-23b: CRF Children

SOP-23c: End of trial/Termination

SOP-24: Concomitant medication

16.4 Cooking books

Cooking book: HP diet (Ins-8a)

Cooking book: MP diet (Ins-8b)

17. REFERENCES

1 WHO. http://www.who.int/features/factfiles/diabetes/en/index.html (accessed 20.1.2013)

2 Hu FB, Manson JE, Stampfer MJ, et al. New Eng J Med, 2001;345(11):790-7.

3 Kuusisto J, Laakso M. Curr Cardiol Rep 2013; 15:331.

4 Wang YC, McPherson K, Marsh T, et al. Lancet, 2011; 378:815–25.

5 Swinburn BA, Sacks G, Hall KD, et al. Lancet, 2011; 378:804–14.

6 Kobayashi D, Takahashi O, Deshpande GA, et al. Sleep Breath, 2012;16: p. 829-33

7 Fogelholm M, Kronholm E, Kukkonen-Harjula K, et al. Int J Obes (Lond), 2007; 31:1713–21.

8 Lindström J, Ilanne-Parikka P, Peltonen M, et al. Lancet, 2006;68:1673–9.

9 Li G, Zhang P, Wang J, et al. Lancet, 2008;371:1783–9.

10 Larsen TM, Dalskov S-M, van Baak M, et al. N Engl J Med, 2010; 363:2102–13.

11 American Diabetes Association, et al. Diabetes Care 2008;31 Suppl 1:S61-78.

12 Diabetes UK Nutrition. http://www.diabetes.org.uk/nutrition-guidelines (20.1.2013)

13 Ilanne-Parikka P, Laaksonen DE, Eriksson JG, et al. Diabetes Care, 2010; 33:1610–7.

14 Gill JMR, Cooper AR. Sports Med, 2008; 38:807–24.

15 Hayes C, Kriska A. J Am Diet Assoc, 2008; 108(4 Suppl 1):S19–23.

16 Sartor F, de Morree HM, Matschke V, et al. Eur. J. Appl. Physiol, 2010;110:893–903.

17 Kerksick CM, Wismann-Bunn J, Fogt D, et al. Nutr J, 2010;:59.

18 Farag YM, Gaballa MR. Nephrol Dial Transplant, 2011;28-35.

19 CDC. http://www.cdc.gov/physicalactivity/everyone/guidelines/adults.html. (20.1.2013)

20 Saaristo T, Peltonen M, Lindström J et al. Diab Vasc Dis Res 2005;2:67-72.

21 OpenClinica https://openclinica.com/

APPENDICES

Appendix 1: Study Flow Chart - Adults.

	Pre-screening	Screen-ing	Base-line	Weight-reduction (LCD)				Weight-maintenance intervention (four intervention groups)												
**Visit**		**1**	**2 CID1**	**3**	**4**	**5**	**6 CID2**	**7**	**8**	**9**	**10**	**11 CID3**	**12**	**13**	**14 CID4**	**15**	**16 CID5**	**17 CID6**	**18**	**19 CID7/EOT**
**Timing (month)**			**0**				**2**		**3**	**4**	**5**	**6**	**8**	**10**	**12**	**15**	**18**	**24**	**30**	**36**
**Timing (week)**			**0**	**2**	**4**	**6**	**8**	**10**	**12**	**16**	**20**	**26**	**32**	**44**	**52**	**64**	**78**	**104**	**130**	**156**
Signing Informed Consent		(-1) X																		
Inclusion/exclusion criteria	X	X																		
Demogr,.Diabetes Risk Score, Med hist. etc.	X	X	X																	
Randomization		X																		
Group meeting			X[#]	X	X	X	X[#]	X	X	X	X	X[#]	X	X	X[#]	X	X	X[#]	X	
Body weight and height*, sitting height once		X*	X	X	X	X	X	X	X	X	X	X	X	X	X	X	X	X	X	X*
Waist, hip, thigh circumference			X				X					X			X		X	X		X
Body composition			X				X					X			X			X		X
Blood pressure		X	X				X					X			X		X	X		X
Heart rate			X				X					X			X		X	X		X
ECG (55-70 y)		X																		
Fasting blood samples[+]		X	X				X					X			X		X	X		X
OGTT (0, 30, 60, 90 and 120 minutes)		0+120 min	X									X			X			X		X
DNA and RNA samples (in sites where possible)			X												X					X
24h urine collection for nitrogen/urea			X									X			X			X		X
PA and sleep – 7 d accelerometer			X									X			X			X		X
Adverse events & concomitant medication			X				X					X			X		X	X		X

	Pre-screening	Screen-ing	Base-line	Weight-reduction (LCD)				Weight-maintenance intervention (four intervention groups)												
**Visit**		**1**	**2 CID1**	**3**	**4**	**5**	**6 CID2**	**7**	**8**	**9**	**10**	**11 CID3**	**12**	**13**	**14 CID4**	**15**	**16 CID5**	**17 CID6**	**18**	**19 CID7/EOT**
**Timing (month)**			**0**				**2**		**3**	**4**	**5**	**6**	**8**	**10**	**12**	**15**	**18**	**24**	**30**	**36**
**Timing (week)**			**0**	**2**	**4**	**6**	**8**	**10**	**12**	**16**	**20**	**26**	**32**	**44**	**52**	**64**	**78**	**104**	**130**	**156**
**Sub-group measurements:**																				
Liver fat content (Sub-group A: UM)			X									X						X		
3-d faecal collection (Sub-group B: HEL, UOA)			X												X					
VO_2 max (Sub-group C: MU, UCPH, UNOTT, UNAV)			X									X						X		
Metabolomics (Sub-group D: UCPH, HEL, UNOTT, UNAV)			X												X					
Food reward (Sub-group E: UM)			X									X						X		
Kidney safety (Sub-group G)			X												X					X
**Questionnaires:**																				
General background questionnaire (WP1)			X																	
Food intake: 4-day food record (WP1)			X									X			X			X		X
Dietary compliance questionnaire (WP1)												X			X			X		X
Baecke Physical Activity Q.& PA log (WP1)			X									X			X			X		X
Three-factor eating questionnaire (WP1)			X				X					X			X			X		X
Appetite scores by VAS (WP1)			X				X					X			X			X		X
Sleep by ESS and PSQI (WP3)			X				X					X			X			X		X
Stress, mood (PSS, POMS) (WP3)			X				X					X			X			X		X
All questionnaires on moderators, mediators, behav-ioural and social environment (WP4)			X				X					X			X			X		X
Quality of life; Work ability (WP4)			X												X			X		X
Cost-effectiveness (WP4)			X												X			X		X

# = group meetings may take place on another day than the measurements; * = height is measured (in addition to weight),+ = Fasting blood samples in the screening visit will be analysed locally. All other blood-samples for adults are analysed at THL.PA: Physical Activity

Appendix 2: Study Flow Chart - Children and Adolescents

	Pre-screening	Screening	Base-line	Weight-reduction (traditional diet)				Weight-maintenance intervention										
**Visit**		**1**	**2 CID1**	**3**	**4**	**5**	**6 CID2**	**7**	**8**	**9**	**10**	**11 CID3**	**12**	**13**	**14 CID4**	**15**	**16 CID5**	**17 CID6/EOT**
**Timing (month)**			**0**				**2**		**3**	**4**	**5**	**6**	**8**	**10**	**12**	**15**	**18**	**24**
**Timing (week)**			**0**	**2**	**4**	**6**	**8**	**10**	**12**	**16**	**20**	**26**	**32**	**44**	**52**	**64**	**78**	**104**
Signing Informed Consent		(-1) X																
Inclusion/exclusion criteria	X	X																
Demographics, patient history, Diabetes Risk Score, etc.		X	X															
Randomization		X																
Individual counselling			X#		X		X#		X	X	X	X#	X	X	X#	X	X	X#
Body weight, height*, sitting height*		X*	X		X		X*		X	X	X	X*	X	X	X*	X	X*	X*
Waist, hip, thigh circumference			X				X					X			X		X	X
Body composition			X				X					X			X			X
Blood pressure, heart rate			X				X					X			X		X	X
Fasting blood samples+		X	X				X					X			X		X	X
PA and sleep – 7 days accelerometer			X									X			X			X
Adverse events & concomitant medication			X				X					X			X		X	X
**Sub-group measurements:**																		
Sleep architecture (Sub-group F: UM)			X												X			X

	Pre-screening	Screening	Base-line	Weight-reduction (traditional diet)				Weight-maintenance intervention										
**Visit**		**1**	**2 CID1**	**3**	**4**	**5**	**6 CID2**	**7**	**8**	**9**	**10**	**11 CID3**	**12**	**13**	**14 CID4**	**15**	**16 CID5**	**17 CID6/EOT**
**Timing (month)**			**0**				**2**		**3**	**4**	**5**	**6**	**8**	**10**	**12**	**15**	**18**	**24**
**Timing (week)**			**0**	**2**	**4**	**6**	**8**	**10**	**12**	**16**	**20**	**26**	**32**	**44**	**52**	**64**	**78**	**104**
**Questionnaires:**																		
General background questionnaire (WP1)			X															
Food intake: 4-day food record (WP1)			X									X			X			X
Dietary compliance questionnaire (WP1)												X			X			X
Baecke Physical activity questionnaire & PA log (WP1)			X									X			X			X
Three-factor eating questionnaire (WP1)			X				X					X			X			X
Appetite scores by VAS (WP1)			X				X					X			X			X
Sleep by ESS and PSQI (WP3)			X				X					X			X			X
Perceived Stress (WP3)			X				X					X			X			X
All questionnaires on moderators, mediators, behavioural and social environment (WP4)			X				X					X			X			X

# = group meetings may take place on another day than the measurements and can be replaced by individual counselling; * = height is measured (in addition to weight). PA: Physcial activity.

+ = Fasting blood samples at the screening visit will be analysed locally. All other blood-samples for children are analysed at the lab of Clinical Chemistry at the Maastricht University Academic Hospital.
